# Tumor microenvironment characterization in head and neck cancer identifies prognostic and immunotherapeutically relevant gene signatures

**DOI:** 10.1038/s41598-020-68074-3

**Published:** 2020-07-07

**Authors:** Mengqi Huo, Ying Zhang, Zhong Chen, Suxin Zhang, Yang Bao, Tianke Li

**Affiliations:** 1 Department of Stomatology, The Fourth Hospital of Hebei Medical University, Shijiazhuang, 050011 China; 2 Department of Stomatology, The Third Hospital of Shijiazhuang City, Shijiazhuang, 050011 China; 3 School of Chinese Material Medica, Beijing University of Chinese Medicine, Beijing, 102488 China

**Keywords:** Tumour immunology, Computational biology and bioinformatics

## Abstract

The tumor microenvironment (TME) is of great clinical significance for predicting the therapeutic effect of tumors. Nonetheless, there was no systematic analysis of cellular interactions in the TME of head and neck cancer (HNSC). This study used gene expression data from 816 patients with HNSC to analyze the scores of 22 immune cells. On this basis, we have established a novel TMEscore-based prognostic risk model. The relationship between TMEscore and clinical and genomic characteristics was analyzed. The sample was divided into risk-H and risk-L groups based on the prognosis risk model of TMEscore, with significant differences in overall survival between the two groups (log rank *p* < 0.001). In terms of clinical features, the TMEscore is closely related to the T staging, Grade, and HPV. As for genomic characteristics, the genomic features of the Risk-H samples are a low expression of immune-related genes and high-frequency mutations of TP53 and CEP152. This model was validated in an external test set, in which the prognosis for Risk-H group and Risk-L group was also significantly different (log rank *p* = 0.017). A quantitative method of TME infiltration pattern is established, which may be a potential predictor of HNSC prognosis.

## Introduction

Head and neck cancer (HNSC) is one of the most common malignant tumors worldwide^1^. In most countries, HNSC patients over the age of 50 are more common. Among them, more than 90% are squamous cell carcinomas^2^. Because of the complexity of head and neck anatomy, it is difficult to perform surgery. Although significant progress has been made in the treatment of HNSC in recent years, the total global survival rate of HNSC is only 50%^3^. Surgery and radiotherapy are the standard modalities for patients with early head and neck cancer. However, when head and neck cancer is diagnosed, more than 50% of the patients are in clinical phase stage III or IV, and lose their best chance of operation. Also, for patients with recurrence after surgery, secondary surgery trauma is more dangerous. For such locally advanced patients, the prognosis is poor^4,5^.

Given the poor prognosis after standard treatment and the low level of targeted therapies in HNSC, immunotherapy is a promising additional approach. It is currently undergoing intensive research^6,7^. At the same time, some immune related parameters have also been reported to predict the prognosis of HNSC patients. These studies further show that different immune states have a significant effect on the prognosis of HNSC patients^8,9^. The sensitivity of therapeutic targets is different among patients due to the heterogeneity of tumors. Immunotherapy, therefore, has a certain selectivity for the patient population^10^. Further differentiation of the immune subtype of cancer is necessary to identify patients who may benefit from immunotherapy.

Different tumor microenvironment (TME) can induce various adverse and beneficial consequences. Immune cells are most likely to be affected by TME and can be activated to promote tumor growth and progression^11^. More and more studies have shown that TME plays an essential role in tumor progression and therapeutic response. Some studies have shown that the infiltration of a large number of immune cell into tumor tissue is strictly related to the prognosis of patients^12^. In particular, changes in the quantitative of cytotoxic T cells, helper T cells, dendritic cells (DCs), tumor-associated macrophages, mesenchymal stem cells, and associated inflammatory pathways and fibroblasts affect the prognosis of a variety of malignancies^13–18^. Indeed, it is generally believed that an individual’s immune state is too complex to be illustrated by a single immune marker. Therefore, it is urgent to integrate a large number of HNSC transcription data to construct a new immune-related prognostic factor.

At present, the study of TME has shown great potential in the treatment of solid tumors, such as melanoma, non-small cell lung cancer, renal cancer, and prostate cancer^19–22^. In She and Chen's studies, prognosis signatures based on immune genes and the corresponding immunotherapy for different immune subgroups have been proved to be feasible strategies for head and neck cancer^23,24^. Currently, there are some algorithms associated with immune scores that can be used to estimate the abundance of immune cells in TME. However, there is still a lack of research on the quantitative model of TME infiltration by these algorithms.

**Figure 1:**
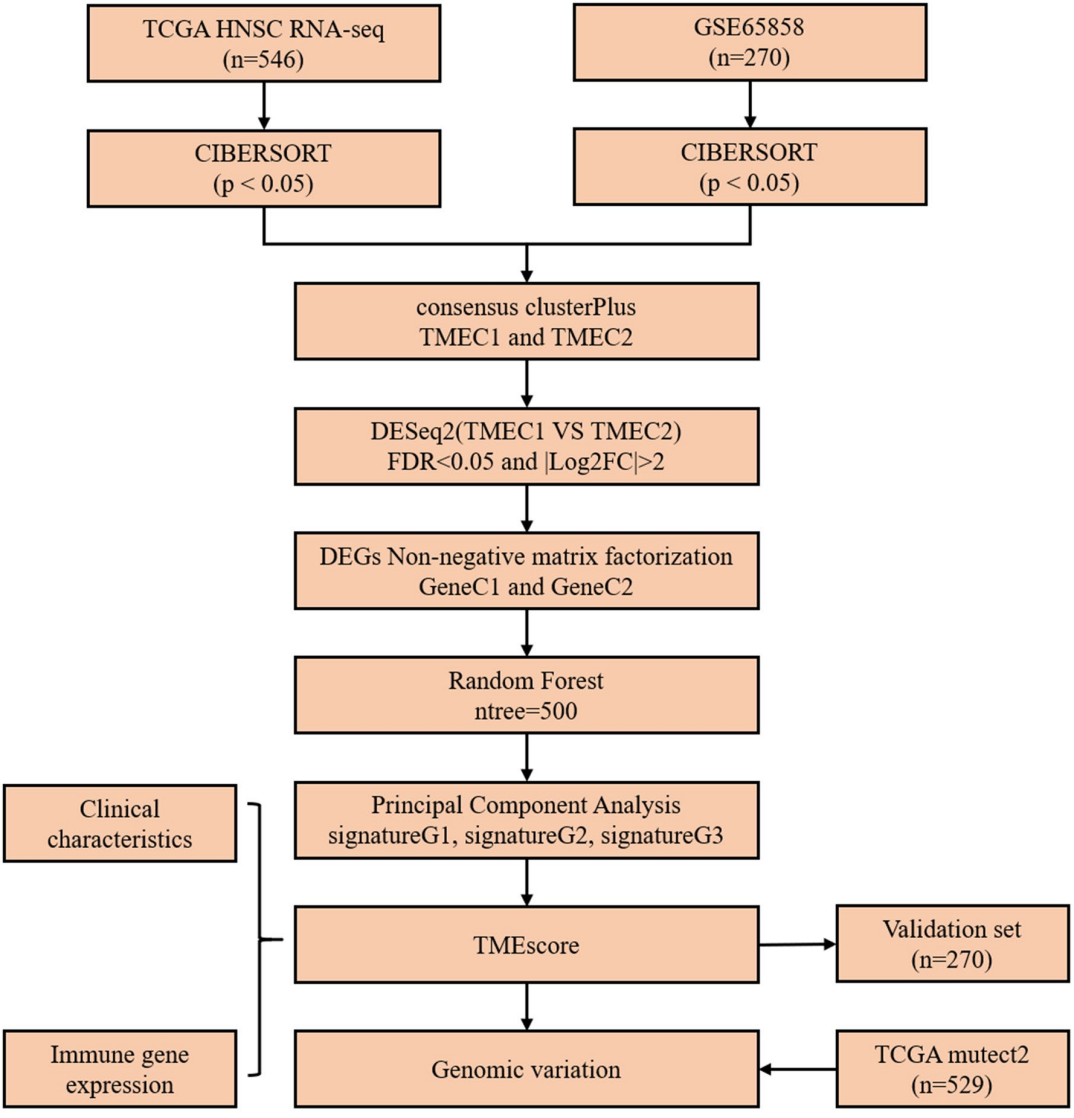
Flow chart of the experiment.

In this study, we used genetic expression data and clinical information from TCGA and GEO public databases to evaluate the proportion of 22 immune cells in HNSC TME. On this basis, the TME infiltrative patterns of 816 HNSC patients were evaluated, and the phenotypes of TME were systematically correlated with the genomic and clinical pathological characteristics of HNSC, thus establishing a quantitative method for TME infiltrative pattern. The flow chart of the experiment is shown in Fig. 1.

## Materials and methods

### Data acquisition and preprocessing

The RNA-Seq data of 546 HNSC samples were downloaded from the TCGA database on April 14, 2019. The clinical follow-up information of these samples was also downloaded at the same time. Additionally, samples without clinical data and follow-up time of less than 30 days were removed. The final study included 491 HNSC samples as training sets. Similarly, we downloaded the HNSC gene expression dataset GSE65858 from the Gene Expression Omnibus (GEO) database, which was annotated by the Illumina HumanHT-12 V4.0 expression beadchip platform. Samples with a follow-up time of less than 30 days were removed, and 270 samples were finally included as a test set. We scaled the gene expression data of the two platforms. The sample statistics of the two sets of data are shown in Table 1. For the probe data, we used the R software package Bioconductor to map the probe to Gene Symbol. Multiple probes correspond to the median expression of a gene.

**Table1:** Preprocessed clinical information of two data sets.

Characteristic		TCGA datasets (n = 491)	GSE65858 (n = 270)
Age(years)	≤ 50	88	41
	> 50	413	229
Survival status	Living	282	176
	Dead	246	94
Gender	Female	134	47
	Male	367	223
Grade	G 1	62	
	G 2	299	
	G 3	119	
	G 4	2	
pathologic_T	T 1	45	35
	T 2	132	80
	T 3	96	58
	T 4	152	97
pathologic_N	N 0	170	94
	N 1	65	32
	N 2	166	132
	N 3	7	12
pathologic_M	M 0	187	263
	M 1/ M X	62	7
Tumor Stage	Stage I	25	18
	Stage II	69	37
	Stage III	78	37
	Stage IV	261	178

### TME analysis of HNSC samples

CIBERSORT is a tool to deconvolute the expression matrix of immune cell subtypes based on the principle of linear support vector regression^25^. It can use RNA sequence data to infer the type proportion from large tumor samples with mixed cell types. To quantify the proportions of immune cells in HNSC samples, we used the CIBERSORT algorithm and the LM22 gene signature, which allows for sensitive and specific discrimination of 22 human immune cell phenotypes, including B cells, T cells, macrophages, etc. The standardized gene expression data was loaded into the CIBERSORT website (https://ciberfort.stanford.edu/). Set the threshold value to *p* < 0.05, and exclude the sample data that does not reach the threshold value. In total, 452 TCGA samples and 199 GEO samples were eligible. Scores of 22 immune cells were obtained with LM22 signature and 1,000 permutation.

### Consensus clustering for TME infiltrating cells

Consensus clustering to obtain molecular subtypes was associated with TME-infiltrating cells. Consensus clustering was performed using the ConcensusClusterPlus package in R to determine subtypes of HNSC based on TME permeabilized cells^26^. We evaluated the optimal number of clusters between k = 2–10 and repeated 1,000 times to ensure the stability of the results^27^. The cluster map was drawn using the pheatmap package in R.

### Identification and clustering of differentially expressed genes (DEGs) between TMECs

To identify genes associated with TME cell infiltration patterns, we used a linear model to analyze differentially expressed genes (DEGs) between subgroups. The gene expression data of HNSC samples in TCGA was selected for DEGs analysis of TMEC1 and TMEC2. The DEGs were calculated by DESeq2 package in R, which accorded with FDR < 0.05, | log2FC |> 2.

Nonnegative matrix factorization (NMF) is an unsupervised clustering method widely used in the discovery of genomics-based tumor molecular subtypes^28^. To further investigate the relationship between differential gene expression and the TME phenotype, we used the NMF method to re-cluster HNSC samples and analyze their clinical features. The NMF method selects "brunet" and performs 50 iterations. Set the number of clusters k to 2–10. The calculation is performed using the NMF package in R, and the minimum member of each subclass is set to 10^29^.

### Construct a prognostic risk model based on TMEscore

To obtain robust TME gene signatures, we first evaluated the prognostic value of each DEG. DEGs with significant effects on prognosis were selected, and random forest algorithms were then used to assess their importance. Genes with significant prognosis were included for randomForest feature selection using randomForest of R software package. We set the mtry for each split to be 1–279, ntree = 500. The mtry value with the lowest error rate is selected as the optimal mtry value of the random forest algorithm. Finally, the DEGs were sorted according to the degree of importance, and DEGs with cumulative importance > 95% were selected as candidate feature genes. Candidate feature genes were clustered using the k-means algorithm and then were defined as signature Gs^30^. The principal component analysis (PCA) was performed on gene expression data of each signature G using the psych package in R. After 100 iterations, the first principal component was extracted as a signature score.

The Cox multivariate regression analysis was used to establish a prognostic risk model for signature Gs. The TMEscore was calculated as follows:

1 $$ {\text{TMEscore}} = \sum {\text{PC1}}*\beta $$

Among them, β is the multifactor regression coefficient for each signature G, and PC1 is the score of the first principal component of each signature G.

### TMEscore and clinical features

To observe the relationship between TMEscore and clinical features, we divided HNSC samples into two groups by the median of TMEscore. The prognostic differences between high TMEscore and low TMEscore were compared. The relationship between TMEscore and clinical features, such as T staging, N staging, M staging, TNM stage, age, Grade, gender, smoking history and HPV was analyzed.

### TMEscore and immune gene expression

To observe the relationship between TMEscore and immune-related gene expression, we collected three types of immune-related genes. (1) Immune-stimulating genes, including CXCL10, CXCL9, GZMA, GZMB, PRF1, IFNG, TBX2, TNF, CD8A. (2) Immunological checkpoint genes, including PDCD1, CTLA4, LAG3, PDCD1LG2, IDO1, CD274, HAVCR23. (3) Activation genes of the TGF/EMT pathway, including VIM, ACTA2, COL4A1, TGFBR2, ZEB1, CLDN3, SMAD9, TWIST1. The expression profiles of these genes were extracted to further analyze the differential expression of these three types of genes in high TMEscore and low TMEscore.

### TMEscore and genomic variation

To observe differences in genomic variation between high TMEscore and low TMEscore samples, we downloaded SNP data from TCGA to remove the intron and silent mutations. Genes for differential mutations in the two samples were analyzed using fisher's exact test with a threshold of *p* < 0.05.

### Statistical analysis

The Shapiro–Wilk normality test was used to test the normality of the variables unless otherwise stated^31^. For the comparison of the two groups, the normal distribution variables were analyzed by the unpaired student t-test, and the non-normal distribution variables were analyzed by the Mann–Whitney U test. For comparisons of more than two groups, the Kruskal–Wallis test and the one-way ANOVA were used as non-parametric and parametric test methods, respectively^32^. Correlation coefficients were computed by Spearmanand distance correlation analysis. Two-sided Fisher exact tests were used to analyze contingency tables. We used the Benjamini–Hochberg method to convert P values to FDR. The Kaplan–Meier method was used to generate survival curves for each subgroup. The log-rank test was used to determine the statistical significance of the difference, and the significance was defined as *p* < 0.05. All of these analyses were performed in R 3.4.3, based on default parameters unless otherwise stated.

## Results

### TME landscape in HNSC

**Figure 2:**
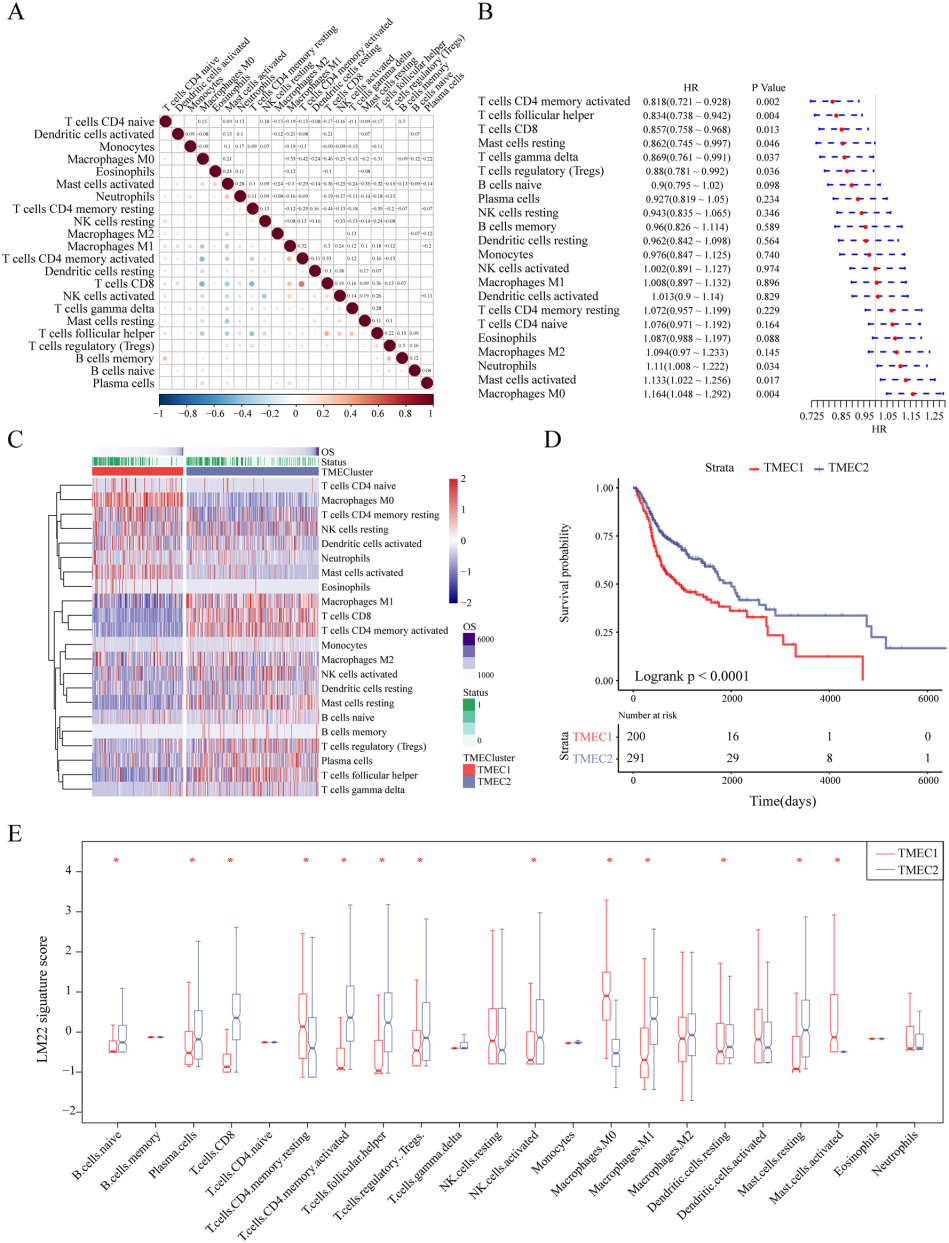
(**A**) The correlation of 22 immune cells of TME, in which the dot size and color indicate correlation, blue indicates negative correlation, red indicates positive correlation, white area in the figure indicates insignificant, and the number in the upper right corner of the figure represents correlation coefficient; (**B**) Forest map of 22 immune cells of TME; (**C**) The heat map of 22 immune cell scores. Red indicates high scores, blue indicates low scores; (**D**) KM survival curves of two types of TMEC; (**E**) Distribution box plot of 22 immune cell scores in two types of TMEC, * indicates significant difference.

We used CIBERSORT to calculate 22 immune cell scores in the training set and analyzed the correlation between them (Fig. 2A, Table S1). We used a univariate cox model to analyze the relationship between these 22 immune cell scores and prognosis. The score of Macrophage M0, Mast cell activated, and Neutrophils were significantly associated with poor prognosis (log rank *p* < 0.05, HR > 1). The score of T cells CD4 memory activated, T cells follicular helper, T cells CD8, etc. was associated with a better prognosis (log rank *p*  < 0.05, HR < 1). The detailed results are shown in Fig. 2B and Table S2. We selected 8 immune cell scores that were significantly associated with prognosis for consistent cluster analysis. As shown in Figure S1, select k = 2 as the optimal cluster number based on the CDF value and Delta area, so the two types of subtypes based on the immune cells score were defined as TMEC1 and TMEC2 (Table S3). Figure 2C shows the heat map of 22 immune cell scores in TME. From the clustering results, immune cells such as Macrophages M0, T cells CD4 memory resting, Mast cells activated, etc. have higher scores in the TMEC1 subtype, while Macrophages M1, T cells CD8, T cells CD4 memory activated, etc. have higher scores in the TMEC2 subtype. The overall survival between the two TMEC subtypes indicated a significant difference in prognosis (log rank *p* < 0.0001), as shown in Fig. 2D. We analyzed the difference in 22 immune cell scores between the two types of samples, 13 of which were significantly different (59.09%), as shown in Fig. 2E.

Similarly, we performed unsupervised clustering in the GSE65858 test set. The optimal number of clusters between k = 2–10 was evaluated and repeated 1,000 times. k = 2 was selected as the optimal cluster number based on the CDF value and Delta area (Figure S2). There was a significant difference in prognosis between two TMEC subtypes (log rank *p* = 0.015).

### Identification and clustering of DEGs between TMECs

**Figure 3:**
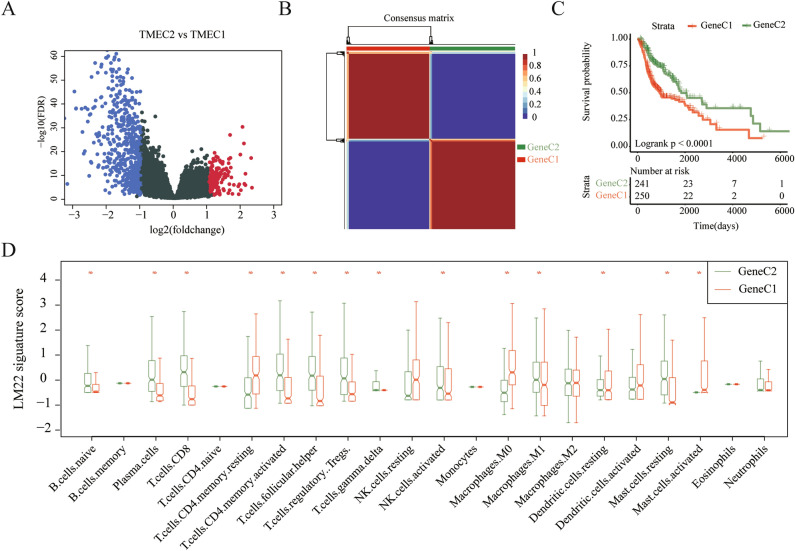
(**A**) Volcano map of DEGs between TMEC1 and TMEC2. (**B**) Consistency matrix heatmap of NMF algorithm; (**C**) KM survival curve of GeneC1 and GeneC2; (**D**) Box plot of 22 immune cell scores in GeneC1 and GeneC2.

We selected 714 DEGs between TMEC1 and TMEC2 for subsequent analysis in the training set (Fig. 3A). Among them, there were 157 up-regulated genes and 557 down-regulated genes (Table S4). We first filtered out genes that have a value of 0 in more than 50% of the samples. After that, 669 filtered genes were analyzed for univariate cox analysis, and 279 genes significantly associated with prognosis were obtained (*p* < 0.05). Finally, based on 279 gene expression profiles, we used the NMF algorithm to re-cluster the HNSC samples in TCGA. As shown in Fig. 3B, select k = 2 as the optimal cluster number according to indicators such as cophenetic, dispersion, and rss, so the two subtypes were defined as GeneC1 and GeneC2 (Figure S2). There was a significant prognostic difference in overall survival between GeneC1 and GeneC2, as shown in Fig. S3C. We analyzed the difference in 22 immune cell scores between the two types of samples. GeneC2 with high scores of T cells CD8, T cells CD4 memory activated, etc. has a better prognosis. GeneC1 with low scores of Macrophages M0, T cells CD4 memory resting、Mast cells activated, etc. has a poor prognosis. (Fig. 3D).

### Construct a prognostic risk model based on TMEscore

**Figure 4:**
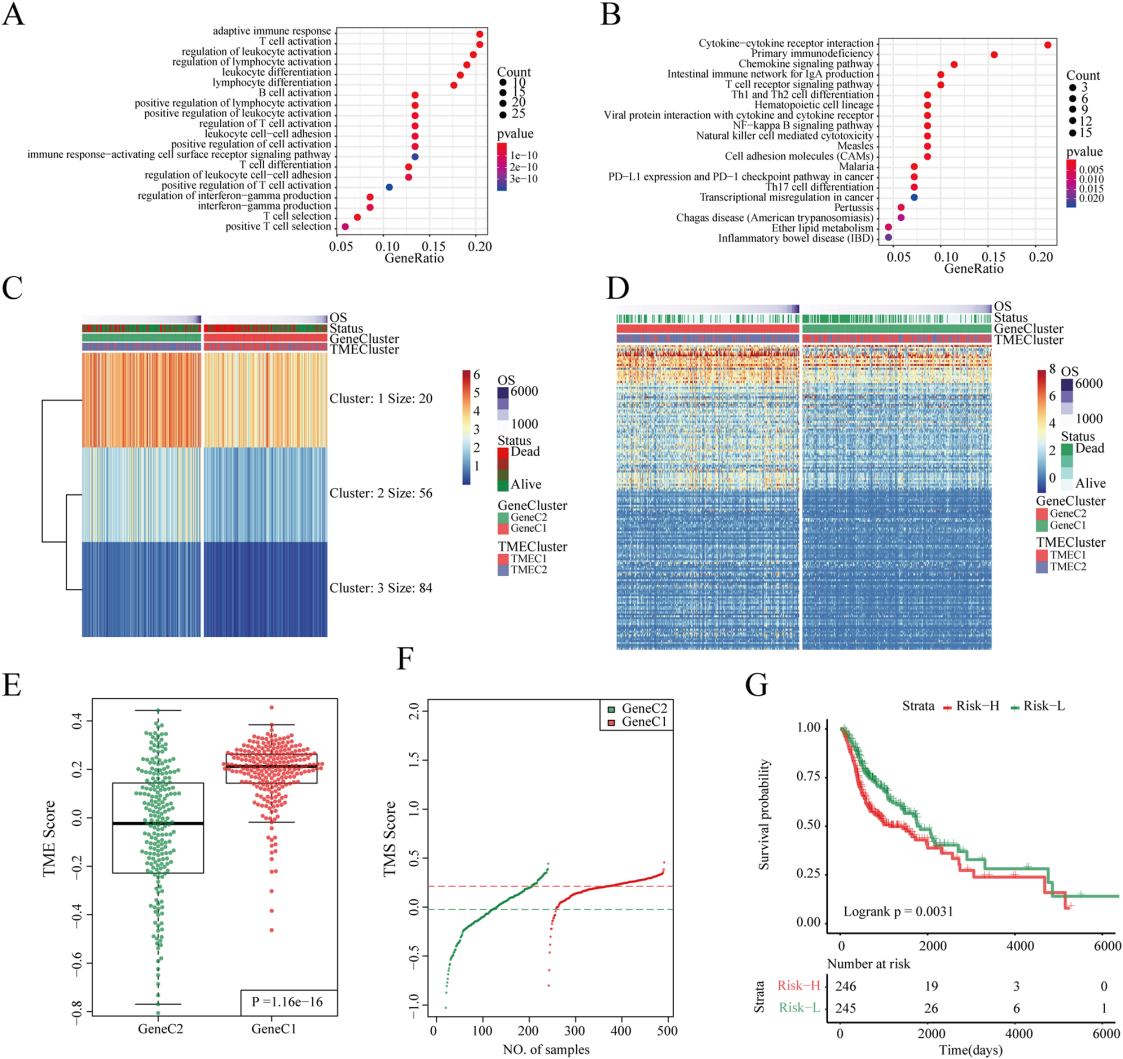
(**A**) GO enrichment analysis of 160 genes; (**B**) KEGG enrichment analysis of 160 genes; (**C**) k-means clustering results of 160 genes; (**D**) heat map of 160 gene expression levels; (**E**) Comparison of TMEscore between GeneC1 and GeneC2; (**F**) TMEscore distribution of GeneC1 and GeneC2; (**G**) KM survival curves for the Risk-H and Risk-L.

We evaluated the importance of 279 DEGs using a random forest algorithm. Set ntree = 100 according to the error rate, as shown in Figure S4A. A total of 160 candidate feature genes were identified by selecting DEGs with cumulative importance > 95% (Figure S4C). The GO and KEGG enrichment analysis showed that they mainly participated in pathways such as the adaptive immune response, T cell activation, lymphocyte differentiation, regulation of lymphocyte activation, regulation of leukocyte activation, leukocyte differentiation, B cell activation, T cell differentiation, positive regulation of lymphocyte activation, T cell selection. (Fig. 4A,B, Table S5–S6). Candidate feature genes were clustered using the k-means algorithm. The optimal number of clusters is 3 (Fig. 4C), defined as signature G1, signature G2, and signature G3, which contains 20, 56, and 84 genes, respectively. These genes have different expression patterns in each sample. Signature G3 is the low expression group, signature G1 is the high expression group, and signature G2 is in the middle (Fig. 4D).

The TMEscore of different subtypes was calculated based on the prognostic risk model, and the detailed information is shown in Tables S7–S9. By comparing the TMEscore of the two GeneCs, we found that the score of GeneC1 with the worst prognosis is significantly higher than the GeneC2 with the best prognosis (Fig. 4E, F). The median value of the TMEscore (0.146) was chosen as a threshold to classify samples into the Risk-H group and the Risk-L group. There was a significant difference in the prognosis between the Risk-H group and the Risk-L group (log rank *p* = 0.0031, HR = 1.52 (1.16–2.00) Fig. 4G).

### Relationship between TMEscore and clinical characteristics

We evaluated the relationship between TMEscore and clinical information, such as T staging, N staging, M staging, TNM stage, Age, Grade, Gender, smoking history and HPV. The results showed significant differences in TMEscore in different T staging samples. There are significant differences in TMEscore for different Graded samples. TMEscore was significantly higher in HPV negative samples than that in HPV positive samples. This significant difference was not observed in other clinical information (Figure S5).

### Relationship between TMEscore and immune genes

**Figure 5:**
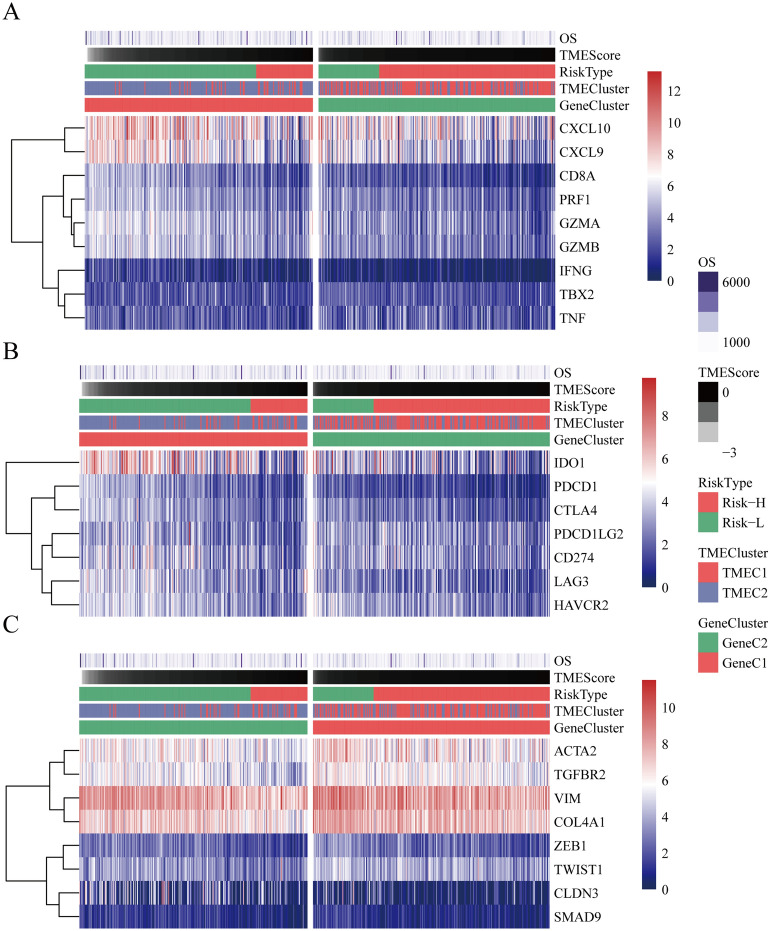
(**A**) heatmap of immune activation genes expression of TCGA samples; (**B**) heatmap of the immune checkpoint gene expression of TCGA samples; (**C**) heatmap of TGF pathway genes expression of TCGA samples.

We analyzed the relationship between immune activation genes and TMEC, GeneC, and TMEscore. These genes have different expression patterns in different subtypes (Fig. 5A). Among them, genes such as CXCL10, CXCL9, GZMA, GZMB, PRF1, IFNG, TBX2, TNF, CD8A were significantly lower in the Risk-H group with poor prognosis than those in the Risk-L group (Figure S6A). The relationship between the expression of the immune checkpoint gene and TMEC, GeneC, and TMEscore is shown in Fig. 5B. Most genes are at a low expression level. Among them, genes such as PDCD1, CTLA4, LAG3, PDCD1LG2, IDO1, CD274, HAVCR2 were significantly lower in the poor prognosis of the Risk-H group than those in the Risk-L group (Figure S6B). The expression levels of the TGF/EMT pathway activation genes on TMECs, GeneCs, and TMEscore are shown in Fig. 5C. Among them, genes such as ACTA2, TGFBR2, VIM, ZEB1, CLDN3, SMAD9 were significantly lower in the poor prognosis of the Risk-H group than those in the Risk-L group (Figure S6C), and there was no significant difference in other genes. Also, we found similar phenomena in TMEC1 and TMEC2, as shown in Figure S7.

### Relationship between TMEscore and genomic variation

**Figure 6:**
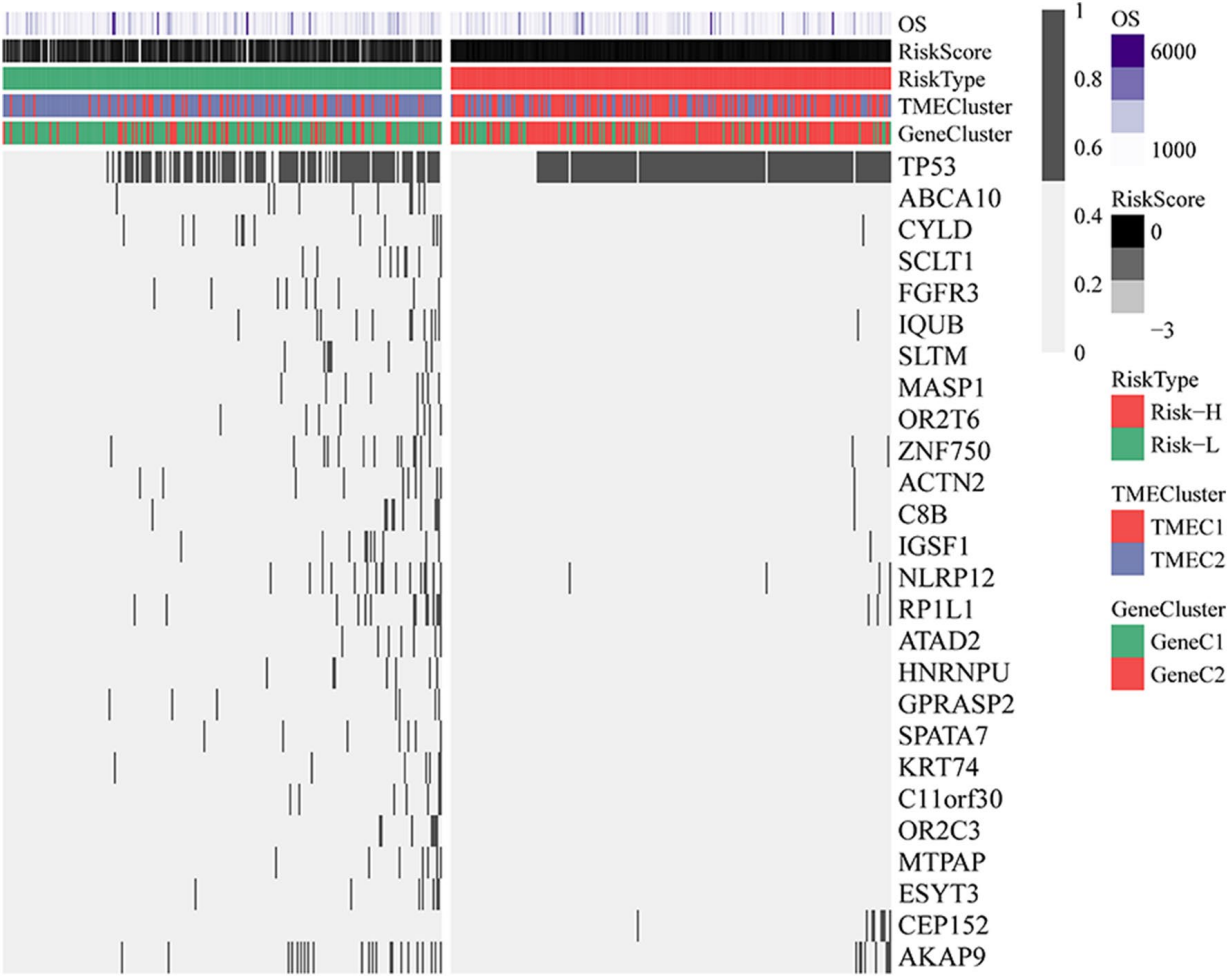
Relationship between TMEscore and genomic mutations. The horizontal axis represents the sample, the vertical axis represents the gene, the black rectangle represents the mutation, and the gray represents the unmutated.

We downloaded the predicted MAF results of the mutect2 software. Training set samples with SNP data were included in subsequent analysis. To observe the relationship between the distribution of gene mutations and TMEScore, Fisher's exact test was used to compare genes with significant differences in mutation frequency between the Risk-H and Risk-L samples. Finally, 26 genes with different mutation frequency were identified (*p* < 0.01). (Fig. 6, Table S10). Among them, the mutation frequency of TP53 and CEP152 in Risk-H was significantly higher than that of Risk-L.

### External dataset validation

We used the same method to perform TMEscore calculations in the test set. The median value of the TMEscore was chosen as a threshold to classify samples into the Risk-H group and the Risk-L group. In the test set, we also analyzed the relationship between TMEscore and prognosis, clinical data, and immune gene expression.

**Figure 7:**
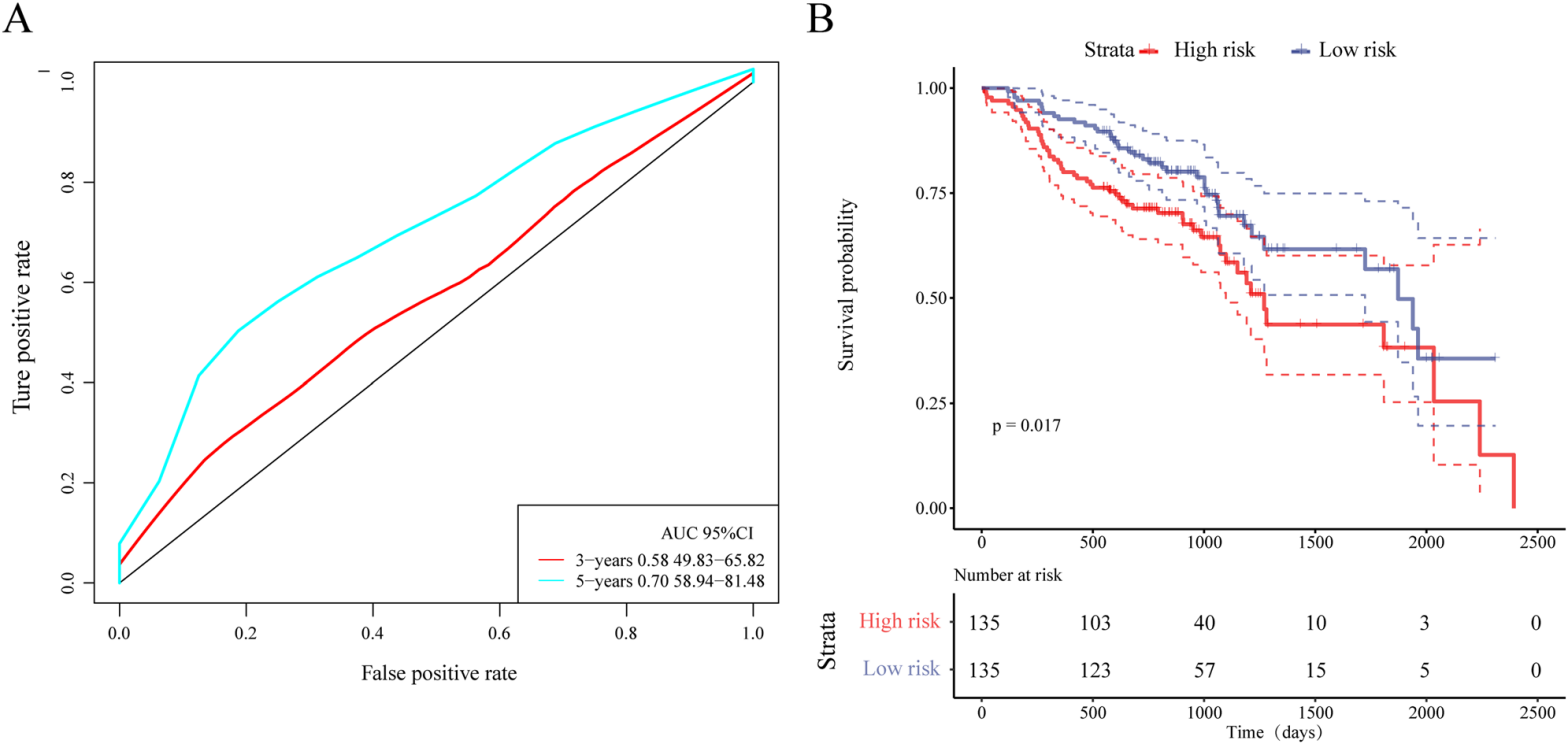
(**A**) ROC curve and AUC values in the test set; (**B**) KM survival curves for the two groups of samples in the test set.

We ploted survival curves for the Risk-H group and the Risk-L group. As a result, it was found that the AUC value of five-years was 0.70 (Fig. 7A). There was a significant difference in prognosis between the two groups (log rank *p* = 0.017), as detailed in Fig. 7B.

We evaluated the relationship between TMEscore and clinical information, such as T staging, N staging, M staging, TNM stage, Age, Gender, smoking history and HPV in the test set. TMEscore was significantly higher in HPV negative samples than that in HPV 16 and other HPV samples. This result is similar to the one in the training set. This significant difference was not observed in other clinical information (Figure S8).

In the test set, we analyzed the relationship between immune activation genes and TMEscore. Among them, genes such as CXCL10, CXCL9 were significantly lower in the Risk-H group with poor prognosis than those in the Risk-L group (Figure S9A). The relationship between the expression of the immune checkpoint gene and TMEscore is shown in Figure S9B. Among them, genes such as IDO1, HAVCR2 were significantly lower in the poor prognosis of the Risk-H group than those in the Risk-L group. The relationship between the expression levels of the TGF/EMT pathway activation genes and TMEscore is shown in Figure S9C. Among them, genes such as ACTA2, VIM were significantly lower in the poor prognosis of the Risk-H group than those in the Risk-L group. The experimental results of the test set are similar to those of the training set.

## Discussion

A comprehensive understanding of HNSC not only needs to focus on tumor cells but also on TME^33^.We have elucidated the global landscape of the interaction between HNSC clinical characteristics and infiltrating TME cells. With the help of several computational algorithms, we have established a method to quantify the infiltrating mode of TMEscore.

In this study, we obtained gene expression data and clinical annotations from 761 HNSC samples. TMEC1 and TMEC2 were obtained by clustering of 8 immune cells significantly related to prognosis. After that, DEGs associated considerably with prognosis were clustered to obtain GeneC1 and GeneC2. Among these subtypes, TMEC2 and GeneC2 have a better prognosis. It is worth noting that their B cells naive, Plasma cells, T cells CD8, T cells CD4 memory activated, T cells follicular helper, T cells regulatory Tregs, NK cells activated, Mast cells resting all scored higher. TMEC1 and GeneC1 have poor prognosis. Their T cells CD4 memory resting, Macrophages M0, Dendritic cells resting, Mast cells activated all scored higher. Of the 22 immune cells, only the scores of Macrophages M1 and T cells gamma delta indicated different results. Therefore, we believe that there are some similarities in the results of the two clustering methods. It can be speculated that the prognosis of TMEC2 and GeneC2 may be related to the enhanced immunological activity of B cells naive, plasma cells, T cells CD8, T cells CD4 memory activated, T cells follicular helper, T cells regulatory Tregs, NK cells activated, and Mast cells resting.

We further screened and clustered prognosis-related DEGs to obtain signatures G1, signature G2, and signature G3. On this basis, we have established an algorithm for a comprehensive evaluation of TMEscore. A high TMEscore is associated with poor prognosis. There was a significant difference between the Risk-H group and the Risk-L group (log rank *p* < 0.001, HR = 4.06). After that, the samples classified by TMEscore were correlated with clinical features. The results showed that there were significant differences in TMEscore for samples of different T staging, grades, and HPV. Comprehensive analysis showed that the TMEscore may be a potential predictor of prognosis in HNSC.

In previous studies, prognosis prediction based on TNM staging was the primary prognosis prediction model for HNSC and many other solid tumors^34^. With the discovery of the relationship between the characteristics of tumor immune microenvironment and tumor progression, it was confirmed that the information of prognosis prediction based on a single TMN stage is incomplete^33^. Therefore, this study attempted to establish a quantitative method of TME infiltration pattern and predicted the prognosis of cancer based on the TME characteristics.

In addition to clinical information, we also analyzed the relationship between TMEscore and immune gene expression, and genomic variation. Samples based on the TMEscore classification were associated with immune activation genes, activation genes for the TGF pathway, and immune checkpoint genes. The results showed that the expression of immune activation genes, immune checkpoint genes, and TGF pathway activating genes showed the same trend. Overall, these genes are underexpressed in Risk-H samples and highly expressed in Risk-L samples. It was found that the mutation frequency of TP53 and CEP152 in the Risk-H group was significantly higher than that in the Risk-L group, while other genes showed the opposite trend. TP53 is one of the most common tumor suppressor genes^35^. TP53 protein is mainly involved in regulating cell cycle, promoting apoptosis, and participating in DNA damage repair^36^. Mutations or deletions of TP53 will result in the cell cycle disorders and inhibition of apoptosis. More importantly, it will affect the function of DNA damage repair, leading to genomic instability^37^. CEP152 is the coding gene for the Centrosomal protein of 152 kDa^38^. The relationship between its mutation and HNSC has not been reported in the literature.

Our research also has certain limitations, however. At present, genome-wide sequencing data from more than 1,000 samples is still challenging to obtain^39^. With the restriction of social ethics and other factors, the number of available cancer samples is relatively limited. As biotechnology evolves, the number of patient samples increases, which is conducive to the improvement of data integrity and model reliability. Also, our research requires validation of biological experiments. In the next study, we will screen and validate DEGs between different subtypes of HNSC. Their molecular functions will be studied to analyze their role in the development of cancer. Correlation analysis of their regulated protein expression with clinical features of patients will be performed. The reliability of TMEscore will be verified from the overall level, providing a valid criterion for the prognosis and diagnosis of HNSC.

## Conclusions

In summary, in this study, we systematically evaluated the TME infiltration pattern from 816 HNSC patients and developed a TME infiltration model approach, which may be a potential predictor of HNSC prognosis.

## Supplementary information

Supplementary file1 (PDF 1491 kb)
