# Supplementary material for: Tumor microenvironment characterization in head and neck cancer identifies prognostic and immunotherapeutically relevant gene signatures
Source: Sci Rep. 2020 Jul 7;10:11163. doi: 10.1038/s41598-020-68074-3 (PMC7341839; doi:10.1038/s41598-020-68074-3)
Supplement: Supplementary file 1 — Supplementary file1 (PDF 1491 kb) [file 41598_2020_68074_MOESM1_ESM.pdf]

Supplementary Data for

Tumor Microenvironment Characterization in head and neck cancer Identifies Prognostic and Immunotherapeutically Relevant Gene Signatures

Mengqi Huo [3], Ying Zhang [2], Zhong Chen [1], Suxin Zhang [1], Yang Bao[1], Tianke Li [1, *]

[1] The Fourth Hospital of Hebei Medical University, Department of Stomatology, Shijiazhuang 050011, China
[2] The Third Hospital of Shijiazhuang City, Department of Stomatology, Shijiazhuang, 050011, China
[3] Beijing University of Chinese Medicine, School of Chinese Material Medica, Beijing, 102488, China

* Correspondence: 185563122@qq.com;

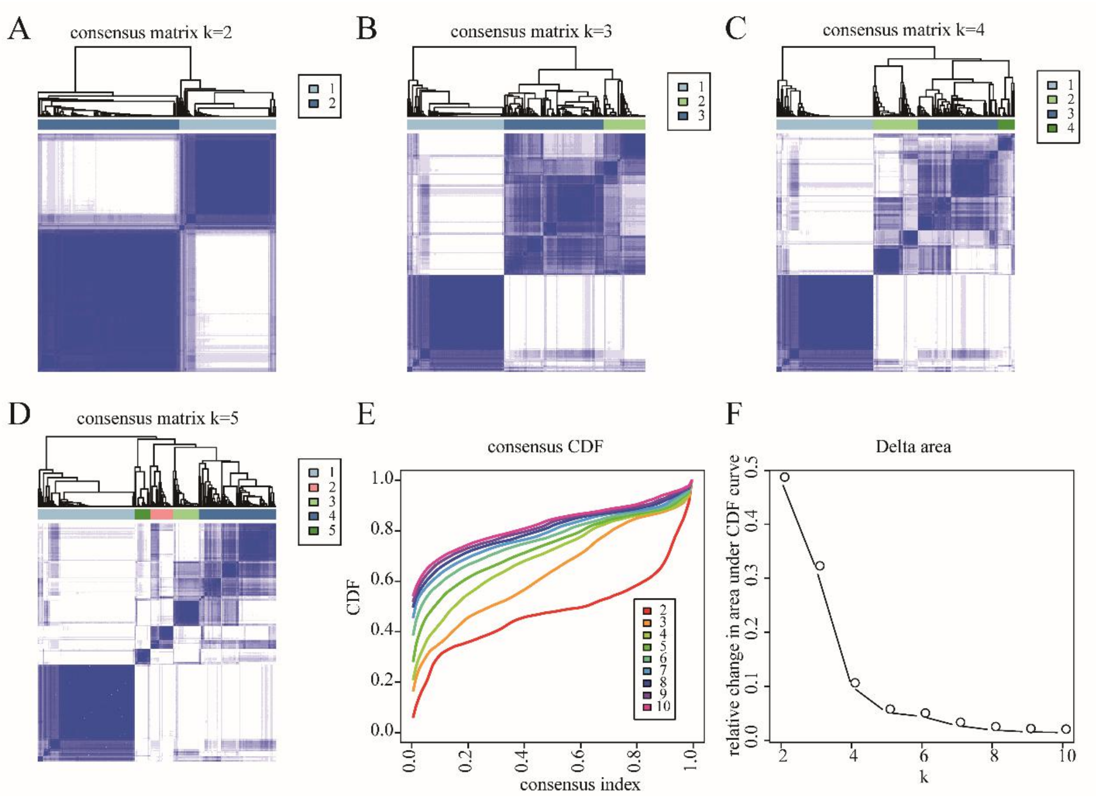

Figure S1: Consensus matrix for TME-infiltrating cells classification with the corresponding heat map in training set. (A-D) The color - coded heatmap corresponding to the consensus matrix for k = 2,3,4,5 obtained by applying consensus clustering. The color gradients were from 0 to 1, representing the degree of consensus, with white corresponding to 0 and dark blue to 1. (E) The cumulative distribution function (CDF) curves in consensus cluster analysis. CDF curves of consensus scores by different subtype number (k = 2, 3, 4, 5, 6,7,8,9,10) were represented. (F) Delta area curve of consensus clustering, indicating the relative change in area under CDF curve for each category number k compared with k – 1. The horizontal axis represents the category number k and the vertical axis represents the relative change in area under CDF curve.

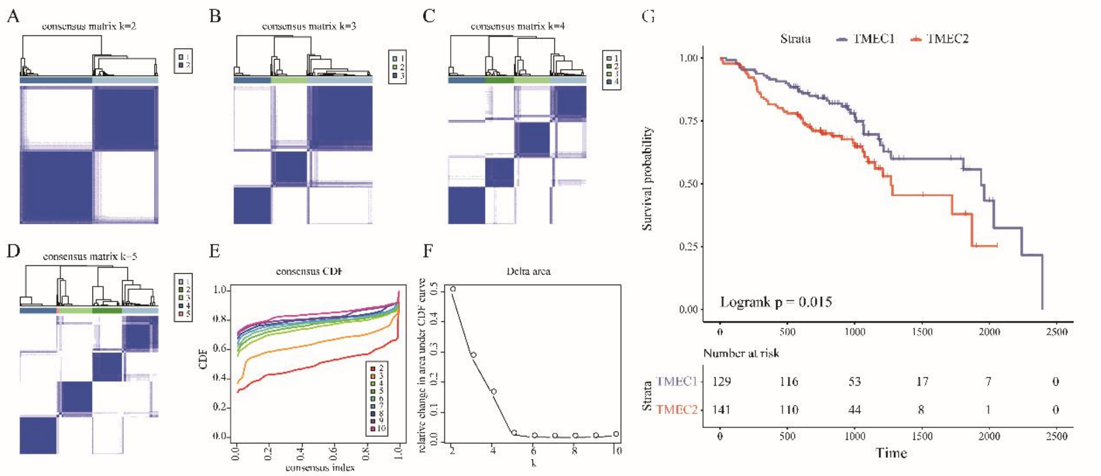

Figure S2: Consensus matrix for TME-infiltrating cells classification with the corresponding heat map in test set. (A-D) The color - coded heatmap corresponding to the consensus matrix for k = 2, 3, 4, 5 obtained by applying consensus clustering. The color gradients were from 0 to 1, representing the degree of consensus, with white corresponding to 0 and dark blue to 1. (E) CDF curves in consensus cluster analysis. CDF curves of consensus scores by different subtype number (k = 2, 3, 4, 5, 6, 7, 8, 9, 10) were represented. (F) Delta area curve of consensus clustering, indicating the relative change in area under CDF curve for each category number k compared with k – 1. The horizontal axis represents the category number k and the vertical axis represents the relative change in area under CDF curve. (G) KM survival curves of two types of TMEC.

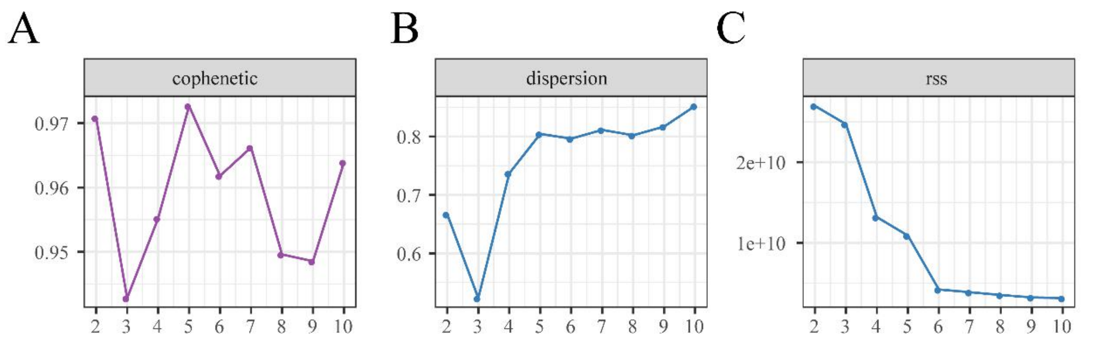

Figure S3: (A-C) distribution of cophenetic, dispersion, and rss when rank=2-10. Among them, the cophenetic correlation is derived from the consistency matrix proposed by Brunet et al., and is used to reflect the cluster obtained from NMF. The value is between 0 and 1. The larger the value, the more stable the cluster is. Rss is the residual sum of squares, which is used to reflect the clustering performance of the model. The smaller the value, the better the effect of model clustering.

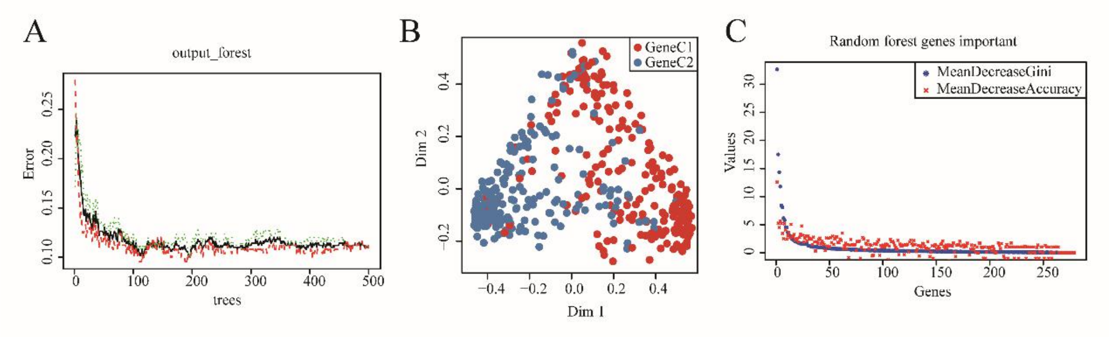

Figure S4: (A) The error rate of the random forest varies with the trees parameter; (B) Multidimensional scaling (MDS) plot for GeneCluster (GencC1 and GeneC2); (C) Random forest importance for DEGs with mean decrease gini index (blue) and mean decrease accuracy (red).

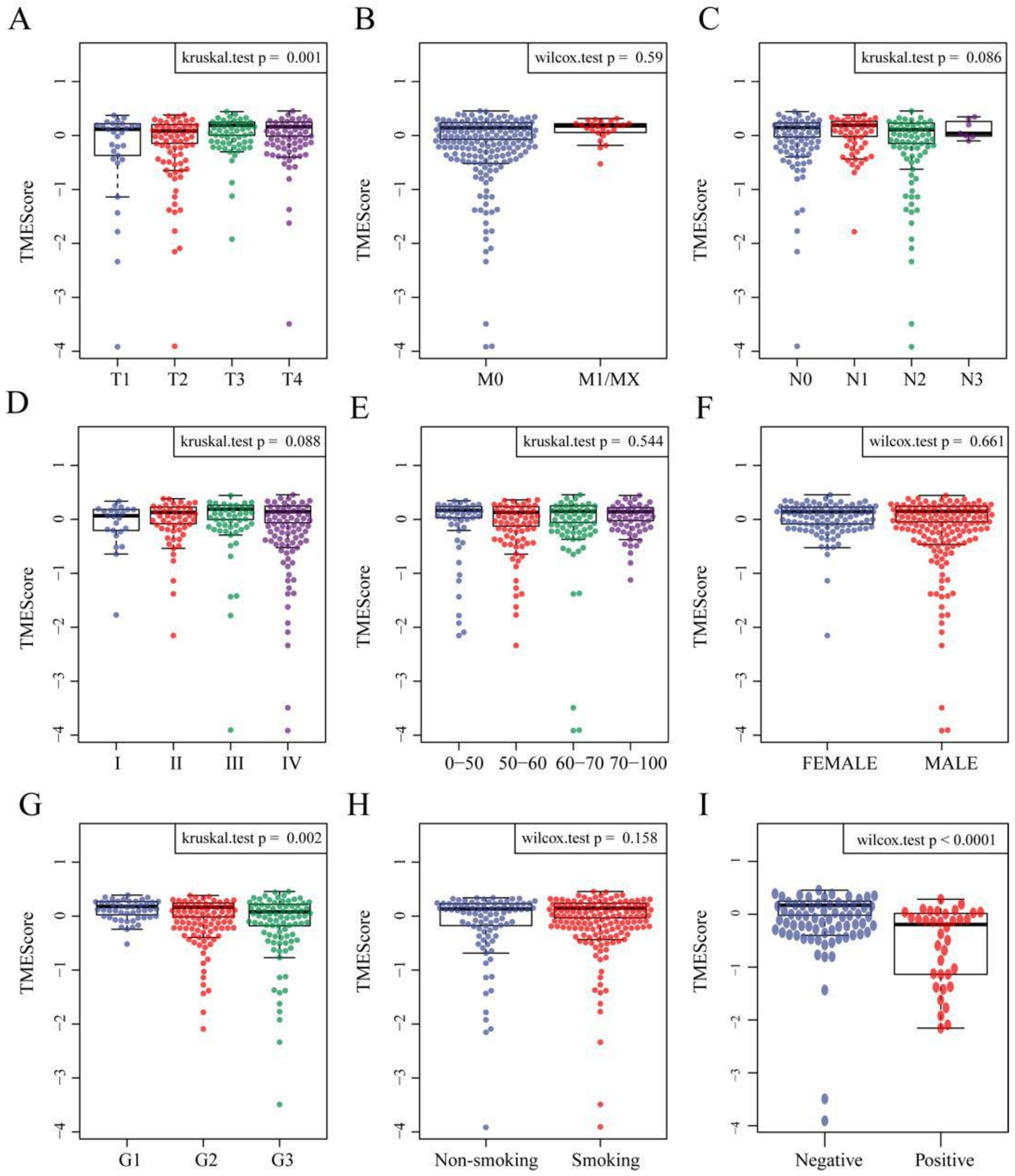

Figure S5: The relationship between TME score and clinical information in the train set. (A) The relationship between T staging and TMEscore; (B) The relationship between N staging and TMEscore; (C) The relationship between M staging and TMEscore; (D) The relationship between TNM stage staging and TMEscore; (E) The relationship between Age and TMEscore; (F) The relationship between Gender and TMEscore; (G) The relationship between Grade and TMEscore; (H) The relationship between smoking history and TMEscore; (I) The relationship between HPV and TMEscore.

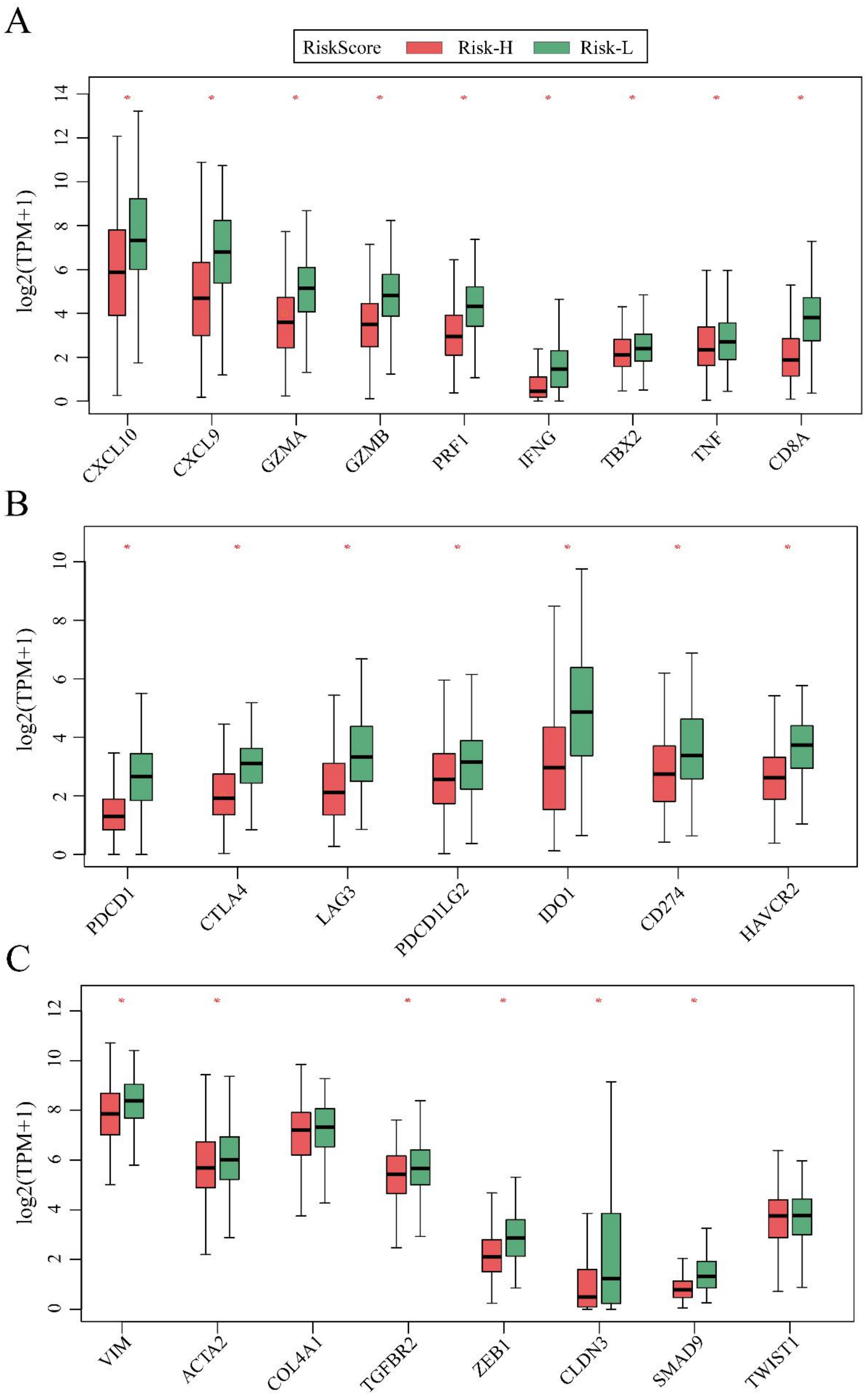

Figure S6: (A-C) The expression level of immune activation genes, immune checkpoint genes, and TGF pathway genes of the risk-H group (red) and risk-L group (green). * indicates significant difference.

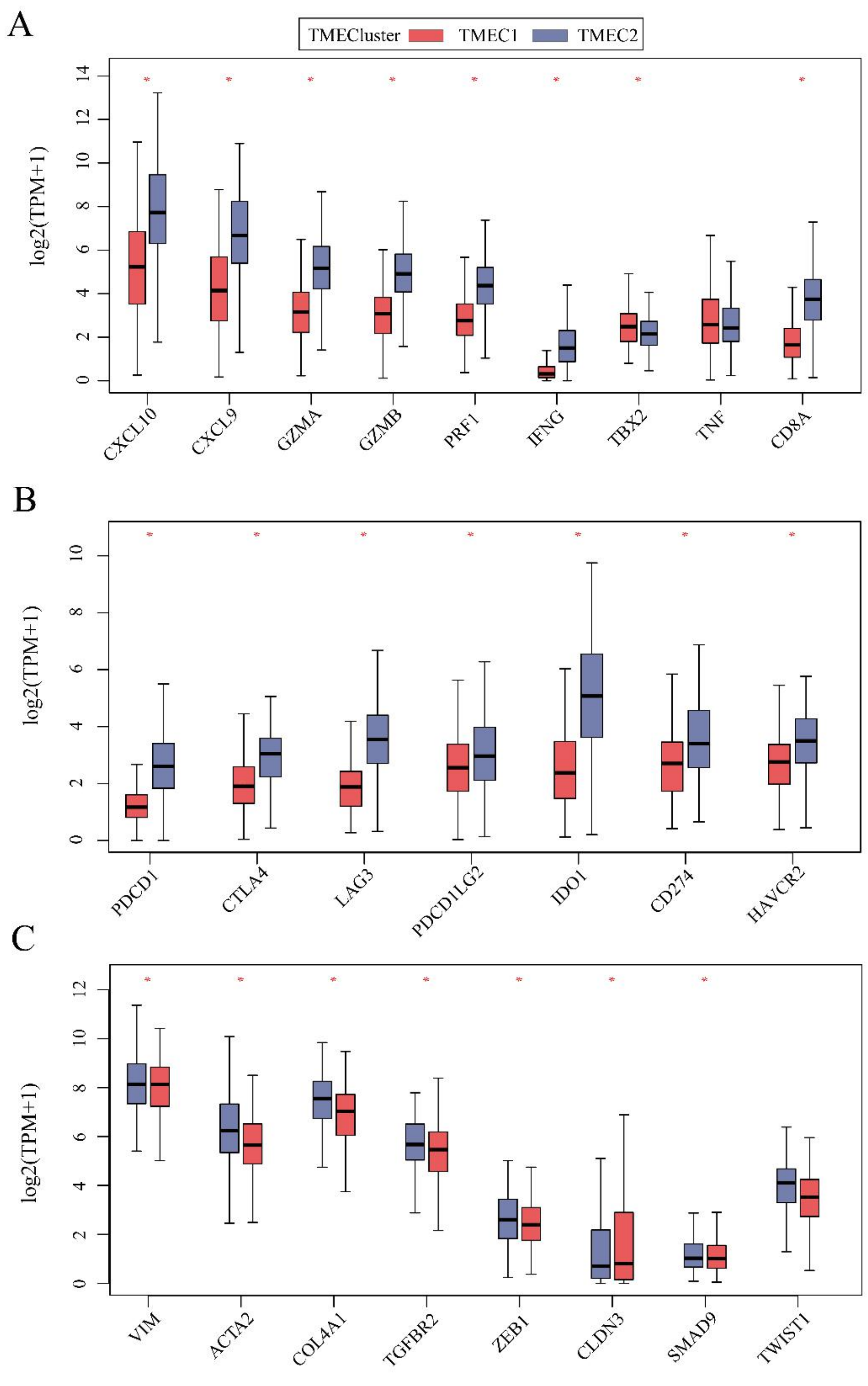

Figure S7: (A-C) The expression level of immune activation genes, immune checkpoint genes, and TGF pathway genes of the TMEC1 group (red) and TMEC2 group (green). * indicates significant difference.

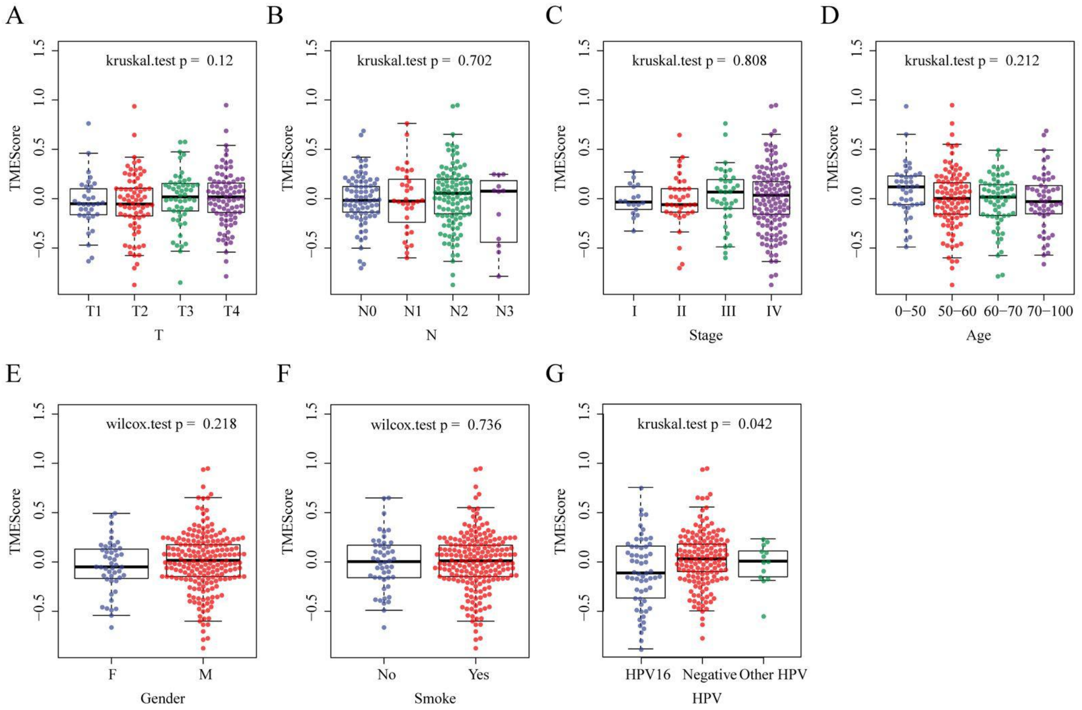

Figure S8: The relationship between TME score and clinical information in the test set. (A) The relationship between T staging and TMEscore; (B) The relationship between N staging and TMEscore; (C) The relationship between TNM stage staging and TMEscore; (D) The relationship between Age and TMEscore; (E) The relationship between Gender and TMEscore; (F) The relationship between smoking history and TMEscore; (G) The relationship between HPV and TMEscore.

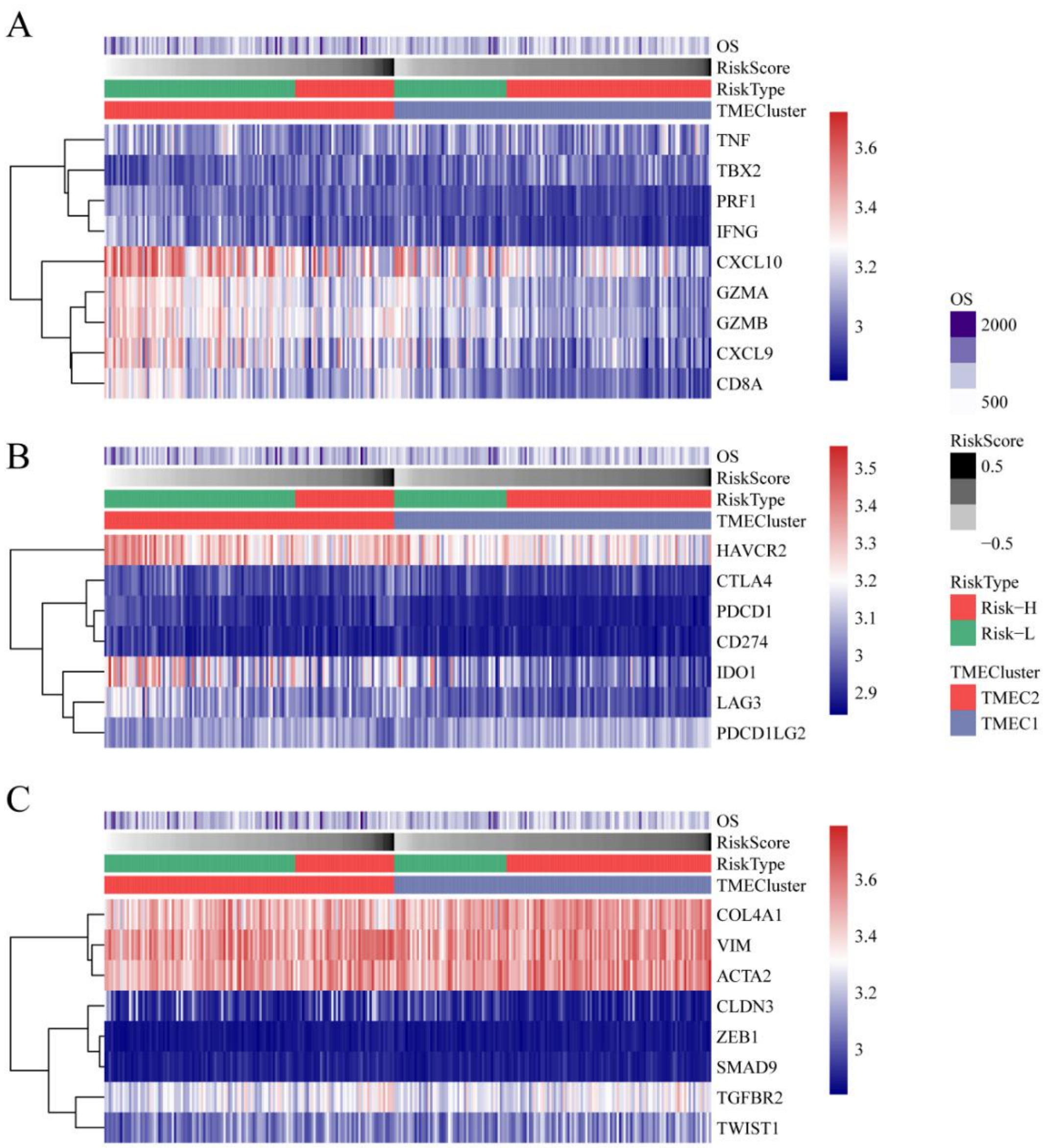

Figure S9: (A) heatmap of immune activation genes expression of GEO samples; (B) heatmap of the immune checkpoint gene expression of GEO samples; (C) heatmap of TGF pathway genes expression of GEO samples.

Table S1: 22 immune cell scores and correlations in TME

	B cells naive	B cells memory	Plasma cells	T cells CD8	T cells CD4 naive	T cells CD4 memory resting	T cells CD4 memory activated	T cells follicular helper	T cells regulatory (Tregs)	T cells gamma delta	NK cells resting	NK cells activated	Monocytes	Macrophages M0	Macrophages M1	Macrophages M2	Dendritic cells resting	Dendritic cells activated	Mast cells resting	Mast cells activated	Eosinophils	Neutrophils	P-value	Correlation	RMSE
TCGA-4P-AA8J	-0.50	-0.13	0.78	-0.43	-0.25	0.54	-0.38	-0.85	-0.14	-0.40	-0.79	0.01	-0.27	0.20	0.38	0.14	0.52	-0.77	-0.11	-0.49	-0.17	-0.43	0.00	0.63	0.79
TCGA-BA-4074	-0.34	-0.13	-0.45	-0.87	-0.25	1.60	-0.93	-1.03	-0.85	-0.40	-0.32	-0.80	-0.27	-0.19	-0.34	0.30	-0.13	0.45	-0.92	3.70	0.44	0.69	0.01	0.26	1.00
TCGA-BA-4075	-0.48	-0.13	-0.82	-0.94	2.11	0.29	-0.93	-1.03	-0.85	-0.40	0.25	-0.59	0.04	0.23	-1.37	0.68	0.57	0.29	-0.92	2.15	12.17	3.83	0.06	0.13	1.06
TCGA-BA-4076	-0.50	-0.13	-0.86	-1.00	-0.25	-0.99	-0.56	0.70	0.37	-0.40	-0.37	-0.80	-0.27	2.16	-1.33	-1.71	0.37	1.59	-0.92	1.63	-0.17	-0.37	0.01	0.25	1.05
TCGA-BA-4077	0.01	-0.13	-0.77	0.16	-0.25	-0.09	-0.56	0.09	-0.67	-0.40	-0.79	1.44	-0.27	-0.66	2.02	-0.01	0.41	0.06	1.78	-0.49	-0.17	-0.34	0.00	0.51	0.86
TCGA-BA-4078	0.47	-0.13	-0.75	-0.66	-0.25	2.03	-0.60	0.42	1.06	-0.40	0.64	-0.80	0.74	-1.37	-0.70	1.24	2.74	-0.77	-0.92	0.12	-0.17	-0.43	0.01	0.25	0.98
TCGA-BA-5151	-0.26	-0.13	-0.14	-1.00	-0.25	2.40	-0.50	-1.03	-0.85	-0.40	1.78	-0.80	-0.27	-0.57	-0.61	-0.10	-0.62	1.03	-0.92	0.86	-0.17	5.23	0.01	0.28	0.97
TCGA-BA-5152	0.34	-0.13	-0.22	0.62	-0.25	0.07	1.26	-0.57	0.22	-0.40	-0.15	-0.69	-0.27	-0.73	0.08	0.09	0.08	1.52	-0.09	-0.49	-0.17	0.29	0.01	0.26	0.98
TCGA-BA-5153	-0.02	-0.13	0.27	1.67	-0.25	-0.41	2.31	0.05	1.68	-0.40	4.45	-0.80	0.07	-0.92	-1.11	-0.83	-0.45	-0.52	-0.34	-0.49	-0.17	-0.43	0.00	0.34	0.95
TCGA-BA-5555	-0.34	-0.13	0.52	-0.27	-0.25	-0.92	0.17	0.71	0.76	-0.40	-0.79	1.27	-0.27	-0.24	0.46	-0.04	-0.19	-0.66	2.60	-0.49	-0.17	-0.15	0.00	0.29	0.97
TCGA-BA-5556	0.27	-0.13	-0.29	2.35	-0.25	-1.13	2.52	-0.83	-0.54	3.04	0.98	-0.80	-0.27	-1.23	0.34	0.62	-0.15	0.19	-0.69	-0.49	-0.17	-0.43	0.00	0.33	0.95
TCGA-BA-5557	-0.23	-0.13	-0.86	-0.53	-0.25	-0.51	-0.63	-1.03	-0.19	-0.40	0.64	-0.80	-0.27	1.96	-1.40	-1.28	-0.79	0.58	-0.92	2.26	-0.17	2.66	0.00	0.71	0.71
TCGA-BA-5558	-0.21	-0.13	-0.80	0.27	-0.25	-0.62	1.50	-0.97	-0.39	-0.40	2.00	-0.80	-0.27	0.15	0.09	1.17	-0.78	-0.57	0.39	0.13	-0.17	0.51	0.00	0.38	0.93
TCGA-BA-5559	0.36	-0.13	-0.34	2.32	-0.25	-1.13	2.01	0.17	2.21	-0.40	0.41	-0.80	-0.27	-0.87	0.07	-0.30	-0.50	-0.77	0.72	-0.49	-0.17	-0.43	0.00	0.40	0.92
TCGA-BA-6868	-0.50	-0.13	-0.62	-0.85	4.86	0.92	-0.93	-1.03	-0.85	-0.40	1.32	-0.80	-0.27	1.48	-0.46	-1.71	-0.79	1.19	-0.92	0.31	1.92	-0.43	0.00	0.63	0.78
TCGA-BA-6869	-0.50	-0.13	1.75	0.67	-0.25	-1.13	3.02	1.60	-0.85	-0.40	-0.79	0.51	-0.27	-0.92	-0.23	-0.91	-0.70	-0.18	-0.92	1.51	1.85	1.10	0.06	0.13	1.03
TCGA-BA-6870	-0.31	0.90	3.91	-0.62	0.98	-0.32	-0.40	-0.80	0.29	-0.40	-0.79	0.00	-0.27	-0.45	-1.43	-1.16	0.13	1.01	-0.19	0.42	-0.17	0.15	0.00	0.36	0.93
TCGA-BA-6871	-0.47	-0.13	-0.42	0.49	-0.25	1.74	0.88	-0.07	-0.74	-0.40	3.66	-0.40	1.08	-0.54	-0.08	-1.12	-0.71	-0.55	-0.92	0.40	-0.17	0.46	0.00	0.32	0.96
TCGA-BA-68	-0.24	-0.13	-0.82	-0.23	-0.16	0.04	-0.51	-1.03	-0.16	-0.40	-0.45	-0.80	-0.27	1.15	0.29	0.36	-0.71	0.10	-0.92	0.84	-0.17	0.03	0.00	0.58	0.82

72																									
TCGA-BA-6873	-0.50	-0.13	-0.55	0.31	-0.25	1.59	0.22	-1.03	-0.85	-0.40	0.75	1.13	-0.27	-0.27	0.44	-0.03	-0.11	-0.66	-0.92	0.12	-0.17	-0.43	0.00	0.47	0.88
TCGA-BA-7269	-0.50	-0.13	1.00	-0.15	-0.25	0.51	-0.93	0.26	0.35	-0.40	-0.79	1.89	-0.05	-0.07	-0.92	0.54	-0.62	0.31	-0.92	0.19	-0.17	-0.35	0.00	0.48	0.88
TCGA-BA-A4IF	1.70	-0.13	-0.10	-0.90	-0.25	0.68	-0.93	-0.41	-0.77	-0.40	0.30	1.39	-0.02	0.97	-0.78	-0.04	-0.25	-0.06	-0.92	-0.49	-0.17	-0.38	0.04	0.16	1.05
TCGA-BA-A4IG	4.05	-0.13	0.52	0.71	-0.25	-0.37	1.21	-0.52	-0.11	2.03	0.24	-0.80	-0.27	-0.97	0.32	-0.22	-0.01	-0.13	-0.76	-0.49	-0.17	-0.43	0.00	0.31	0.96
TCGA-BA-A4IH	2.10	-0.13	0.35	0.66	-0.25	-0.46	0.96	1.99	-0.63	-0.40	-0.35	-0.80	-0.27	-0.19	0.82	-1.28	-0.79	0.74	-0.92	-0.43	-0.17	-0.43	0.04	0.16	1.03
TCGA-BA-A4II	-0.50	-0.13	0.96	-0.23	-0.25	-0.28	1.40	0.59	-0.81	0.03	0.17	-0.80	-0.27	-0.20	-0.72	-0.29	-0.57	1.90	-0.92	0.48	-0.17	1.12	0.01	0.24	0.98
TCGA-BA-A6D8	-0.37	-0.13	-0.14	-0.77	-0.25	-1.02	-0.90	0.73	-0.36	0.64	-0.79	-0.55	12.87	0.47	-1.43	1.03	-0.52	1.46	-0.92	1.00	-0.17	-0.43	0.00	0.63	0.78
TCGA-BA-A6DA	-0.50	-0.13	0.71	0.89	-0.13	-0.19	0.39	-1.03	-0.85	-0.40	-0.61	0.21	-0.27	0.36	-0.54	0.02	-0.73	-0.77	-0.92	0.26	-0.17	2.03	0.05	0.14	1.04
TCGA-BA-A6DB	-0.50	-0.13	-0.47	1.06	-0.25	-0.81	-0.87	1.04	0.30	6.18	-0.79	1.01	-0.27	-0.64	0.20	-0.66	0.88	-0.21	1.40	-0.49	-0.17	-0.43	0.00	0.53	0.85
TCGA-BA-A6DD	-0.50	-0.13	1.35	1.25	-0.25	0.08	-0.10	-0.27	-0.85	-0.40	0.23	-0.80	-0.27	-0.93	1.39	-0.19	-0.37	0.31	-0.69	-0.49	-0.17	-0.34	0.00	0.52	0.86
TCGA-BA-A6DE	-0.45	-0.13	0.34	0.07	-0.25	-0.64	0.63	0.07	-0.85	-0.40	-0.79	2.40	-0.27	-0.13	0.96	-0.35	-0.30	-0.16	0.41	-0.49	8.29	-0.30	0.06	0.13	1.05
TCGA-BA-A6DG	-0.50	-0.13	-0.86	-1.00	0.57	1.20	-0.83	-1.03	-0.85	-0.40	0.36	-0.80	-0.27	1.86	-0.73	-0.01	-0.79	-0.77	-0.92	-0.12	-0.17	2.41	0.00	0.53	0.85
TCGA-BA-A6DI	-0.50	-0.13	1.85	-0.66	-0.25	0.94	-0.43	-0.51	-0.85	-0.40	-0.78	-0.43	-0.27	0.16	0.47	-0.89	-0.24	0.85	-0.84	-0.49	-0.17	-0.35	0.03	0.18	1.02
TCGA-BA-A6DJ	-0.50	-0.13	-0.68	0.72	-0.25	-0.91	1.50	-0.50	-0.79	0.86	0.88	-0.05	-0.27	0.09	0.02	-0.35	-0.79	0.68	-0.92	2.18	-0.17	-0.42	0.01	0.27	0.99
TCGA-BA-A6DL	-0.02	-0.13	1.33	0.29	4.07	-1.13	0.49	0.13	-0.76	-0.40	0.37	-0.80	-0.27	-0.68	-0.98	-0.36	-0.66	0.71	-0.92	2.85	-0.17	1.69	0.08	0.12	1.03
TCGA-BA-A8YP	-0.29	-0.13	-0.08	-0.99	-0.25	0.09	-0.93	-1.03	-0.56	-0.40	0.23	-0.80	-0.27	0.62	-0.40	1.99	-0.61	1.28	-0.92	0.76	-0.17	-0.43	0.02	0.20	1.04
TCGA-BB-4217	3.35	18.80	0.59	-0.14	5.41	0.39	-0.52	0.49	2.66	-0.40	-0.79	-0.79	0.11	-1.06	-1.22	-1.34	-0.79	-0.20	-0.40	-0.49	-0.17	-0.43	0.00	0.37	0.93
TCGA-BB-4223	1.48	-0.13	0.18	2.57	-0.25	-1.13	1.60	0.48	1.29	-0.40	0.90	-0.80	0.05	-0.72	-0.41	-1.08	-0.10	-0.77	-0.51	-0.49	-0.17	-0.43	0.00	0.43	0.90
TCGA-BB-4224	-0.50	-0.13	-0.86	-0.82	-0.25	-0.08	-0.80	-1.03	-0.36	-0.40	-0.79	-0.21	-0.27	0.51	0.19	-1.36	4.63	-0.77	0.47	-0.49	-0.17	0.28	0.01	0.26	1.01
TCGA-BB-4225	0.17	-0.13	0.46	1.74	-0.25	-1.13	1.86	1.65	0.27	-0.40	-0.79	-0.26	-0.27	-0.37	-0.24	-1.17	-0.10	-0.52	0.49	-0.49	-0.17	-0.43	0.00	0.32	0.95
TCGA-BB-42	-0.50	-0.13	-0.46	-1.00	-0.25	-0.03	-0.93	-1.03	-0.55	-0.40	2.38	-0.80	-0.27	-0.65	-1.43	3.00	0.01	3.38	-0.92	1.93	1.46	-0.21	0.06	0.14	1.05

27																									
TCGA-BB-4228	0.06	-0.13	0.29	0.35	-0.25	-1.13	-0.36	2.53	-0.18	-0.40	-0.79	-0.06	-0.27	0.60	-0.21	-0.88	0.44	-0.77	0.71	-0.49	-0.17	-0.43	0.00	0.34	0.95
TCGA-BB-8596	0.18	-0.13	-0.84	-1.00	-0.02	-1.00	-0.90	-0.23	0.17	-0.40	-0.78	-0.34	-0.27	2.29	-0.64	-0.13	-0.01	-0.64	1.33	-0.49	-0.17	-0.43	0.00	0.74	0.68
TCGA-BB-8601	3.90	-0.13	3.58	-1.00	-0.25	0.54	0.68	-1.03	-0.21	-0.40	0.79	-0.80	0.03	-0.93	-1.43	-1.71	0.41	-0.32	-0.23	0.89	-0.17	-0.43	0.22	0.07	1.04
TCGA-BB-A5HU	-0.50	-0.13	-0.71	-0.75	-0.25	1.07	-0.93	-1.03	-0.46	-0.40	0.35	-0.80	0.02	0.46	-1.20	-1.71	0.54	0.15	-0.92	5.77	-0.17	-0.43	0.02	0.20	1.05
TCGA-BB-A5HY	-0.50	-0.13	-0.70	-0.62	0.44	0.85	-0.67	-1.03	-0.85	-0.40	-0.10	-0.49	-0.27	0.28	-1.14	-0.98	-0.79	2.35	-0.92	4.97	2.81	-0.35	0.18	0.08	1.11
TCGA-BB-A5HZ	-0.19	-0.13	0.81	-0.63	-0.25	-0.45	-0.93	3.38	-0.85	-0.40	0.44	-0.80	0.33	-0.61	-0.34	0.04	0.54	0.99	-0.92	1.30	-0.17	1.17	0.12	0.10	1.04
TCGA-BB-A6UM	-0.40	-0.13	-0.55	2.48	-0.25	-1.13	1.24	2.50	1.18	-0.40	-0.04	0.82	0.06	-1.35	1.02	-0.69	-0.38	-0.08	0.14	-0.49	-0.17	-0.43	0.00	0.30	0.98
TCGA-BB-A6UO	-0.44	-0.13	-0.81	-0.26	-0.25	0.52	-0.93	-1.03	-0.85	-0.40	-0.79	2.55	-0.27	0.88	-0.08	0.47	-0.79	-0.22	-0.92	0.95	0.05	-0.13	0.00	0.48	0.88
TCGA-C9-A47Z	0.09	-0.13	-0.60	-1.00	-0.25	0.13	0.36	-0.93	-0.84	-0.40	-0.79	0.77	-0.27	1.37	0.54	0.01	-0.79	0.01	-0.92	0.18	1.31	-0.43	0.06	0.13	1.09
TCGA-C9-A480	-0.50	-0.13	-0.86	1.02	-0.25	-1.13	-0.47	1.50	-0.85	-0.40	-0.79	2.28	0.00	0.09	-1.03	-1.50	1.49	3.53	-0.74	-0.49	-0.17	-0.43	0.01	0.27	0.99
TCGA-CN-4722	-0.50	-0.13	2.68	0.68	-0.25	-0.28	-0.93	0.94	0.89	-0.40	-0.79	-0.31	0.94	-0.93	-1.08	0.26	0.25	-0.47	0.31	-0.49	-0.17	0.05	0.11	0.10	1.03
TCGA-CN-4723	-0.50	-0.13	0.64	0.15	-0.25	0.73	-0.16	-0.30	-0.85	-0.40	0.75	-0.66	0.05	-0.10	0.30	0.20	-0.70	-0.38	0.57	-0.49	-0.17	-0.43	0.00	0.55	0.84
TCGA-CN-4725	-0.33	-0.13	-0.74	-0.69	0.22	0.14	-0.93	-1.03	-0.07	-0.40	1.81	-0.80	0.64	0.42	-0.85	0.28	0.20	1.64	-0.92	1.46	-0.17	0.26	0.09	0.12	1.07
TCGA-CN-4726	-0.47	-0.13	-0.86	-1.00	-0.25	1.54	-0.93	-1.03	-0.85	-0.40	0.73	-0.80	-0.27	-0.39	-1.16	-0.32	1.44	0.06	-0.92	1.19	3.53	9.36	0.01	0.22	1.01
TCGA-CN-4727	-0.50	-0.13	-0.19	-1.00	5.86	0.23	-0.40	-1.03	0.21	-0.40	3.25	-0.80	-0.27	-0.46	-1.39	-1.18	-0.79	1.40	-0.92	1.56	-0.17	9.24	0.38	0.04	1.09
TCGA-CN-4728	-0.50	-0.13	-0.79	-0.61	-0.25	-0.35	-0.48	-0.24	0.59	-0.37	-0.68	-0.80	-0.27	1.90	-0.74	-0.21	-0.69	0.96	0.17	-0.49	-0.17	-0.43	0.00	0.77	0.66
TCGA-CN-4729	0.04	-0.13	0.17	-0.55	-0.25	1.49	0.69	-1.03	-0.69	-0.40	1.39	-0.80	0.20	-0.19	-0.92	-1.06	0.44	1.38	-0.38	0.05	-0.17	0.61	0.03	0.18	1.01
TCGA-CN-4730	-0.26	-0.13	0.53	-1.00	-0.25	-0.96	-0.93	2.01	-0.65	0.35	-0.42	-0.80	-0.27	1.69	-0.04	0.14	-0.54	-0.73	-0.92	-0.19	0.62	-0.27	0.03	0.17	1.08
TCGA-CN-4731	-0.50	-0.13	0.17	-0.27	-0.25	-0.34	-0.93	0.04	0.70	-0.40	-0.62	0.54	-0.27	1.18	-1.07	-0.75	0.15	0.94	-0.31	-0.49	-0.17	-0.43	0.00	0.36	0.95
TCGA-CN-4733	-0.50	-0.13	0.94	0.85	-0.25	0.59	0.37	0.95	0.48	0.32	-0.47	-0.80	-0.27	-0.85	-0.09	-0.36	-0.34	-0.07	0.52	-0.49	-0.17	-0.32	0.00	0.43	0.90
TCGA-CN-47	-0.48	-0.13	-0.86	2.13	-0.25	-0.54	1.17	-0.31	-0.74	0.46	1.45	-0.80	-0.27	-0.45	1.12	-0.44	-0.48	0.44	-0.58	-0.49	-0.17	-0.35	0.00	0.47	0.89

34																									
TCGA-CN-47 35	-0.50	-0.13	-0.45	-0.57	-0.25	0.42	-0.31	-0.11	-0.69	-0.40	-0.48	0.07	-0.27	1.35	-0.29	-0.63	0.03	-0.77	0.87	-0.49	-0.17	-0.43	0.00	0.44	0.91
TCGA-CN-47 36	-0.42	-0.13	-0.86	0.41	-0.25	-1.13	-0.56	1.29	0.08	-0.40	0.44	0.42	-0.27	0.49	1.16	-0.40	-0.53	-0.39	1.61	-0.49	-0.17	-0.43	0.00	0.56	0.83
TCGA-CN-47 37	-0.50	-0.13	-0.80	-1.00	-0.25	-0.54	-0.93	0.28	-0.62	-0.40	-0.79	-0.12	-0.27	2.85	-1.08	-0.39	-0.46	-0.32	0.12	-0.49	-0.17	-0.43	0.00	0.50	0.90
TCGA-CN-47 38	0.21	-0.13	-0.22	0.00	-0.25	1.08	1.11	0.08	0.33	-0.40	1.10	-0.80	0.40	-0.63	0.46	-0.66	-0.11	-0.77	0.74	-0.49	-0.17	0.77	0.00	0.32	0.95
TCGA-CN-47 39	0.07	-0.13	1.18	1.70	-0.25	0.06	0.62	0.08	0.44	-0.40	1.36	-0.80	0.38	-1.11	-0.72	-0.75	-0.40	-0.52	-0.82	-0.26	-0.17	3.39	0.01	0.23	0.99
TCGA-CN-47 40	-0.49	-0.13	-0.62	-0.09	-0.25	-0.77	-0.54	0.02	-0.57	0.64	-0.79	-0.68	-0.27	1.49	0.11	0.37	-0.39	-0.77	0.65	-0.49	-0.17	0.03	0.00	0.67	0.74
TCGA-CN-47 41	-0.50	-0.13	0.43	-0.50	-0.25	-0.87	-0.03	2.72	-0.02	-0.06	-0.79	0.38	-0.27	0.05	-0.33	0.17	1.01	-0.77	0.85	-0.49	-0.17	-0.19	0.06	0.13	1.03
TCGA-CN-47 42	0.12	-0.13	-0.03	-0.98	-0.25	1.13	-0.16	-1.03	-0.77	-0.40	0.95	-0.80	-0.27	1.37	-0.69	-0.33	-0.68	-0.20	-0.26	-0.49	-0.17	-0.25	0.00	0.70	0.73
TCGA-CN-53 55	0.17	-0.13	2.06	-0.81	0.95	0.48	-0.93	-1.03	-0.47	-0.40	1.41	-0.80	-0.27	0.56	-0.79	-0.50	-0.76	-0.48	-0.92	0.61	-0.17	0.15	0.00	0.36	0.94
TCGA-CN-53 56	-0.50	-0.13	2.05	0.31	-0.25	1.26	-0.89	-0.27	-0.76	-0.40	0.03	-0.80	1.44	-0.68	-0.84	0.78	-0.79	-0.09	-0.40	-0.29	-0.17	0.43	0.04	0.16	1.01
TCGA-CN-53 58	-0.38	-0.13	0.43	0.19	-0.25	0.73	0.36	0.24	0.28	0.28	-0.79	-0.80	0.66	-0.32	-0.69	0.59	-0.32	-0.06	0.25	-0.49	-0.17	0.26	0.00	0.59	0.83
TCGA-CN-53 59	0.57	-0.13	-0.86	-0.14	-0.25	1.73	0.15	-0.86	-0.04	-0.40	1.96	-0.80	-0.27	0.14	-0.53	-0.17	-0.79	-0.02	-0.92	-0.17	-0.17	2.07	0.00	0.50	0.87
TCGA-CN-53 60	0.07	-0.13	-0.01	0.40	-0.25	0.51	2.70	-1.03	-0.06	-0.40	1.42	-0.80	-0.27	-0.64	-0.05	0.21	-0.39	-0.51	0.43	-0.49	-0.17	-0.22	0.00	0.48	0.88
TCGA-CN-53 61	-0.48	-0.13	-0.82	-0.81	-0.25	-0.47	-0.52	-0.18	0.34	-0.40	0.54	1.15	-0.27	0.14	1.20	0.89	0.56	-0.77	1.12	-0.49	-0.17	-0.43	0.00	0.41	0.92
TCGA-CN-53 63	-0.48	-0.13	-0.15	0.36	-0.25	1.01	0.08	-1.03	-0.85	-0.40	1.23	0.22	-0.27	-0.80	1.78	0.81	-0.63	-0.47	-0.05	-0.49	-0.17	-0.43	0.00	0.44	0.90
TCGA-CN-53 64	-0.50	0.14	-0.86	-0.76	-0.25	0.24	-0.93	0.00	-0.01	-0.40	-0.79	1.50	-0.27	0.51	0.31	0.04	1.36	-0.77	0.76	-0.49	-0.17	-0.43	0.00	0.60	0.81
TCGA-CN-53 65	-0.22	-0.13	-0.86	0.81	-0.25	-1.13	0.43	0.86	-0.19	-0.40	-0.79	1.90	-0.27	-0.58	0.22	1.64	1.25	-0.77	-0.43	-0.49	-0.17	0.07	0.00	0.31	0.96
TCGA-CN-53 66	0.35	-0.13	-0.71	0.92	-0.25	0.55	0.18	-1.03	-0.85	-0.40	1.92	-0.02	-0.27	-0.26	0.52	0.38	-0.28	-0.77	-0.01	-0.49	-0.17	-0.43	0.00	0.52	0.86
TCGA-CN-53 67	-0.46	-0.13	-0.86	-0.86	-0.25	1.25	-0.90	-1.03	-0.81	-0.40	0.08	-0.80	-0.27	0.12	0.25	-0.55	4.25	-0.77	-0.88	-0.49	-0.17	-0.43	0.00	0.50	0.87
TCGA-CN-53 69	-0.22	-0.13	-0.03	0.41	-0.25	-0.20	0.13	-0.69	-0.85	-0.40	0.78	-0.06	-0.27	-0.31	3.11	-0.01	-0.64	-0.77	-0.92	-0.49	-0.17	0.45	0.00	0.57	0.82
TCGA-CN-53	-0.23	-0.13	-0.86	-0.42	-0.25	0.54	0.27	-1.03	-0.77	-0.40	-0.34	-0.61	-0.27	0.02	0.88	2.92	-0.42	-0.77	-0.92	-0.20	-0.17	0.43	0.00	0.39	0.93

70																									
TCGA-CN-5373	0.20	-0.13	-0.79	-0.99	-0.25	1.28	1.48	0.14	-0.20	-0.40	0.33	-0.67	-0.27	-0.88	0.40	0.53	0.72	0.25	1.24	-0.49	-0.17	0.34	0.00	0.32	0.95
TCGA-CN-5374	-0.15	7.56	-0.05	0.53	-0.25	-0.45	-0.09	0.66	4.34	-0.40	-0.79	1.44	-0.27	-0.72	0.34	-1.07	-0.20	-0.77	0.26	-0.49	-0.17	-0.43	0.00	0.45	0.89
TCGA-CN-6010	-0.04	-0.13	2.06	-0.19	-0.25	0.67	-0.41	-1.03	-0.85	-0.40	-0.79	0.60	-0.11	-0.58	-0.17	-0.17	-0.24	-0.51	2.34	-0.49	-0.17	-0.43	0.01	0.25	0.97
TCGA-CN-6011	-0.10	-0.13	-0.86	0.06	-0.25	0.93	-0.10	-1.03	-0.85	-0.40	3.35	-0.28	0.49	0.31	-0.01	0.63	-0.79	-0.77	0.07	-0.49	-0.17	-0.43	0.00	0.33	0.96
TCGA-CN-6012	0.31	-0.13	1.75	0.22	-0.25	-1.07	-0.68	0.51	1.35	-0.40	0.60	-0.80	-0.27	-0.24	0.42	0.63	-0.64	-0.77	-0.47	-0.49	-0.17	0.07	0.00	0.54	0.85
TCGA-CN-6013	-0.28	-0.13	-0.33	0.33	-0.25	0.05	0.97	1.03	-0.18	-0.40	2.48	-0.80	-0.04	-0.40	0.72	0.19	-0.66	-0.01	-0.48	-0.49	-0.17	-0.43	0.00	0.56	0.83
TCGA-CN-6016	-0.46	-0.13	-0.86	-1.00	-0.25	-0.62	-0.93	0.14	2.27	1.53	-0.13	0.72	-0.27	1.41	-0.46	-0.02	-0.70	-0.76	1.76	-0.49	-0.17	-0.43	0.00	0.59	0.81
TCGA-CN-6017	-0.50	-0.13	0.53	2.04	-0.25	-1.13	0.09	0.01	0.18	-0.40	-0.79	1.37	0.13	-0.86	0.81	0.45	-0.47	-0.29	-0.02	-0.49	-0.17	-0.40	0.00	0.55	0.84
TCGA-CN-6018	-0.46	-0.13	0.01	-0.84	-0.25	1.15	-0.56	-1.03	-0.85	-0.40	1.01	-0.60	-0.27	0.21	0.55	-0.62	-0.79	0.69	-0.92	2.05	-0.17	0.36	0.00	0.59	0.81
TCGA-CN-6019	-0.31	-0.13	-0.69	-0.25	-0.25	-0.83	-0.79	-0.16	-0.27	-0.40	-0.79	-0.52	-0.27	2.48	-1.12	0.52	-0.76	-0.69	-0.07	-0.49	-0.17	-0.43	0.00	0.81	0.60
TCGA-CN-6020	-0.50	-0.13	-0.36	-0.88	-0.25	0.18	-0.81	-0.63	-0.40	-0.40	0.23	-0.43	-0.27	1.66	-0.17	-0.82	-0.79	1.02	-0.57	-0.49	-0.17	1.70	0.00	0.41	0.93
TCGA-CN-6021	2.15	-0.13	2.81	0.35	-0.25	-0.66	-0.16	-0.64	0.41	0.08	-0.45	-0.80	0.19	-1.06	-1.43	0.46	-0.38	0.57	-0.92	-0.27	-0.17	3.95	0.01	0.28	0.96
TCGA-CN-6022	-0.50	-0.13	-0.86	-0.87	-0.25	-0.72	-0.93	-0.97	1.29	-0.40	-0.41	-0.69	-0.27	2.03	-0.62	0.75	0.40	-0.77	-0.30	-0.49	-0.17	-0.43	0.00	0.63	0.78
TCGA-CN-6023	0.26	-0.13	-0.28	3.50	-0.25	-1.13	0.61	0.03	1.69	-0.40	0.00	-0.80	-0.27	-0.93	-0.09	0.38	-0.54	-0.58	-0.79	-0.33	-0.17	-0.43	0.00	0.42	0.91
TCGA-CN-6024	-0.50	-0.13	-0.86	-0.93	-0.25	-0.37	-0.93	-0.62	0.76	-0.40	-0.39	-0.80	-0.27	2.44	-0.74	-0.22	0.01	-0.77	-0.92	0.26	-0.17	-0.43	0.07	0.12	1.15
TCGA-CN-6988	-0.50	-0.13	-0.61	-0.48	-0.25	-1.13	-0.93	2.55	-0.85	-0.40	-0.53	-0.80	-0.27	0.57	-1.43	1.27	-0.79	3.62	-0.92	1.50	-0.17	0.17	0.37	0.04	1.12
TCGA-CN-6989	-0.50	-0.13	-0.86	-0.94	-0.25	0.89	-0.86	-1.03	0.12	-0.40	0.51	-0.80	-0.27	2.03	-1.43	-0.76	-0.40	1.58	-0.92	-0.31	-0.17	-0.43	0.00	0.59	0.81
TCGA-CN-6992	0.67	-0.13	-0.48	-0.94	-0.25	-0.56	0.12	-0.41	1.30	-0.40	-0.79	-0.78	-0.27	1.26	-0.68	1.08	-0.40	-0.24	0.27	-0.49	-0.17	-0.43	0.00	0.43	0.91
TCGA-CN-6994	-0.50	-0.13	-0.66	-1.00	-0.25	0.58	-0.93	-0.42	-0.09	-0.40	-0.79	1.14	-0.27	1.36	-1.03	-0.07	-0.11	0.43	0.88	-0.49	-0.17	-0.43	0.00	0.59	0.81
TCGA-CN-6995	-0.34	-0.13	-0.74	1.64	-0.25	-0.29	0.29	0.93	-0.03	-0.40	1.03	1.22	-0.27	-0.58	0.69	0.34	-0.49	-0.43	-0.91	-0.49	-0.17	-0.43	0.00	0.54	0.84
TCGA-CN-69	-0.32	-0.13	-0.20	-0.84	-0.25	1.29	0.91	-0.96	-0.52	-0.40	1.70	-0.80	-0.27	-0.36	0.68	0.45	-0.60	0.08	1.29	-0.49	-0.17	-0.38	0.00	0.44	0.90

96																									
TCGA-CN-6997	1.54	-0.13	-0.18	-1.00	-0.25	-0.26	-0.93	0.21	-0.85	3.30	-0.79	0.18	-0.27	0.75	-0.55	0.91	-0.47	-0.31	1.43	-0.49	-0.17	-0.43	0.02	0.19	1.03
TCGA-CN-6998	-0.44	-0.13	-0.86	-0.41	-0.25	0.99	0.11	-1.03	-0.85	-0.40	0.05	0.34	-0.27	0.78	1.00	-0.30	-0.23	-0.49	-0.92	-0.17	-0.17	-0.15	0.02	0.19	1.05
TCGA-CN-A497	1.81	-0.13	0.93	-0.43	-0.25	1.30	-0.21	1.28	-0.12	-0.40	0.51	-0.80	-0.27	-0.55	-1.07	-0.77	-0.45	0.56	-0.92	1.16	-0.17	-0.25	0.01	0.23	0.98
TCGA-CN-A498	-0.15	-0.13	2.26	-0.37	-0.25	-1.13	0.67	1.53	-0.72	-0.40	-0.79	0.36	-0.27	-0.50	0.04	-0.83	0.69	0.73	0.31	-0.49	-0.17	-0.26	0.00	0.32	0.95
TCGA-CN-A499	0.04	-0.13	-0.08	1.08	-0.25	-0.46	1.89	0.16	-0.85	2.38	-0.75	0.63	-0.27	-1.12	1.91	0.06	-0.79	0.23	-0.24	-0.49	-0.17	-0.05	0.00	0.36	0.95
TCGA-CN-A49A	10.41	-0.13	-0.68	-0.93	-0.25	-0.72	-0.34	-1.03	-0.26	-0.40	0.95	-0.80	-0.27	1.04	-0.57	-1.09	-0.79	-0.77	-0.92	0.68	-0.17	-0.43	0.05	0.15	1.06
TCGA-CN-A49B	-0.35	-0.13	-0.86	0.36	-0.25	-0.24	1.15	2.20	-0.77	2.11	-0.52	-0.80	-0.27	-0.24	1.20	0.81	-0.70	-0.31	-0.25	-0.49	-0.17	-0.43	0.00	0.50	0.87
TCGA-CN-A49C	-0.48	-0.13	0.16	0.21	-0.25	-0.39	-0.93	1.17	0.99	-0.40	-0.79	2.94	0.94	-1.04	0.14	-0.24	1.23	0.06	1.29	-0.49	-0.17	-0.43	0.11	0.10	1.05
TCGA-CN-A63T	0.10	-0.13	-0.34	0.00	-0.25	0.76	1.48	-1.03	-0.74	-0.40	2.22	-0.80	-0.27	-0.38	1.27	-0.11	-0.73	0.07	-0.92	0.03	-0.17	-0.15	0.00	0.49	0.87
TCGA-CN-A63U	-0.50	0.33	0.50	-0.46	-0.25	-0.44	-0.93	2.70	0.93	-0.40	1.69	-0.80	1.16	-0.58	0.46	-1.05	-0.58	-0.03	0.82	1.92	-0.17	-0.03	0.02	0.20	1.01
TCGA-CN-A63V	-0.41	-0.13	-0.48	-0.89	-0.25	-1.13	-0.93	-0.16	1.53	4.22	-0.79	-0.63	-0.27	2.29	-1.10	-0.34	-0.56	-0.77	-0.75	0.44	-0.17	0.15	0.00	0.30	1.02
TCGA-CN-A63W	-0.46	-0.13	-0.52	0.31	-0.25	-1.13	-0.23	0.47	-0.28	-0.40	-0.35	0.55	-0.27	1.17	0.83	0.46	-0.74	-0.77	-0.80	-0.49	-0.17	-0.43	0.01	0.23	1.04
TCGA-CN-A641	-0.46	-0.13	1.80	-1.00	4.34	-0.47	-0.86	0.23	-0.79	2.22	0.07	-0.80	-0.27	-0.31	-0.92	-0.23	1.14	0.00	0.35	0.22	-0.17	0.56	0.20	0.07	1.04
TCGA-CN-A642	-0.43	-0.13	-0.86	1.53	-0.25	-1.13	2.98	-0.10	-0.24	-0.40	0.03	-0.10	-0.27	-1.38	1.42	0.16	1.64	-0.77	0.61	-0.49	-0.17	-0.43	0.00	0.46	0.89
TCGA-CN-A6UY	-0.50	-0.13	0.01	1.94	-0.25	-0.79	-0.48	1.09	2.81	-0.40	-0.15	1.69	-0.27	-0.19	-1.03	-1.02	-0.79	0.60	-0.29	-0.49	-0.17	-0.43	0.00	0.38	0.93
TCGA-CN-A6V3	-0.41	-0.13	0.95	1.32	-0.25	-1.13	0.96	0.02	-0.84	-0.40	-0.79	-0.30	-0.27	-0.56	2.70	-0.72	-0.55	-0.40	-0.18	-0.49	-0.17	-0.43	0.00	0.43	0.91
TCGA-CN-A6V6	-0.50	-0.13	0.63	2.88	-0.25	-1.13	1.03	1.19	0.71	3.84	-0.77	-0.80	-0.27	-0.83	0.26	-0.78	-0.79	-0.62	-0.53	-0.49	-0.17	-0.43	0.00	0.36	0.94
TCGA-CN-A6V7	0.89	-0.13	-0.56	1.77	-0.25	-1.13	3.34	1.63	0.52	-0.40	1.10	-0.80	0.46	-1.18	0.30	-0.11	-0.45	0.20	-0.92	-0.19	-0.17	-0.43	0.09	0.11	1.05
TCGA-CQ-5323	-0.50	-0.13	1.29	0.54	-0.25	-1.13	-0.09	2.20	-0.55	3.55	-0.79	-0.27	-0.27	-0.53	0.56	-0.28	-0.64	0.49	0.49	-0.49	-0.17	-0.27	0.00	0.33	0.95
TCGA-CQ-5324	-0.38	-0.13	-0.13	0.71	-0.25	-1.13	0.03	1.28	-0.59	-0.40	1.97	-0.07	-0.27	0.08	0.24	-0.28	-0.63	0.39	0.96	-0.49	-0.17	-0.43	0.00	0.37	0.93
TCGA-CQ-53	-0.07	-0.13	0.98	1.11	-0.25	-0.43	-0.05	-0.91	-0.50	0.05	-0.63	-0.27	-0.27	-0.61	0.87	0.52	-0.56	-0.48	1.21	-0.49	-0.17	-0.43	0.00	0.47	0.88

25																									
TCGA-CQ-53 26	-0.50	-0.13	-0.86	-1.00	-0.25	-0.78	-0.93	-0.55	-0.32	-0.38	0.23	-0.80	-0.27	1.88	-0.90	-0.78	-0.79	-0.58	-0.92	4.72	2.30	0.86	0.00	0.64	0.77
TCGA-CQ-53 27	-0.20	-0.13	-0.61	2.24	-0.25	-1.13	0.56	-0.93	-0.40	-0.40	-0.05	-0.05	-0.27	-0.78	0.83	1.47	-0.30	-0.67	0.98	-0.49	-0.17	-0.06	0.00	0.37	0.93
TCGA-CQ-53 29	-0.47	-0.13	-0.79	-0.37	-0.25	1.58	1.02	-1.03	-0.79	-0.40	0.44	-0.47	-0.27	-0.07	0.75	1.58	-0.75	-0.54	-0.84	-0.39	-0.17	-0.38	0.00	0.51	0.86
TCGA-CQ-53 30	0.23	-0.13	-0.83	-0.47	-0.25	1.47	0.77	-1.03	-0.72	-0.40	1.57	-0.74	0.27	-0.43	0.98	1.27	-0.52	-0.77	0.37	-0.49	-0.17	-0.37	0.00	0.43	0.90
TCGA-CQ-53 31	0.74	-0.13	-0.01	0.42	-0.25	-0.33	0.84	-0.05	-0.26	0.33	0.43	0.54	-0.27	-1.11	2.09	0.74	-0.53	-0.35	-0.16	-0.49	-0.17	0.02	0.00	0.50	0.87
TCGA-CQ-53 32	1.21	-0.13	0.09	-1.00	-0.25	0.37	-0.86	-1.03	0.09	-0.31	0.57	-0.80	-0.27	1.19	-1.43	0.23	-0.78	-0.22	-0.92	1.90	-0.17	-0.43	0.00	0.48	0.88
TCGA-CQ-53 33	-0.45	-0.13	-0.68	0.40	-0.25	-0.27	0.14	0.51	1.55	-0.40	0.16	-0.35	-0.27	-0.66	0.83	1.70	0.18	-0.77	-0.08	-0.49	-0.17	-0.36	0.00	0.40	0.92
TCGA-CQ-53 34	0.28	-0.13	-0.58	1.38	-0.25	-0.52	1.82	-1.02	0.05	-0.40	2.29	-0.80	-0.14	-0.77	1.71	0.15	-0.63	-0.72	-0.07	-0.49	-0.17	-0.43	0.00	0.55	0.84
TCGA-CQ-62 18	-0.16	-0.13	-0.39	-0.52	-0.25	2.17	-0.40	-1.03	-0.85	-0.40	0.33	-0.54	-0.27	0.19	-0.02	0.58	-0.44	-0.77	0.64	-0.49	-0.17	-0.30	0.01	0.26	0.99
TCGA-CQ-62 19	-0.12	-0.13	-0.86	0.80	-0.25	-0.76	0.29	0.83	-0.76	2.00	-0.79	0.39	-0.27	-0.47	1.50	0.22	0.23	-0.77	1.58	-0.49	-0.17	-0.43	0.00	0.42	0.91
TCGA-CQ-62 20	0.04	-0.13	-0.80	-0.59	-0.25	-1.13	0.77	0.98	-0.85	0.04	-0.79	0.50	-0.27	0.31	0.65	1.15	-0.33	1.78	-0.35	-0.49	-0.17	-0.16	0.01	0.26	1.01
TCGA-CQ-62 21	-0.50	-0.13	-0.83	-1.00	-0.25	0.94	-0.93	-1.03	-0.85	-0.40	1.26	-0.41	-0.27	0.82	-1.14	3.06	-0.79	-0.62	-0.92	0.95	-0.17	-0.43	0.00	0.69	0.73
TCGA-CQ-62 22	-0.48	-0.13	-0.86	0.57	-0.25	-0.91	-0.79	0.07	0.05	-0.40	-0.79	0.45	-0.27	1.30	0.31	-0.44	-0.13	-0.74	0.71	-0.49	-0.17	-0.43	0.00	0.58	0.81
TCGA-CQ-62 23	0.16	-0.13	0.03	-1.00	-0.25	-0.71	0.36	0.91	-0.54	3.19	0.37	-0.80	-0.27	0.74	-0.32	-0.55	-0.61	2.00	0.21	-0.49	-0.17	-0.19	0.01	0.28	0.98
TCGA-CQ-62 24	-0.35	-0.13	-0.86	-0.91	-0.25	2.30	-0.93	-1.03	-0.08	-0.40	0.00	-0.63	-0.27	-0.06	0.25	0.19	1.77	-0.70	0.01	-0.49	-0.17	-0.43	0.00	0.56	0.83
TCGA-CQ-62 25	-0.50	-0.13	-0.84	-0.73	4.57	-1.13	-0.93	-1.03	2.53	-0.40	3.13	-0.80	-0.27	1.51	-1.24	-0.61	-0.55	-0.51	-0.92	1.60	0.61	-0.43	0.03	0.18	1.06
TCGA-CQ-62 27	0.55	-0.13	-0.60	-0.77	0.46	-0.62	-0.48	-1.03	0.75	-0.40	-0.79	-0.80	-0.27	1.18	0.76	0.78	0.12	-0.73	-0.30	-0.49	-0.17	-0.43	0.00	0.32	0.98
TCGA-CQ-62 28	0.77	-0.13	0.13	-1.00	0.49	-1.10	-0.72	-0.29	0.24	1.32	0.70	-0.80	-0.27	1.52	-0.85	0.82	-0.64	-0.29	-0.92	0.27	-0.17	-0.43	0.00	0.36	0.96
TCGA-CQ-62 29	0.35	-0.13	-0.43	0.47	-0.25	-1.13	1.72	0.17	-0.85	-0.40	-0.79	0.21	-0.27	-0.70	1.23	0.36	0.66	-0.41	2.07	-0.49	-0.17	-0.43	0.00	0.38	0.93
TCGA-CQ-70 63	0.34	-0.13	-0.86	0.49	-0.25	0.04	1.24	1.27	-0.73	0.24	-0.79	0.91	-0.27	-0.52	0.72	-0.58	0.60	-0.57	1.02	-0.49	-0.17	-0.43	0.00	0.38	0.93
TCGA-CQ-70	-0.50	-0.13	-0.51	-1.00	-0.25	0.03	-0.93	0.86	-0.32	-0.40	-0.79	0.17	-0.27	-0.23	-1.17	-1.19	2.57	3.50	2.43	-0.49	-0.17	-0.43	0.23	0.07	1.07

65																									
TCGA-CQ-70 68	-0.43	-0.13	-0.78	2.32	-0.25	-1.13	0.75	0.93	0.49	-0.40	1.43	1.31	-0.27	-0.70	1.36	-0.19	-0.62	-0.77	-0.92	-0.49	-0.17	-0.43	0.00	0.30	0.99
TCGA-CQ-70 69	-0.34	-0.13	-0.42	-0.95	-0.06	0.61	-0.93	-1.03	-0.85	-0.40	0.38	-0.80	-0.27	0.70	-1.04	-1.44	-0.79	4.78	-0.92	2.49	-0.17	-0.14	0.00	0.51	0.86
TCGA-CQ-70 71	-0.50	-0.13	0.13	0.04	-0.25	-0.02	0.35	-0.44	-0.79	-0.40	-0.79	0.03	-0.27	-0.24	1.42	0.60	-0.05	-0.38	0.53	-0.49	-0.17	-0.30	0.00	0.35	0.95
TCGA-CQ-70 72	-0.44	-0.13	-0.75	-1.00	0.39	-0.31	-0.93	-0.53	1.25	-0.40	-0.50	-0.19	-0.27	0.81	-1.43	1.45	0.84	1.95	-0.60	-0.49	-0.17	-0.43	0.00	0.63	0.78
TCGA-CQ-A4 C6	0.21	-0.13	-0.86	-1.00	-0.25	0.82	0.32	-0.23	-0.44	0.09	0.74	-0.52	-0.27	-0.05	1.86	-0.09	-0.04	0.36	0.10	-0.49	-0.17	-0.39	0.00	0.43	0.91
TCGA-CQ-A4 C7	-0.50	-0.13	-0.83	-0.51	-0.25	-0.03	-0.93	-0.89	-0.81	-0.40	-0.79	1.38	-0.27	0.33	2.65	-0.25	-0.79	-0.73	-0.92	2.75	-0.17	-0.35	0.00	0.53	0.86
TCGA-CQ-A4 C9	-0.50	-0.13	1.80	-0.81	-0.25	-0.91	-0.93	-0.19	0.68	-0.09	-0.79	-0.80	-0.27	1.03	-0.69	0.39	-0.12	-0.26	-0.06	-0.40	-0.17	-0.43	0.01	0.25	1.00
TCGA-CQ-A4 CB	0.32	-0.13	-0.86	-1.00	-0.25	0.96	-0.30	-1.03	-0.85	-0.40	0.57	-0.80	0.29	0.14	0.34	0.21	-0.79	2.76	-0.92	1.20	-0.17	0.10	0.00	0.31	0.98
TCGA-CQ-A4 CD	-0.01	-0.13	-0.82	-0.46	-0.25	1.17	-0.92	-1.03	-0.85	-0.40	-0.02	0.22	-0.21	-0.46	2.04	-0.73	-0.79	1.34	-0.92	2.79	-0.17	-0.10	0.00	0.40	0.94
TCGA-CQ-A4 CE	-0.50	-0.13	0.16	0.91	-0.25	-1.13	1.43	0.67	-0.10	0.37	-0.79	0.15	-0.27	-0.40	1.18	-1.06	-0.02	0.75	0.17	-0.49	-0.17	-0.04	0.05	0.14	1.05
TCGA-CQ-A4 CG	-0.50	-0.13	-0.53	-1.00	-0.25	2.14	-0.93	-1.03	-0.85	-0.40	-0.79	0.98	-0.27	0.30	1.81	-1.43	-0.08	-0.14	-0.92	1.07	-0.17	-0.43	0.05	0.14	1.08
TCGA-CQ-A4 CH	0.46	-0.13	0.04	-1.00	0.85	-0.91	-0.93	-0.44	-0.45	-0.40	0.46	-0.80	-0.27	2.29	-1.01	-0.82	-0.79	-0.46	-0.92	1.13	-0.17	0.97	0.00	0.74	0.68
TCGA-CQ-A4 CI	1.02	-0.13	-0.71	-0.43	-0.25	-1.05	-0.93	0.86	0.83	-0.40	0.54	-0.80	-0.27	1.40	-1.43	0.78	-0.31	0.46	-0.92	0.28	-0.17	-0.43	0.00	0.48	0.88
TCGA-CR-52 43	-0.20	-0.13	1.10	0.15	-0.25	1.55	-0.93	-0.67	3.64	-0.40	-0.52	-0.03	-0.27	-0.61	-0.62	-1.48	-0.29	-0.23	1.28	-0.49	-0.17	-0.43	0.00	0.29	0.96
TCGA-CR-52 47	-0.50	-0.13	0.01	-1.00	2.77	0.56	-0.93	-1.03	-0.85	-0.40	-0.79	-0.80	-0.27	2.20	-1.24	-0.78	-0.79	-0.77	0.75	0.09	-0.17	-0.43	0.00	0.47	0.90
TCGA-CR-52 48	-0.06	-0.13	0.57	0.93	-0.25	-1.13	1.50	0.47	2.62	0.66	1.19	-0.11	-0.27	-0.17	-0.76	-0.39	-0.74	-0.77	-0.16	-0.49	-0.17	-0.43	0.00	0.46	0.89
TCGA-CR-52 49	3.85	-0.13	0.30	0.39	-0.25	0.90	-0.93	1.30	3.07	-0.40	-0.79	-0.35	0.19	-1.06	-0.47	-1.19	-0.10	0.32	0.02	-0.49	-0.17	-0.43	0.00	0.48	0.88
TCGA-CR-52 50	-0.07	-0.13	0.76	-0.67	-0.25	1.50	-0.76	1.00	4.39	-0.40	-0.79	0.02	-0.27	0.00	-0.93	-1.71	-0.40	-0.58	0.73	-0.49	-0.17	-0.43	0.00	0.40	0.92
TCGA-CR-64 67	1.23	-0.13	0.21	3.41	-0.25	-1.13	0.28	0.16	2.36	-0.40	-0.79	-0.54	0.85	-1.01	0.35	-1.12	-0.58	-0.61	-0.13	-0.49	-0.17	-0.43	0.00	0.51	0.86
TCGA-CR-64 70	1.22	-0.13	0.14	1.14	-0.25	-0.28	0.45	0.93	3.18	-0.40	-0.79	0.67	-0.27	-0.74	-0.16	-1.05	-0.36	-0.75	0.85	-0.49	-0.17	-0.43	0.00	0.30	0.96
TCGA-CR-64	0.26	-0.13	-0.37	1.38	-0.25	0.47	1.73	-0.77	0.87	-0.40	0.03	-0.80	-0.27	-0.76	-0.54	-0.62	0.63	-0.07	0.13	-0.49	-0.17	-0.05	0.00	0.40	0.92

71																									
TCGA-CR-6472	0.21	-0.13	0.43	0.64	-0.25	0.34	-0.76	0.86	2.57	0.94	-0.79	1.45	-0.27	-1.23	0.65	-0.81	-0.39	-0.66	1.95	-0.49	-0.17	-0.43	0.01	0.23	1.00
TCGA-CR-6473	0.44	-0.13	0.00	-0.26	-0.25	1.17	-0.33	2.64	1.73	-0.40	0.15	-0.79	0.49	-1.20	0.81	-1.71	-0.45	3.15	-0.38	-0.49	-0.17	-0.43	0.02	0.20	1.03
TCGA-CR-6474	-0.49	-0.13	-0.38	-0.53	-0.25	1.28	0.69	-1.03	-0.85	-0.40	1.97	-0.80	-0.09	0.33	-0.19	-0.75	-0.73	0.18	-0.92	0.55	-0.17	2.64	0.00	0.40	0.92
TCGA-CR-6477	-0.50	-0.13	-0.26	-0.89	-0.25	1.76	-0.47	-1.03	-0.18	-0.40	0.71	-0.80	-0.27	0.32	-1.26	-0.52	0.20	3.06	-0.88	-0.19	-0.17	-0.20	0.00	0.49	0.87
TCGA-CR-6478	0.11	-0.13	-0.25	0.34	-0.25	1.12	0.05	-1.03	-0.64	-0.40	-0.79	1.65	-0.27	-0.33	1.92	-0.29	-0.72	-0.55	-0.71	-0.49	-0.17	0.05	0.00	0.42	0.91
TCGA-CR-6480	-0.50	-0.13	1.16	0.56	-0.25	-0.94	0.51	1.72	0.37	0.98	-0.79	-0.80	-0.27	-1.28	0.03	-0.19	-0.77	4.26	0.31	-0.49	-0.17	-0.15	0.00	0.35	0.94
TCGA-CR-6481	0.65	1.76	-0.20	1.03	-0.25	-1.13	0.73	-0.06	2.09	1.38	-0.79	-0.22	-0.27	0.11	-0.04	-0.54	-0.65	-0.77	0.88	-0.49	-0.17	-0.43	0.00	0.53	0.85
TCGA-CR-6482	2.11	2.40	0.36	0.25	-0.25	-0.64	-0.43	0.40	4.36	-0.40	-0.79	-0.65	-0.27	-0.71	-0.02	-0.25	-0.21	-0.01	0.47	-0.49	-0.17	-0.39	0.00	0.37	0.93
TCGA-CR-6484	-0.26	-0.13	-0.02	-0.29	-0.25	0.07	-0.47	0.77	0.51	-0.40	-0.79	0.55	-0.27	0.53	0.11	-0.04	-0.54	-0.77	0.56	-0.49	-0.17	0.45	0.00	0.67	0.76
TCGA-CR-6487	0.00	-0.13	0.10	3.37	-0.25	-1.13	1.71	0.64	0.87	-0.40	-0.63	-0.75	-0.27	-1.09	0.01	-0.14	-0.47	-0.66	-0.01	-0.49	-0.17	-0.43	0.00	0.49	0.87
TCGA-CR-6488	-0.50	-0.13	-0.84	-1.00	-0.25	-0.39	-0.91	-0.43	0.19	-0.40	-0.38	-0.80	-0.27	2.76	-0.90	0.02	-0.52	-0.77	-0.02	-0.49	-0.17	-0.43	0.00	0.31	1.05
TCGA-CR-6491	-0.50	-0.13	1.15	1.15	-0.25	-1.13	0.35	-0.24	-0.51	-0.40	-0.79	2.30	-0.27	-0.27	0.01	-1.25	-0.20	0.09	-0.92	1.49	-0.17	-0.43	0.09	0.11	1.05
TCGA-CR-6492	0.86	-0.13	0.81	-0.86	-0.25	1.21	0.34	-1.03	-0.85	-0.40	-0.17	-0.14	-0.23	-0.64	0.95	-0.02	-0.46	-0.28	1.20	-0.49	-0.17	1.29	0.00	0.33	0.95
TCGA-CR-6493	0.18	-0.13	0.12	-0.40	1.59	-0.79	0.66	-1.03	-0.71	-0.40	-0.53	-0.35	-0.27	0.85	0.14	0.37	-0.79	-0.58	-0.92	1.10	-0.17	0.66	0.02	0.19	1.04
TCGA-CR-7364	-0.50	-0.13	1.61	-0.61	-0.25	-0.08	0.12	0.06	-0.26	-0.40	-0.27	-0.80	-0.09	-0.57	-0.70	-0.59	0.23	2.35	0.96	-0.49	-0.17	2.01	0.00	0.30	0.96
TCGA-CR-7365	-0.50	-0.13	-0.60	-1.00	-0.25	1.50	-0.75	-1.03	-0.85	-0.40	0.53	-0.80	-0.27	1.58	0.01	0.04	-0.73	-0.77	-0.22	-0.49	-0.17	-0.43	0.00	0.63	0.77
TCGA-CR-7367	-0.27	-0.13	0.54	-0.26	-0.25	1.37	0.19	-1.03	-0.85	-0.40	-0.03	-0.80	-0.27	0.31	-0.05	-0.09	-0.54	-0.77	0.42	-0.49	-0.17	0.06	0.00	0.43	0.90
TCGA-CR-7368	-0.50	-0.13	0.82	-0.73	-0.25	0.53	1.51	-0.62	-0.23	-0.40	1.40	-0.57	-0.27	0.11	-0.49	-0.35	-0.54	-0.03	0.88	-0.49	-0.17	-0.36	0.01	0.23	0.99
TCGA-CR-7369	0.77	-0.13	0.91	-0.25	-0.25	0.35	0.23	-1.03	-0.85	-0.40	-0.79	-0.80	-0.27	0.43	-0.85	-0.13	-0.16	0.01	-0.92	0.92	-0.17	0.14	0.00	0.38	0.93
TCGA-CR-7370	0.45	-0.13	0.79	-0.24	-0.25	-0.75	1.25	-0.28	-0.60	-0.40	-0.79	0.70	-0.27	-0.48	0.29	0.81	-0.31	0.16	1.52	-0.49	-0.17	-0.43	0.00	0.31	0.96
TCGA-CR-73	-0.13	-0.13	-0.09	0.02	-0.25	1.48	0.69	-1.03	-0.11	-0.40	0.56	-0.80	-0.27	-0.19	-0.67	-0.64	-0.79	0.86	-0.92	0.28	-0.17	3.39	0.00	0.45	0.89

71																									
TCGA-CR-73 72	2.38	-0.13	-0.04	-1.00	-0.25	1.07	-0.62	-0.95	-0.03	-0.40	0.07	-0.80	-0.27	0.14	-0.87	0.91	-0.45	0.81	-0.08	0.01	-0.17	-0.09	0.04	0.16	1.03
TCGA-CR-73 73	-0.20	-0.13	-0.25	-0.53	-0.25	-0.05	1.56	-0.49	-0.65	-0.40	2.39	-0.80	-0.27	0.27	0.72	0.06	-0.71	0.61	-0.62	-0.49	-0.17	-0.42	0.00	0.42	0.91
TCGA-CR-73 76	-0.33	-0.13	-0.55	0.26	-0.25	0.41	1.28	0.63	-0.63	1.58	0.22	-0.80	-0.27	-0.19	0.75	0.48	-0.60	-0.77	0.01	-0.49	-0.17	-0.25	0.00	0.52	0.85
TCGA-CR-73 77	0.37	-0.13	-0.27	1.12	-0.25	0.62	0.10	-0.51	0.36	-0.40	0.33	-0.80	-0.27	-0.79	1.01	-0.19	0.02	-0.53	0.51	-0.49	-0.17	-0.43	0.00	0.58	0.82
TCGA-CR-73 79	-0.50	-0.13	0.76	-1.00	-0.25	0.99	-0.88	-1.03	-0.85	-0.40	0.87	-0.80	-0.27	0.94	-0.74	-0.43	-0.74	-0.77	-0.92	0.63	-0.17	4.07	0.00	0.40	0.92
TCGA-CR-73 80	-0.07	-0.13	-0.31	-1.00	-0.25	1.82	-0.83	-1.03	-0.09	-0.40	0.60	-0.80	-0.27	-0.40	0.85	0.42	0.01	-0.77	2.67	-0.49	-0.17	-0.09	0.00	0.41	0.91
TCGA-CR-73 82	0.16	-0.13	-0.70	-0.65	-0.25	0.35	1.10	-0.46	-0.85	-0.40	1.55	-0.78	0.73	-0.49	2.11	0.83	-0.79	-0.73	-0.92	0.96	-0.17	1.05	0.00	0.47	0.88
TCGA-CR-73 83	-0.50	-0.13	-0.19	-0.86	-0.25	0.40	-0.30	-0.17	-0.07	-0.03	-0.79	1.16	-0.27	-0.36	0.25	1.05	0.73	-0.73	2.02	-0.49	-0.17	-0.10	0.00	0.44	0.90
TCGA-CR-73 85	-0.13	2.36	-0.23	0.40	-0.25	0.49	-0.07	0.71	2.68	-0.40	-0.35	-0.77	-0.03	-0.81	0.78	-0.61	0.57	-0.07	-0.87	-0.49	-0.17	0.03	0.00	0.49	0.87
TCGA-CR-73 86	-0.25	-0.13	-0.86	-0.02	-0.25	0.33	-0.32	0.28	-0.39	-0.40	-0.79	-0.43	-0.27	-0.04	0.68	0.90	0.09	-0.77	1.99	-0.49	-0.17	-0.17	0.00	0.53	0.85
TCGA-CR-73 88	-0.50	-0.13	1.59	-0.73	-0.25	0.44	0.40	0.22	-0.15	-0.40	-0.79	-0.80	-0.27	-0.10	-0.77	-0.34	-0.53	0.43	-0.92	0.39	-0.17	3.95	0.00	0.40	0.92
TCGA-CR-73 89	-0.50	-0.13	2.26	-0.91	-0.25	-0.01	0.90	-1.03	-0.70	-0.40	0.07	-0.80	1.74	-1.24	-0.90	1.69	1.04	0.63	0.56	-0.49	-0.17	0.44	0.01	0.28	0.96
TCGA-CR-73 90	-0.50	-0.13	-0.54	-1.00	1.19	1.11	-0.93	-1.03	-0.71	-0.40	0.33	-0.80	-0.27	1.52	-1.38	-1.22	-0.79	-0.73	-0.92	1.76	-0.17	5.39	0.01	0.25	1.03
TCGA-CR-73 91	-0.45	-0.13	-0.16	0.80	-0.25	-0.23	-0.10	-0.37	1.78	-0.40	-0.79	-0.66	-0.27	-0.01	-0.45	-0.38	0.22	0.32	0.72	-0.49	-0.17	0.47	0.00	0.55	0.84
TCGA-CR-73 92	-0.42	-0.13	0.61	0.06	-0.25	1.65	0.05	-0.91	-0.29	-0.40	0.88	-0.80	-0.27	-0.01	-0.06	-0.60	-0.77	0.11	-0.36	-0.49	-0.17	-0.08	0.00	0.45	0.89
TCGA-CR-73 93	1.10	-0.13	-0.48	0.75	-0.25	1.26	0.65	-1.03	1.66	-0.40	-0.79	0.49	0.25	-1.13	-1.04	0.26	-0.05	0.75	0.56	-0.49	-0.17	0.54	0.02	0.19	1.01
TCGA-CR-73 94	-0.10	-0.13	0.55	1.99	-0.25	-0.97	2.09	-0.28	-0.55	2.62	1.10	-0.80	-0.27	-0.80	-0.24	-0.11	-0.57	-0.22	-0.16	-0.49	-0.17	0.09	0.00	0.40	0.92
TCGA-CR-73 95	-0.23	-0.13	0.05	0.35	-0.25	-0.40	1.38	-0.11	-0.05	-0.40	1.06	0.04	-0.27	-0.52	0.07	0.18	-0.73	0.87	1.01	-0.49	-0.17	0.10	0.00	0.43	0.90
TCGA-CR-73 97	-0.43	-0.13	-0.86	-1.00	-0.25	1.41	-0.24	0.16	0.32	-0.40	0.23	-0.80	-0.27	0.05	1.74	-0.86	0.64	-0.76	0.94	-0.49	-0.17	-0.41	0.00	0.54	0.84
TCGA-CR-73 98	-0.48	-0.13	-0.56	-0.60	-0.25	0.88	0.30	-1.03	-0.85	-0.40	0.89	-0.80	-0.27	0.15	-1.11	0.83	-0.79	0.32	3.77	-0.49	-0.17	0.82	0.01	0.28	0.98
TCGA-CR-73	0.86	-0.13	-0.81	2.11	-0.25	-1.13	2.73	-0.56	0.17	-0.40	0.14	-0.80	-0.27	-0.98	1.96	-0.21	-0.28	-0.77	0.20	-0.49	-0.17	-0.36	0.00	0.55	0.83

99																									
TCGA-CR-7401	0.07	-0.13	-0.86	-0.68	-0.25	0.78	0.03	-0.67	0.24	-0.40	-0.79	-0.80	0.19	-0.55	0.84	-0.26	0.47	2.06	2.15	-0.49	-0.17	-0.02	0.00	0.36	0.94
TCGA-CR-7402	-0.50	-0.13	2.14	-0.11	-0.25	0.06	1.63	-0.25	-0.64	-0.40	-0.79	1.22	0.02	-0.39	-0.84	-1.09	-0.58	-0.17	1.88	-0.49	-0.17	0.07	0.03	0.18	1.00
TCGA-CR-7404	-0.23	0.04	0.78	0.42	-0.25	-0.45	0.52	0.42	0.71	-0.40	0.76	-0.80	-0.27	-0.45	0.51	0.11	-0.79	-0.31	0.98	-0.49	-0.17	-0.43	0.00	0.45	0.89
TCGA-CV-5430	-0.05	-0.13	0.07	-0.54	-0.25	0.14	0.04	0.21	0.56	-0.40	0.13	-0.80	-0.27	-0.78	1.47	-0.03	1.15	-0.27	1.17	-0.49	-0.17	-0.43	0.00	0.43	0.90
TCGA-CV-5431	-0.50	-0.13	0.50	0.20	-0.25	0.78	-0.22	1.18	0.81	-0.40	-0.79	0.33	-0.27	-0.32	-0.08	-1.20	-0.19	-0.77	-0.92	1.88	-0.17	-0.36	0.01	0.28	0.97
TCGA-CV-5432	0.30	-0.13	2.93	0.02	-0.25	-0.92	0.12	-0.28	-0.85	-0.40	-0.79	-0.06	-0.19	-0.70	0.34	-0.01	0.70	-0.77	0.58	-0.49	-0.17	-0.43	0.09	0.11	1.03
TCGA-CV-5434	0.28	-0.13	-0.86	-0.85	-0.25	-0.15	-0.81	-0.55	1.03	1.27	-0.79	-0.80	-0.27	1.54	0.05	0.57	-0.79	-0.18	0.03	-0.49	-0.17	-0.43	0.00	0.76	0.67
TCGA-CV-5435	-0.50	-0.13	-0.80	-0.71	-0.25	2.23	-0.93	-1.03	-0.85	-0.40	-0.79	1.77	-0.27	0.90	-0.82	0.06	-0.34	-0.77	0.80	-0.49	-0.17	-0.43	0.00	0.46	0.89
TCGA-CV-5436	-0.50	-0.13	0.17	0.33	-0.25	0.40	-0.77	-1.03	-0.85	-0.40	-0.79	0.22	-0.27	0.00	1.46	-0.16	-0.61	0.72	0.36	-0.49	-0.17	-0.41	0.00	0.39	0.93
TCGA-CV-5439	1.46	-0.13	-0.39	0.57	-0.25	-0.59	1.31	0.89	-0.60	-0.40	-0.79	0.33	-0.16	-0.83	1.63	-0.72	0.60	-0.70	1.37	-0.49	-0.17	-0.43	0.01	0.24	1.00
TCGA-CV-5440	-0.09	-0.13	0.33	-0.53	-0.25	-1.13	-0.93	0.17	-0.66	-0.40	-0.75	0.12	-0.27	0.68	1.05	-0.14	1.16	-0.77	0.57	-0.49	-0.17	-0.43	0.02	0.21	1.03
TCGA-CV-5441	0.12	-0.13	-0.03	0.77	-0.25	-0.57	-0.19	1.59	0.23	-0.40	-0.79	1.02	-0.27	-0.25	0.77	0.10	-0.01	-0.77	-0.48	-0.49	-0.17	-0.26	0.00	0.28	0.97
TCGA-CV-5442	-0.50	-0.13	-0.58	0.33	-0.25	-1.13	1.11	2.60	-0.48	-0.40	1.35	-0.80	0.02	-0.06	-1.25	-0.77	1.83	0.50	-0.92	0.44	-0.17	0.92	0.35	0.04	1.07
TCGA-CV-5443	-0.39	-0.13	1.06	0.95	-0.25	-1.13	0.82	-0.11	0.96	-0.40	0.60	-0.80	-0.27	-0.17	0.27	0.34	-0.71	-0.77	-0.07	-0.49	-0.17	-0.39	0.00	0.52	0.86
TCGA-CV-5444	-0.27	-0.13	-0.86	-1.00	-0.25	0.45	-0.71	-0.18	0.57	1.41	1.26	-0.80	-0.27	0.03	0.92	0.90	0.45	-0.77	0.92	-0.49	-0.17	-0.37	0.00	0.47	0.88
TCGA-CV-5966	-0.50	-0.13	-0.86	0.09	-0.25	0.68	2.39	-1.03	-0.85	-0.40	0.59	0.05	0.67	-0.93	0.14	1.72	-0.66	1.43	0.11	-0.49	0.29	-0.36	0.00	0.31	0.96
TCGA-CV-5970	-0.50	-0.13	1.03	-0.76	-0.25	-0.04	-0.58	-1.03	-0.78	-0.40	-0.77	-0.46	-0.27	0.20	-0.05	-0.16	1.35	0.47	0.67	-0.49	-0.17	0.29	0.01	0.24	0.99
TCGA-CV-5971	0.08	-0.13	-0.35	-0.10	-0.25	0.16	1.04	0.65	-0.64	1.62	-0.79	-0.40	1.37	-0.40	0.49	0.47	-0.59	0.31	1.04	-0.49	-0.17	-0.36	0.00	0.40	0.92
TCGA-CV-5973	-0.50	-0.13	-0.53	-0.86	-0.25	-0.52	-0.93	-0.54	2.97	-0.40	0.16	-0.80	-0.27	1.47	-0.85	0.31	-0.64	-0.44	-0.92	1.11	-0.17	0.28	0.24	0.06	1.13
TCGA-CV-5976	-0.50	-0.13	-0.86	-1.00	-0.25	1.43	-0.93	-1.03	-0.77	-0.40	2.47	-0.80	-0.27	1.23	0.12	0.27	-0.23	-0.77	-0.92	-0.39	-0.17	-0.43	0.00	0.36	0.96
TCGA-CV-59	-0.50	-0.13	-0.58	-1.00	2.11	1.36	-0.93	-1.03	-0.85	-0.40	0.93	1.22	-0.27	-0.16	2.45	-0.89	-0.49	-0.39	-0.92	1.43	-0.17	-0.43	0.00	0.36	0.96

77																									
TCGA-CV-59 78	-0.50	0.06	-0.80	-0.94	-0.25	-1.01	-0.74	-0.52	-0.58	-0.40	-0.79	-0.67	-0.27	2.99	-1.43	-0.14	-0.79	0.95	0.48	-0.49	-0.17	-0.43	0.00	0.80	0.61
TCGA-CV-59 79	-0.44	-0.13	-0.70	-1.00	-0.25	2.65	-0.93	-1.03	-0.85	-0.40	0.23	2.24	1.13	-0.83	-0.20	0.05	1.71	0.09	0.63	-0.49	-0.17	-0.43	0.01	0.24	0.99
TCGA-CV-60 03	-0.24	-0.13	-0.56	-0.70	7.08	0.40	-0.93	-1.03	-0.77	-0.40	-0.79	0.71	-0.27	0.92	0.33	-0.31	-0.39	-0.16	-0.92	-0.04	-0.17	-0.43	0.00	0.52	0.86
TCGA-CV-64 33	-0.50	-0.06	-0.18	4.21	-0.25	-1.13	1.18	0.42	0.48	-0.40	-0.79	-0.40	-0.27	-0.68	-0.82	-0.56	-0.79	0.46	-0.56	-0.49	-0.17	-0.43	0.01	0.24	1.01
TCGA-CV-64 36	-0.15	-0.13	-0.46	3.04	-0.25	-1.13	2.05	0.23	-0.56	0.69	-0.79	1.19	-0.27	-1.16	0.56	0.68	-0.76	-0.77	-0.04	-0.49	-0.17	-0.26	0.00	0.31	0.97
TCGA-CV-64 41	-0.50	-0.13	-0.86	-1.00	-0.25	-0.45	-0.93	-0.77	-0.57	-0.21	-0.79	1.61	-0.27	-1.01	1.32	-0.33	-0.64	7.69	2.87	-0.49	-0.17	-0.43	0.00	0.37	0.98
TCGA-CV-69 33	-0.50	-0.13	-0.82	-0.92	0.25	-0.91	-0.93	-1.03	-0.45	-0.40	-0.22	-0.80	-0.27	3.60	-1.27	-0.76	-0.70	0.12	-0.86	-0.49	-0.17	-0.43	0.00	0.81	0.58
TCGA-CV-69 34	-0.41	-0.13	-0.86	0.80	-0.25	-0.14	0.00	-0.94	0.13	-0.40	0.45	-0.61	-0.27	0.45	0.84	0.37	-0.70	-0.77	0.63	-0.49	-0.17	-0.43	0.00	0.64	0.78
TCGA-CV-69 35	0.42	-0.13	-0.65	-0.94	-0.25	-0.33	-0.93	-0.38	-0.44	-0.29	-0.79	-0.67	-0.27	1.93	-0.85	1.52	-0.48	-0.77	0.01	-0.49	2.23	-0.43	0.00	0.47	0.90
TCGA-CV-69 36	-0.25	-0.13	0.66	2.05	-0.25	-1.13	0.01	1.95	-0.37	-0.40	-0.69	0.33	-0.27	-0.69	0.75	-0.49	-0.37	-0.63	0.56	-0.49	-0.17	-0.43	0.00	0.37	0.93
TCGA-CV-69 37	-0.50	-0.13	-0.86	-0.81	-0.25	0.39	-0.93	0.69	0.86	-0.40	0.54	-0.80	-0.27	2.12	-1.08	-0.03	-0.79	-0.60	-0.92	-0.49	-0.17	-0.43	0.00	0.63	0.77
TCGA-CV-69 38	-0.26	-0.13	-0.86	0.72	-0.25	-1.13	-0.43	0.89	-0.09	-0.40	-0.79	0.79	-0.27	0.00	0.39	0.64	-0.41	1.75	0.59	-0.49	-0.17	-0.37	0.00	0.36	0.94
TCGA-CV-69 39	-0.39	-0.13	-0.21	-0.05	-0.25	1.37	0.46	0.53	-0.70	-0.40	-0.47	-0.63	-0.27	0.33	0.57	-0.64	-0.35	-0.77	-0.46	-0.36	-0.17	-0.43	0.00	0.42	0.91
TCGA-CV-69 40	-0.50	-0.13	-0.21	-1.00	-0.25	0.50	-0.93	0.58	0.20	-0.40	-0.79	0.38	-0.27	0.99	-0.88	0.01	1.20	-0.77	-0.05	-0.49	-0.17	-0.43	0.01	0.28	0.99
TCGA-CV-69 41	-0.01	-0.13	0.20	-0.69	-0.25	0.85	-0.38	-1.03	-0.22	-0.40	0.45	-0.80	-0.27	-0.18	0.42	1.52	-0.20	-0.09	0.07	-0.43	-0.17	-0.24	0.00	0.49	0.87
TCGA-CV-69 42	-0.45	-0.13	1.09	0.73	-0.25	-0.93	0.66	-0.70	-0.65	-0.40	2.78	-0.69	-0.27	-0.37	0.48	0.21	-0.73	-0.42	0.70	-0.49	-0.17	-0.25	0.00	0.54	0.85
TCGA-CV-69 43	-0.08	-0.13	-0.28	2.62	-0.25	-1.00	-0.06	0.82	0.18	-0.40	-0.79	0.23	-0.27	-1.16	2.00	-0.29	-0.59	-0.77	1.58	-0.49	-0.17	-0.43	0.00	0.55	0.83
TCGA-CV-69 45	-0.50	-0.13	-0.11	-0.76	5.89	0.09	-0.77	-1.03	-0.85	-0.40	-0.15	-0.80	-0.27	1.81	-1.43	-0.41	-0.79	0.69	-0.92	0.14	-0.17	-0.43	0.06	0.13	1.10
TCGA-CV-69 48	0.11	-0.13	-0.36	-0.24	-0.25	-0.66	0.62	-0.51	0.79	-0.40	0.53	0.42	-0.27	0.44	-0.03	1.16	-0.28	-0.77	-0.16	-0.49	-0.17	-0.43	0.00	0.49	0.87
TCGA-CV-69 50	-0.50	-0.13	-0.86	-0.61	-0.25	-0.50	-0.74	0.92	-0.50	-0.40	-0.79	1.04	-0.27	1.34	1.06	0.37	-0.67	-0.77	-0.92	0.20	-0.17	-0.43	0.00	0.47	0.90
TCGA-CV-69	-0.48	-0.13	0.30	-0.45	-0.25	0.11	-0.93	1.37	-0.16	-0.40	-0.79	2.49	-0.27	-0.75	-0.79	0.13	1.04	-0.31	3.53	-0.49	-0.17	-0.43	0.10	0.10	1.04

51																									
TCGA-CV-69 52	0.50	-0.13	0.05	-0.04	-0.25	0.39	0.10	-0.33	-0.43	-0.40	-0.75	0.53	-0.27	-0.16	0.39	0.47	-0.56	0.71	-0.92	0.01	-0.17	0.01	0.00	0.38	0.93
TCGA-CV-69 53	-0.37	-0.13	3.15	0.80	-0.25	-1.13	1.28	-0.43	0.08	-0.40	0.00	-0.80	0.40	-0.47	-0.97	0.10	-0.50	-0.37	-0.92	-0.27	-0.17	0.24	0.03	0.18	1.00
TCGA-CV-69 54	-0.50	-0.13	-0.54	-0.91	-0.25	-1.13	0.57	0.46	0.37	-0.09	-0.79	-0.70	-0.27	-0.90	3.63	-0.36	2.48	-0.64	1.56	-0.49	-0.17	-0.10	0.00	0.51	0.87
TCGA-CV-69 55	-0.29	-0.13	-0.86	0.47	-0.25	-0.30	-0.92	1.22	1.25	-0.40	-0.79	1.89	-0.27	-0.51	0.10	-0.62	1.36	-0.05	1.35	-0.49	-0.17	0.08	0.02	0.20	1.01
TCGA-CV-69 56	-0.50	-0.13	-0.43	-0.96	-0.25	-1.13	-0.93	1.06	0.67	-0.40	0.08	-0.80	-0.27	2.93	-0.76	-0.56	-0.79	-0.77	-0.92	-0.49	1.00	-0.43	0.02	0.19	1.13
TCGA-CV-69 59	-0.13	-0.13	-0.02	-1.00	-0.25	1.76	-0.03	-1.03	-0.85	-0.40	1.34	-0.80	-0.27	0.34	0.05	-0.21	-0.79	-0.06	-0.92	0.83	-0.17	0.77	0.00	0.33	0.95
TCGA-CV-69 60	-0.50	-0.13	-0.64	-0.85	0.20	-0.74	-0.54	-0.11	0.28	-0.40	0.99	-0.80	-0.27	1.20	-1.43	-1.00	0.97	-0.42	-0.92	3.23	10.29	-0.34	0.15	0.09	1.11
TCGA-CV-69 61	-0.50	-0.13	-0.83	-1.00	-0.25	-0.26	-0.13	-0.29	-0.72	-0.40	1.53	-0.80	1.84	-0.06	2.29	-0.41	-0.79	2.62	-0.92	-0.20	-0.17	3.12	0.00	0.37	0.97
TCGA-CV-69 62	-0.28	-0.13	-0.62	-0.95	0.42	-1.13	-0.93	-0.44	0.05	-0.40	0.79	-0.80	-0.27	2.57	-1.21	1.07	-0.78	-0.77	-0.92	0.14	-0.17	-0.43	0.00	0.34	1.02
TCGA-CV-70 89	-0.50	-0.13	-0.86	-0.32	-0.25	1.01	-0.81	-1.03	0.19	-0.40	-0.79	1.63	-0.27	-0.20	0.93	0.01	0.70	0.37	0.21	-0.49	-0.17	-0.06	0.00	0.32	0.96
TCGA-CV-70 90	-0.12	-0.13	-0.86	-1.00	-0.25	-0.31	-0.69	0.23	-0.51	1.35	-0.20	-0.40	-0.27	0.77	0.74	1.98	-0.40	-0.69	0.48	-0.49	-0.17	-0.43	0.00	0.60	0.80
TCGA-CV-70 91	0.29	-0.13	-0.67	0.45	-0.25	-0.27	1.05	2.23	-0.62	-0.40	-0.41	-0.80	-0.27	-0.21	1.01	-0.04	0.24	-0.77	-0.10	-0.49	-0.17	-0.16	0.00	0.45	0.89
TCGA-CV-70 95	-0.38	-0.13	0.32	-0.75	-0.25	1.68	-0.93	-1.03	-0.85	-0.40	-0.79	0.45	-0.27	-0.26	0.30	0.57	0.98	0.08	-0.43	-0.49	-0.17	-0.43	0.00	0.37	0.93
TCGA-CV-70 97	-0.27	-0.13	-0.86	-1.00	-0.25	2.13	-0.92	-1.03	-0.85	-0.40	2.17	-0.70	-0.27	0.73	-0.52	1.53	-0.79	-0.17	-0.92	-0.49	-0.17	-0.43	0.00	0.63	0.78
TCGA-CV-70 99	-0.45	-0.13	-0.57	0.87	-0.25	-0.49	-0.20	-0.10	0.24	-0.40	0.26	0.30	-0.27	-0.04	1.23	-0.16	-0.79	0.58	0.13	-0.49	-0.17	0.17	0.00	0.43	0.91
TCGA-CV-71 00	-0.17	-0.13	-0.73	1.13	-0.25	-0.40	0.75	0.76	-0.38	-0.39	0.60	-0.58	-0.12	-0.89	2.54	0.12	-0.28	-0.77	0.45	-0.49	-0.17	-0.18	0.00	0.41	0.92
TCGA-CV-71 01	-0.48	-0.13	2.14	-0.38	-0.25	-0.80	-0.56	-0.15	-0.58	0.51	-0.08	-0.67	-0.27	0.34	0.40	0.29	-0.66	-0.77	0.70	-0.49	-0.17	-0.32	0.00	0.50	0.87
TCGA-CV-71 02	-0.29	-0.13	-0.80	-0.53	-0.25	0.83	-0.25	-1.03	-0.52	-0.40	0.57	-0.80	-0.27	0.65	0.53	1.40	-0.52	-0.77	-0.10	-0.49	-0.17	-0.23	0.00	0.68	0.75
TCGA-CV-71 03	-0.27	-0.13	-0.78	-0.87	-0.25	0.07	-0.93	-1.03	1.66	-0.40	-0.79	0.59	-0.27	0.99	-0.84	0.33	0.65	0.02	0.97	-0.49	-0.17	-0.43	0.00	0.40	0.93
TCGA-CV-71 04	-0.19	-0.13	-0.64	-0.99	-0.25	2.45	-0.92	-1.03	0.07	-0.40	0.09	-0.30	0.15	-0.23	-0.27	0.26	0.46	0.91	0.48	-0.49	-0.17	-0.17	0.00	0.37	0.93
TCGA-CV-71	-0.50	-0.13	-0.42	-1.00	-0.25	-0.26	-0.93	-0.57	0.26	-0.40	-0.79	-0.76	-0.27	1.56	-1.23	-0.40	2.03	-0.59	0.97	-0.49	-0.17	-0.40	0.00	0.62	0.78

77																									
TCGA-CV-71 78	-0.47	-0.13	-0.85	0.55	-0.25	0.39	-0.40	0.84	-0.14	-0.40	0.26	-0.20	-0.27	0.22	0.48	-0.46	0.10	-0.77	0.76	-0.49	-0.17	-0.43	0.00	0.58	0.82
TCGA-CV-71 80	-0.16	-0.13	1.10	0.80	-0.25	-0.86	1.64	2.29	-0.85	-0.40	-0.79	-0.10	-0.27	-0.41	-0.35	-1.13	0.56	-0.77	0.75	-0.49	-0.17	0.07	0.12	0.10	1.03
TCGA-CV-71 83	0.25	-0.13	-0.30	-0.24	-0.17	-0.59	-0.91	-0.95	-0.42	-0.40	0.86	-0.80	-0.06	1.45	-0.94	-0.81	0.93	-0.06	0.33	-0.49	-0.17	0.28	0.24	0.06	1.12
TCGA-CV-72 35	0.30	-0.13	-0.86	0.00	-0.25	-0.87	0.44	1.27	0.20	-0.40	0.98	1.09	0.08	-0.93	1.29	-0.38	0.13	1.83	1.44	-0.49	-0.17	-0.43	0.00	0.48	0.88
TCGA-CV-72 36	-0.46	-0.13	-0.70	-0.72	-0.25	1.63	-0.93	-1.03	-0.85	-0.40	-0.79	0.38	-0.27	-0.12	1.83	-0.31	0.82	-0.77	-0.92	1.32	-0.17	-0.43	0.00	0.30	0.98
TCGA-CV-72 38	-0.22	-0.13	-0.21	0.13	-0.25	-0.20	-0.64	0.19	1.47	0.17	-0.79	-0.80	-0.27	0.31	1.74	-0.60	-0.22	-0.75	0.15	-0.49	-0.17	-0.43	0.00	0.49	0.87
TCGA-CV-72 42	-0.08	-0.13	1.59	1.37	-0.25	-0.73	1.29	-0.47	-0.28	1.58	-0.59	-0.80	-0.27	-0.93	0.74	-0.64	0.08	-0.53	0.26	-0.49	-0.17	-0.35	0.00	0.51	0.87
TCGA-CV-72 45	-0.39	-0.13	-0.11	-0.87	-0.25	1.43	-0.33	-0.67	-0.85	-0.40	1.65	-0.04	-0.27	0.94	-1.41	-0.23	-0.60	0.88	-0.92	0.17	-0.17	-0.43	0.02	0.20	1.03
TCGA-CV-72 47	-0.50	-0.13	-0.73	-1.00	2.50	1.34	-0.93	-1.03	-0.85	-0.40	1.40	-0.80	-0.27	2.12	-0.56	-1.36	-0.79	-0.77	-0.92	0.19	-0.17	-0.43	0.01	0.25	1.04
TCGA-CV-72 48	0.24	-0.13	1.75	0.29	-0.25	-0.54	0.15	-0.61	0.83	0.18	-0.79	-0.34	-0.27	0.11	-0.53	0.17	-0.68	-0.77	0.41	-0.49	-0.17	-0.24	0.00	0.55	0.85
TCGA-CV-72 50	-0.50	-0.13	-0.08	-0.39	-0.25	-0.13	1.38	1.41	-0.68	-0.40	0.95	-0.80	-0.27	0.30	-0.04	-0.16	-0.79	-0.11	-0.92	1.04	-0.17	0.23	0.06	0.13	1.04
TCGA-CV-72 52	-0.50	-0.13	0.36	-0.28	-0.25	0.29	-0.85	-0.56	-0.12	-0.40	-0.22	0.57	-0.27	-0.01	0.10	-0.28	1.51	-0.12	-0.92	0.06	-0.07	-0.22	0.00	0.30	0.97
TCGA-CV-72 53	0.51	-0.13	1.29	-0.96	-0.25	0.81	-0.93	-1.03	-0.37	-0.40	-0.79	1.29	-0.27	0.39	-1.43	-0.79	1.13	0.33	0.23	-0.49	-0.17	-0.43	0.13	0.10	1.05
TCGA-CV-72 54	2.16	-0.13	-0.42	-0.26	-0.25	0.09	-0.59	0.55	0.37	-0.40	-0.79	0.81	-0.27	-0.30	-0.21	2.15	-0.25	-0.77	0.04	-0.49	-0.17	-0.43	0.00	0.55	0.84
TCGA-CV-72 55	0.29	-0.13	-0.48	-1.00	-0.25	-0.34	-0.93	-0.59	0.38	-0.31	-0.79	0.32	-0.27	2.97	-0.84	-1.71	-0.79	-0.77	0.38	-0.49	-0.17	-0.43	0.00	0.69	0.73
TCGA-CV-72 61	1.38	-0.13	0.07	-0.86	-0.25	-1.13	0.14	-0.35	-0.78	1.97	-0.79	-0.80	-0.27	1.25	0.69	0.09	-0.72	-0.13	-0.26	-0.48	-0.17	0.59	0.00	0.30	1.00
TCGA-CV-72 63	-0.10	-0.13	-0.48	-0.69	-0.25	-0.11	-0.57	-1.03	0.14	-0.22	-0.79	0.71	-0.27	1.11	0.01	-0.15	0.31	-0.77	1.33	-0.49	-0.17	-0.43	0.00	0.71	0.72
TCGA-CV-74 06	-0.50	-0.13	-0.65	-1.00	-0.25	-0.56	-0.93	-0.19	-0.07	-0.40	3.39	-0.80	-0.27	1.54	-1.24	0.61	0.08	-0.70	-0.92	1.02	-0.17	-0.43	0.01	0.25	1.03
TCGA-CV-74 07	-0.50	-0.13	-0.86	-0.17	-0.25	-0.02	1.15	0.22	1.06	-0.40	-0.13	1.40	5.56	-1.20	-0.20	-0.18	1.58	-0.58	2.56	-0.49	-0.17	-0.43	0.00	0.32	0.95
TCGA-CV-74 10	9.18	-0.13	-0.30	-0.06	-0.25	0.14	0.49	0.31	2.65	-0.40	-0.04	-0.80	-0.27	-0.81	-0.39	-1.15	-0.24	-0.76	-0.16	-0.49	-0.17	-0.40	0.00	0.59	0.82
TCGA-CV-74	-0.37	-0.13	0.85	-0.92	-0.25	0.27	-0.38	0.02	0.07	2.92	-0.79	-0.80	-0.27	0.47	0.00	-0.26	-0.26	0.75	-0.57	-0.49	-0.17	-0.05	0.05	0.14	1.04

11

TCGA-CV-74 13	-0.50	-0.13	-0.86	-0.49	-0.25	1.01	-0.93	0.21	0.70	-0.40	-0.79	1.04	-0.27	0.10	0.03	0.46	0.47	-0.45	0.23	-0.49	-0.17	0.57	0.00	0.44	0.90
TCGA-CV-74 15	-0.47	-0.13	-0.13	0.80	-0.25	0.35	-0.01	-1.03	-0.21	-0.40	-0.65	0.49	-0.27	-0.13	1.28	-0.24	-0.33	-0.77	0.64	-0.49	-0.17	-0.43	0.00	0.47	0.88
TCGA-CV-74 16	-0.50	-0.13	-0.81	-0.85	-0.25	1.41	-0.93	-1.03	-0.85	-0.40	0.94	-0.80	1.60	0.11	-1.43	2.29	-0.25	2.55	-0.92	-0.10	-0.17	-0.43	0.00	0.33	0.96
TCGA-CV-74 18	-0.02	-0.13	-0.03	0.22	-0.25	-0.66	1.30	0.84	0.17	-0.40	1.92	-0.55	-0.27	-0.43	0.27	0.22	-0.47	-0.19	1.16	-0.49	-0.17	-0.43	0.00	0.31	0.96
TCGA-CV-74 22	1.47	-0.13	-0.82	-1.00	-0.25	0.84	-0.44	0.38	-0.37	0.36	-0.79	0.11	-0.27	0.66	0.93	0.09	-0.67	0.00	-0.49	-0.49	-0.17	-0.42	0.10	0.11	1.08
TCGA-CV-74 23	-0.32	-0.13	-0.78	-1.00	3.10	0.75	-0.93	-1.03	-0.85	-0.40	2.08	-0.80	-0.27	1.81	-1.06	-1.62	-0.36	0.69	-0.92	0.73	-0.17	-0.43	0.00	0.42	0.92
TCGA-CV-74 24	1.90	-0.13	1.44	0.39	-0.25	0.20	0.77	-1.03	0.05	1.29	-0.79	0.03	-0.27	-0.36	-0.29	-0.48	-0.79	-0.77	0.62	-0.49	-0.17	-0.43	0.00	0.40	0.92
TCGA-CV-74 25	0.49	-0.13	-0.30	0.69	-0.25	-0.91	-0.07	0.89	1.05	-0.40	-0.79	1.31	-0.26	-0.86	-0.24	-0.38	2.03	-0.03	0.96	-0.49	-0.17	-0.08	0.01	0.27	0.97
TCGA-CV-74 27	0.09	-0.13	-0.86	0.73	-0.25	0.49	1.17	0.64	-0.67	-0.40	-0.44	0.32	-0.27	-0.92	0.78	-0.28	0.71	-0.43	0.86	-0.49	-0.17	0.52	0.00	0.32	0.96
TCGA-CV-74 28	-0.24	-0.13	-0.56	0.31	-0.25	-0.78	0.00	0.60	0.08	-0.40	-0.79	1.48	-0.27	0.37	0.47	0.59	-0.10	-0.77	0.03	-0.49	-0.17	-0.36	0.00	0.33	0.96
TCGA-CV-74 29	-0.35	-0.13	-0.86	-0.04	-0.25	1.25	0.01	-1.03	-0.85	-0.40	0.21	-0.69	-0.27	0.32	-0.42	2.82	-0.79	-0.77	-0.75	-0.49	-0.17	-0.30	0.00	0.29	0.98
TCGA-CV-74 30	-0.50	-0.13	-0.65	-0.97	-0.25	4.22	-0.93	-1.03	-0.85	-0.40	0.54	-0.71	-0.14	0.20	-1.13	-0.65	0.23	1.04	-0.79	-0.49	-0.17	-0.43	0.20	0.07	1.08
TCGA-CV-74 32	-0.50	-0.13	-0.58	-0.40	1.47	0.66	1.73	-1.03	-0.85	-0.40	1.64	-0.80	0.12	-0.18	0.76	0.06	-0.27	-0.04	0.47	-0.49	-0.17	-0.43	0.02	0.21	1.01
TCGA-CV-74 33	-0.50	-0.13	-0.86	0.98	-0.25	0.58	0.84	-0.30	-0.23	-0.40	1.03	-0.80	-0.27	-0.39	0.63	0.46	-0.70	0.23	-0.16	-0.49	-0.17	-0.31	0.00	0.42	0.91
TCGA-CV-74 34	-0.50	-0.13	-0.86	-0.56	-0.25	2.26	1.10	-1.03	-0.85	-0.40	0.52	1.79	0.48	-1.01	1.57	1.00	-0.40	-0.77	-0.30	-0.49	0.13	-0.43	0.00	0.31	0.97
TCGA-CV-74 35	-0.34	-0.13	0.09	-0.57	-0.25	-0.04	-0.12	-0.97	0.02	-0.40	-0.19	-0.80	-0.27	1.07	-0.20	0.54	-0.79	0.12	0.35	-0.49	-0.17	-0.31	0.00	0.72	0.72
TCGA-CV-74 37	-0.43	-0.13	-0.33	-0.72	-0.25	0.32	-0.93	0.46	-0.28	-0.40	-0.79	1.52	-0.27	-1.07	-0.44	-0.44	4.51	0.48	1.12	-0.49	-0.17	-0.27	0.03	0.18	1.02
TCGA-CV-74 38	-0.45	-0.13	0.88	-1.00	-0.25	0.27	-0.90	0.23	0.17	-0.40	0.02	-0.14	-0.27	0.34	1.59	-0.45	0.28	-0.77	-0.78	-0.49	-0.17	-0.43	0.00	0.53	0.85
TCGA-CV-74 40	-0.50	-0.13	0.02	-1.00	-0.25	-0.52	-0.93	-0.53	-0.38	-0.30	-0.79	-0.37	-0.27	0.84	-0.41	-0.65	-0.79	0.55	-0.92	5.26	1.75	-0.06	0.00	0.61	0.80
TCGA-CV-74 46	0.84	-0.13	-0.51	-0.29	-0.25	-0.55	1.53	-0.23	-0.85	0.52	-0.44	-0.80	-0.27	0.35	0.58	0.91	-0.01	-0.15	-0.88	-0.22	-0.17	-0.43	0.04	0.16	1.05
TCGA-CV-75	-0.37	-0.13	-0.36	-1.00	-0.25	0.96	-0.77	0.83	-0.83	2.15	-0.79	-0.02	-0.27	0.25	0.39	0.70	0.33	-0.77	0.38	-0.49	-0.17	-0.36	0.01	0.25	1.00

68																									
TCGA-CV-A4 5O	-0.50	-0.13	-0.80	-0.48	-0.25	-0.01	-0.45	2.52	-0.85	-0.40	-0.79	1.34	-0.27	-0.77	1.01	0.12	1.04	-0.53	1.45	-0.49	-0.17	3.04	0.39	0.04	1.08
TCGA-CV-A4 5P	0.03	-0.13	-0.07	-0.04	-0.25	-1.13	0.03	1.67	-0.58	-0.40	-0.35	-0.35	-0.27	1.12	-0.35	-0.93	-0.54	0.91	-0.92	0.28	-0.17	0.72	0.03	0.17	1.05
TCGA-CV-A4 5Q	-0.41	-0.13	-0.86	0.46	-0.25	-1.13	0.54	1.08	-0.84	-0.40	2.28	0.32	-0.27	0.28	-0.16	0.37	0.14	-0.46	0.21	-0.49	-0.17	0.54	0.07	0.12	1.06
TCGA-CV-A4 5R	-0.40	-0.13	-0.86	-0.17	-0.25	0.27	-0.40	1.00	0.26	-0.40	-0.08	-0.19	-0.27	0.11	0.93	0.44	-0.79	-0.23	1.41	-0.49	-0.17	-0.19	0.00	0.38	0.93
TCGA-CV-A4 5T	-0.39	3.51	-0.04	0.17	-0.25	-0.51	0.09	1.62	2.26	4.29	-0.79	0.77	-0.27	-0.66	1.08	-0.48	-0.44	-0.57	-0.39	-0.49	-0.17	-0.43	0.00	0.50	0.86
TCGA-CV-A4 5U	-0.45	-0.13	0.19	1.57	-0.25	1.12	1.26	2.59	-0.75	-0.40	-0.79	0.39	0.44	-0.90	-0.64	-1.16	-0.07	-0.61	0.01	-0.49	-0.17	-0.43	0.02	0.20	1.01
TCGA-CV-A4 5V	-0.34	-0.13	-0.47	0.23	-0.25	-0.79	0.38	2.07	-0.73	2.18	-0.61	0.39	-0.27	-0.34	1.66	0.02	-0.62	-0.45	1.22	-0.49	-0.17	0.19	0.03	0.18	1.04
TCGA-CV-A4 5W	0.39	-0.13	2.54	0.23	-0.25	-0.89	1.02	-0.64	-0.58	-0.40	0.28	-0.80	-0.27	0.13	-0.12	-0.21	-0.79	-0.77	-0.26	-0.49	-0.17	-0.43	0.00	0.62	0.81
TCGA-CV-A4 5X	-0.50	-0.13	-0.73	-1.00	-0.25	-0.78	-0.03	1.53	0.20	-0.40	0.49	-0.80	-0.27	2.41	-0.86	-1.66	0.83	-0.64	-0.92	-0.49	0.97	-0.43	0.01	0.25	1.06
TCGA-CV-A4 5Y	-0.50	0.02	0.84	-0.18	-0.25	0.02	0.04	-0.03	1.19	-0.40	-0.79	-0.52	-0.27	0.28	-0.39	-0.70	-0.16	0.57	0.42	-0.49	-0.17	-0.43	0.00	0.61	0.81
TCGA-CV-A4 5Z	0.21	-0.13	-0.78	0.00	-0.25	0.10	1.69	0.36	-0.57	-0.40	-0.76	0.00	-0.27	-0.03	0.41	0.42	-0.32	0.50	0.04	-0.49	-0.17	-0.43	0.00	0.48	0.87
TCGA-CV-A4 60	-0.31	-0.13	0.19	0.12	-0.25	0.70	-0.17	0.74	1.34	-0.40	0.26	-0.68	-0.27	-0.33	0.68	-0.32	-0.23	-0.53	-0.88	0.04	-0.17	-0.43	0.02	0.21	1.01
TCGA-CV-A4 61	-0.50	-0.13	-0.45	1.95	-0.25	-1.13	0.70	-0.25	2.03	-0.40	-0.79	0.99	-0.27	-0.75	0.42	-0.36	0.03	-0.50	1.67	-0.49	-0.17	-0.43	0.00	0.48	0.87
TCGA-CV-A4 64	-0.10	-0.13	-0.86	-1.00	-0.25	0.18	-0.93	0.83	-0.43	1.89	-0.79	1.18	-0.27	-0.32	2.57	-0.05	0.40	-0.53	1.60	-0.49	-0.17	-0.43	0.00	0.52	0.86
TCGA-CV-A4 65	0.09	-0.13	2.12	-1.00	-0.25	0.00	-0.93	0.51	-0.42	-0.40	-0.29	-0.80	-0.27	0.26	0.28	-1.08	0.60	-0.19	-0.44	-0.04	-0.17	-0.12	0.56	0.01	1.09
TCGA-CV-A4 68	-0.33	-0.13	-0.01	1.66	-0.25	-0.75	0.10	1.31	1.01	-0.38	-0.78	-0.44	-0.27	-0.86	0.43	-0.02	-0.30	0.48	1.04	-0.49	-0.17	-0.43	0.00	0.47	0.88
TCGA-CV-A6 JD	-0.50	3.54	-0.74	0.76	-0.25	-0.92	-0.32	1.05	0.38	-0.40	-0.79	0.87	-0.27	-0.99	0.84	0.83	2.34	-0.70	-0.92	-0.25	-0.17	-0.03	0.01	0.25	0.99
TCGA-CV-A6 JE	-0.50	-0.13	-0.39	2.27	-0.25	-1.13	-0.92	0.43	1.55	-0.40	-0.12	3.47	0.60	-0.64	-0.47	-0.07	0.52	-0.34	-0.92	-0.43	-0.17	-0.43	0.00	0.49	0.87
TCGA-CV-A6 JM	-0.50	-0.13	0.97	-0.50	-0.25	-1.13	-0.76	-0.40	-0.85	-0.40	-0.79	-0.06	-0.27	1.50	-1.40	1.02	-0.79	1.16	-0.10	-0.49	-0.17	-0.43	0.00	0.48	0.88
TCGA-CV-A6 JN	-0.04	-0.13	-0.50	0.51	-0.25	-0.92	-0.59	2.01	1.24	-0.40	-0.79	1.98	-0.04	-0.87	0.22	0.11	0.41	0.06	-0.92	1.48	-0.17	1.09	0.00	0.34	0.95
TCGA-CV-A6	0.05	-0.13	-0.86	1.56	-0.25	-1.13	-0.02	2.67	0.06	-0.40	-0.41	0.81	-0.27	-0.76	2.04	0.40	-0.66	-0.63	-0.92	0.36	-0.17	-0.43	0.00	0.33	0.97

JO																									
TCGA-CV-A6JT	-0.50	3.55	-0.86	0.35	-0.25	-1.13	-0.92	1.79	0.89	-0.40	-0.79	2.65	-0.27	-0.75	-0.17	-0.06	2.73	-0.42	0.70	-0.49	-0.17	-0.26	0.00	0.39	0.92
TCGA-CV-A6JU	-0.50	-0.13	-0.82	-0.58	-0.25	-1.13	-0.93	0.72	4.68	-0.40	0.27	-0.80	-0.21	-0.59	0.38	2.80	-0.01	1.20	-0.41	-0.49	-0.17	-0.42	0.01	0.26	0.99
TCGA-CV-A6JY	0.34	-0.13	2.96	-0.93	0.40	-0.29	-0.68	-1.03	-0.24	-0.40	0.25	-0.80	-0.27	-0.14	-1.28	0.04	0.28	0.35	0.20	0.06	-0.17	0.80	0.40	0.04	1.06
TCGA-CV-A6JZ	-0.42	-0.13	-0.55	2.74	-0.25	-1.13	2.28	-0.06	0.28	-0.40	0.14	-0.80	-0.27	-0.97	0.00	1.50	-0.45	-0.60	-0.41	-0.49	-0.17	-0.43	0.00	0.44	0.90
TCGA-CV-A6K0	0.23	-0.13	-0.86	0.03	-0.25	0.13	-0.85	0.47	1.98	1.49	-0.79	0.11	-0.27	-0.49	0.65	0.92	-0.15	0.84	-0.22	-0.49	-0.17	-0.43	0.00	0.52	0.86
TCGA-CV-A6K1	-0.50	-0.13	0.42	0.97	-0.25	-0.91	-0.18	-0.04	1.37	-0.40	1.66	1.00	0.04	-0.56	-0.15	0.90	-0.66	0.35	-0.74	-0.49	-0.17	-0.38	0.01	0.28	0.98
TCGA-CV-A6K2	-0.50	-0.13	-0.81	0.19	-0.25	0.62	-0.93	-0.51	-0.85	-0.40	-0.79	1.65	-0.27	0.35	0.01	0.39	-0.36	0.42	-0.92	0.42	-0.17	0.73	0.02	0.19	1.04
TCGA-CX-7085	0.17	-0.13	0.17	0.07	-0.25	1.21	0.86	-1.03	0.40	-0.40	0.84	-0.80	1.16	-0.51	0.62	-0.62	-0.77	-0.39	-0.59	-0.09	-0.17	1.78	0.00	0.46	0.89
TCGA-CX-7086	0.28	-0.13	0.72	-0.90	-0.25	0.59	1.28	0.01	-0.05	-0.40	0.47	-0.80	-0.27	-0.81	0.56	-0.44	0.49	0.64	-0.92	0.05	-0.17	1.71	0.18	0.08	1.05
TCGA-CX-7219	-0.50	-0.13	-0.39	-0.55	-0.25	0.52	1.25	-1.03	-0.25	-0.40	1.16	-0.80	-0.27	0.90	0.12	-0.38	-0.78	-0.34	-0.92	0.16	-0.17	0.35	0.00	0.41	0.92
TCGA-CX-A4AQ	6.57	-0.13	0.82	-0.37	-0.25	0.02	-0.10	-0.20	3.40	0.22	-0.79	0.11	0.28	-0.90	-0.61	-1.17	0.21	-0.63	0.22	-0.49	-0.17	-0.43	0.03	0.18	1.01
TCGA-D6-6515	-0.33	-0.13	-0.71	0.29	-0.25	0.09	-0.83	0.16	1.70	-0.40	-0.79	1.09	-0.27	-1.24	1.56	-0.22	2.16	-0.72	1.02	-0.49	-0.17	0.11	0.00	0.42	0.91
TCGA-D6-6516	0.86	-0.13	-0.60	0.96	-0.25	-1.13	-0.01	0.98	-0.14	0.99	-0.74	1.45	-0.27	-0.95	0.78	-0.13	0.22	0.73	1.95	-0.49	-0.17	-0.26	0.00	0.43	0.91
TCGA-D6-6517	0.53	-0.13	0.43	-0.63	-0.25	0.41	-0.93	-1.03	2.10	-0.40	-0.62	-0.35	1.25	-0.21	-1.36	0.31	1.05	1.90	-0.92	-0.28	-0.17	-0.20	0.00	0.31	0.96
TCGA-D6-6823	-0.28	-0.13	-0.15	-1.00	1.62	-1.12	1.02	-0.15	-0.43	-0.18	-0.24	-0.80	-0.27	1.24	0.33	-0.38	-0.77	-0.05	-0.63	-0.37	-0.17	4.17	0.01	0.25	1.02
TCGA-D6-6824	-0.04	-0.13	-0.36	-0.18	-0.25	0.08	0.28	-0.60	0.19	-0.40	0.97	-0.80	-0.27	-0.35	1.21	0.91	-0.54	-0.77	1.32	-0.49	-0.17	0.82	0.00	0.53	0.85
TCGA-D6-6825	-0.32	-0.13	-0.68	0.77	-0.25	-0.37	2.88	1.15	-0.80	-0.40	-0.43	-0.46	-0.27	-0.81	0.01	0.90	-0.30	-0.01	1.30	-0.49	-0.17	0.18	0.00	0.37	0.93
TCGA-D6-6826	0.32	-0.13	-0.53	-1.00	-0.25	0.32	-0.87	-1.03	-0.45	0.45	0.09	-0.80	-0.27	0.61	-0.23	0.87	-0.79	-0.75	-0.92	2.39	-0.17	3.38	0.01	0.28	0.99
TCGA-D6-6827	1.17	-0.13	-0.82	-0.58	-0.25	0.37	-0.76	-0.39	1.41	-0.40	-0.79	0.01	-0.27	0.99	-0.12	0.24	-0.56	-0.77	0.50	-0.49	-0.17	-0.40	0.02	0.21	1.03
TCGA-D6-8568	-0.42	-0.13	-0.41	-1.00	0.89	0.31	-0.93	-1.03	-0.85	-0.40	0.52	-0.80	0.15	-0.56	0.81	-0.59	-0.79	0.29	-0.92	5.56	-0.17	4.76	0.04	0.16	1.07
TCGA-D6-85	-0.50	-0.13	-0.77	-0.99	1.47	0.75	-0.62	-1.03	-0.58	-0.40	-0.79	1.92	-0.06	-0.20	1.22	-0.47	-0.14	2.49	0.95	-0.49	-0.17	-0.12	0.00	0.34	0.96

69																									
TCGA-D6-A4Z9	-0.26	-0.13	-0.52	-0.88	-0.25	1.07	-0.93	-1.03	-0.85	-0.40	-0.79	3.39	0.40	0.08	0.58	0.10	0.49	-0.06	-0.64	0.22	-0.17	-0.43	0.00	0.37	0.94
TCGA-D6-A4ZB	-0.50	-0.13	2.94	-1.00	-0.25	-0.46	-0.81	-0.96	-0.55	-0.18	-0.79	-0.16	-0.27	1.49	-0.75	-0.82	-0.79	-0.77	-0.92	0.12	-0.17	0.30	0.00	0.29	0.99
TCGA-D6-A6EK	-0.50	1.81	0.38	1.21	-0.25	-1.13	-0.36	0.94	3.25	-0.40	-0.79	1.23	-0.27	-1.05	-0.35	0.06	0.74	0.06	0.07	-0.49	-0.17	-0.43	0.00	0.32	0.95
TCGA-D6-A6EM	-0.19	-0.13	0.48	0.93	-0.25	-1.13	0.86	0.18	1.34	-0.08	-0.79	1.04	-0.27	-0.55	0.93	0.67	-0.79	-0.32	-0.67	-0.49	-0.17	-0.14	0.00	0.41	0.92
TCGA-D6-A6EN	-0.50	-0.13	-0.68	3.02	-0.25	-0.93	1.51	1.90	-0.11	4.21	-0.79	-0.04	-0.27	-1.34	-0.16	0.72	-0.59	-0.69	-0.92	0.54	-0.17	-0.39	0.00	0.43	0.91
TCGA-D6-A6EO	0.61	-0.13	1.20	-1.00	-0.25	-1.13	0.87	0.91	-0.51	-0.40	1.27	-0.80	-0.27	0.73	-0.50	-0.72	-0.79	1.25	-0.92	0.25	-0.17	0.23	0.09	0.12	1.05
TCGA-D6-A6EP	-0.50	-0.13	2.95	0.44	-0.25	-1.13	-0.16	-0.41	-0.85	1.78	-0.79	2.48	-0.27	-1.03	-1.38	2.61	-0.79	-0.77	0.84	-0.49	-0.17	-0.41	0.06	0.13	1.03
TCGA-D6-A6EQ	-0.50	-0.13	0.22	0.00	-0.25	-0.66	-0.46	0.93	1.01	-0.40	-0.79	1.50	-0.12	-0.55	0.50	-0.28	1.91	-0.77	0.23	-0.49	-0.17	-0.13	0.00	0.44	0.90
TCGA-D6-A6ES	-0.50	-0.13	-0.24	-0.34	-0.25	-0.66	1.02	-0.89	-0.41	-0.40	0.60	-0.80	1.39	0.67	-1.43	0.00	-0.23	2.64	-0.92	0.01	-0.17	1.59	0.00	0.33	0.96
TCGA-D6-A74Q	-0.50	-0.13	2.68	0.44	-0.25	-1.13	-0.62	3.18	0.08	-0.40	-0.79	1.08	-0.21	-0.64	-0.69	-1.04	-0.70	1.05	0.04	0.21	-0.17	-0.43	0.02	0.21	0.99
TCGA-DQ-5624	-0.50	-0.13	0.03	-0.77	-0.25	2.32	-0.93	-1.03	-0.77	-0.40	-0.55	-0.41	0.22	0.03	-0.92	-1.51	1.25	2.74	-0.09	-0.49	-0.17	-0.40	0.00	0.35	0.94
TCGA-DQ-5625	-0.31	-0.13	-0.86	2.85	-0.25	-1.13	-0.04	0.48	-0.22	-0.40	0.04	1.42	-0.12	-1.02	0.72	-0.26	0.76	-0.70	0.77	-0.49	-0.17	-0.43	0.00	0.33	0.96
TCGA-DQ-5629	2.37	-0.13	1.93	-1.00	2.27	-0.20	0.56	-1.03	-0.78	-0.14	-0.22	-0.80	-0.27	0.75	-0.76	-0.57	-0.74	-0.18	-0.74	-0.42	-0.17	-0.43	0.04	0.16	1.03
TCGA-DQ-5630	-0.39	-0.13	-0.45	-0.19	-0.25	-0.35	-0.82	0.95	-0.59	4.58	1.10	1.52	-0.27	-0.29	1.48	0.15	0.00	-0.77	-0.13	-0.49	-0.17	-0.43	0.00	0.45	0.89
TCGA-DQ-5631	-0.50	-0.13	1.25	-1.00	2.44	-0.39	-0.93	-0.22	-0.48	-0.40	0.98	-0.80	-0.27	0.23	-1.33	-0.32	1.71	0.51	-0.92	0.90	-0.17	-0.43	0.00	0.33	0.95
TCGA-DQ-7588	-0.50	-0.13	-0.83	-0.63	-0.25	2.20	0.11	-1.03	-0.85	-0.40	2.29	-0.80	0.12	1.37	-1.39	-1.71	-0.74	-0.77	-0.92	1.68	-0.17	-0.36	0.08	0.12	1.09
TCGA-DQ-7591	0.36	-0.07	-0.37	1.64	-0.25	-1.06	0.22	1.68	2.75	4.57	-0.79	1.37	-0.27	-1.11	0.44	-1.09	0.26	-0.22	-0.41	-0.49	-0.17	-0.43	0.05	0.14	1.05
TCGA-DQ-7592	-0.27	-0.13	-0.78	0.12	-0.25	0.49	0.47	-1.03	-0.85	-0.40	1.53	0.99	-0.02	0.13	0.86	0.15	-0.40	-0.73	0.06	-0.49	-0.17	-0.43	0.00	0.48	0.87
TCGA-F7-7848	-0.29	0.66	-0.02	0.45	-0.25	0.30	0.79	-0.61	0.43	-0.40	0.74	-0.80	-0.27	-0.20	-0.96	1.99	-0.78	-0.52	-0.58	-0.49	-0.17	0.68	0.00	0.29	0.97
TCGA-F7-8298	-0.50	-0.13	-0.80	-0.81	-0.25	1.58	0.74	-1.03	-0.85	-0.40	1.05	-0.01	-0.27	0.89	-1.05	0.21	-0.79	-0.77	-0.92	1.51	-0.17	-0.43	0.00	0.64	0.78
TCGA-F7-848	-0.12	-0.13	-0.10	-0.19	-0.25	1.52	1.30	0.31	-0.64	-0.40	0.14	0.75	-0.27	-0.86	0.60	-0.78	0.27	0.41	-0.92	0.12	-0.17	-0.32	0.01	0.28	0.97

9																									
TCGA-F7-A50G	-0.10	-0.13	-0.59	0.86	-0.25	-1.13	-0.17	0.21	-0.37	-0.40	-0.79	1.52	-0.27	-0.43	0.16	-0.21	1.38	0.26	1.83	-0.49	-0.17	-0.16	0.00	0.39	0.92
TCGA-F7-A50I	-0.50	-0.13	-0.22	0.71	-0.25	-0.37	-0.89	0.47	-0.47	-0.40	-0.79	1.17	-0.27	0.21	0.58	-1.34	0.57	1.72	-0.87	-0.49	-0.17	-0.43	0.00	0.31	0.97
TCGA-F7-A50J	-0.22	-0.13	-0.50	0.61	-0.25	-0.40	0.41	-0.56	-0.85	-0.25	-0.79	0.62	-0.27	0.26	1.05	-0.37	-0.09	0.19	-0.92	0.28	-0.17	-0.07	0.00	0.31	0.98
TCGA-F7-A61S	0.29	-0.13	-0.63	0.23	-0.25	0.95	0.17	-0.88	-0.69	-0.40	2.16	0.49	-0.27	-0.54	1.29	0.54	-0.47	-0.43	-0.92	-0.27	-0.17	-0.43	0.00	0.39	0.93
TCGA-F7-A61V	0.61	-0.13	0.76	0.21	-0.25	-1.04	-0.09	1.08	-0.47	-0.40	-0.79	2.18	-0.27	-0.79	0.48	-0.66	1.72	-0.77	1.15	-0.49	-0.17	-0.43	0.02	0.21	1.00
TCGA-F7-A620	0.98	-0.13	0.23	0.92	-0.25	-0.75	0.76	-0.85	-0.83	-0.30	1.83	1.63	-0.27	-0.89	2.13	-0.22	-0.64	-0.24	-0.90	-0.25	-0.17	-0.43	0.00	0.38	0.94
TCGA-F7-A622	-0.50	-0.13	-0.61	-0.43	0.34	-0.31	0.14	-1.03	-0.85	-0.40	0.78	-0.80	-0.27	-0.03	2.68	1.00	-0.27	-0.75	-0.10	-0.49	-0.17	1.48	0.05	0.14	1.09
TCGA-F7-A623	0.41	-0.13	0.92	-0.16	-0.25	-0.02	2.99	-0.26	-0.85	1.25	0.30	-0.80	-0.27	-1.01	0.81	-0.05	-0.38	-0.43	0.21	-0.49	-0.17	1.40	0.00	0.30	0.96
TCGA-F7-A624	-0.12	-0.13	0.52	2.57	-0.25	-1.13	2.73	-0.96	-0.85	-0.40	0.45	0.86	0.99	-1.31	0.20	0.32	-0.40	-0.77	0.25	-0.49	-0.17	-0.43	0.00	0.56	0.83
TCGA-H7-7774	-0.47	-0.13	-0.36	1.42	-0.25	-0.98	0.83	-0.36	0.68	0.29	-0.79	-0.80	-0.27	-1.16	-0.21	0.57	1.72	0.89	1.25	-0.49	-0.17	-0.43	0.01	0.24	0.98
TCGA-H7-8501	-0.07	-0.13	-0.82	1.62	-0.25	-1.13	5.24	-0.69	-0.81	-0.40	3.43	-0.80	-0.27	-0.81	0.02	0.19	-0.79	-0.69	-0.92	0.75	-0.17	-0.43	0.06	0.14	1.05
TCGA-H7-8502	-0.36	-0.13	-0.81	-1.00	-0.25	2.20	-0.16	-1.03	-0.85	-0.40	2.54	-0.80	-0.05	-0.25	1.62	0.26	-0.79	0.24	-0.92	-0.13	-0.17	0.54	0.00	0.34	0.96
TCGA-H7-A6C4	0.25	-0.13	0.87	-0.13	0.97	-1.02	-0.28	-0.82	0.08	-0.40	-0.79	-0.53	-0.27	-0.02	0.04	-0.67	0.84	1.53	-0.43	0.37	-0.17	0.19	0.01	0.26	0.98
TCGA-H7-A76A	0.21	1.49	0.50	0.40	-0.25	-0.06	-0.68	0.07	2.99	-0.40	0.20	-0.80	2.47	-0.35	0.02	0.28	-0.58	-0.77	-0.46	-0.49	-0.17	-0.43	0.00	0.43	0.90
TCGA-HD-7229	0.24	-0.13	0.28	0.51	-0.25	0.53	0.45	-1.03	-0.85	-0.40	2.76	-0.80	-0.27	-0.39	1.04	-0.88	-0.79	0.82	-0.92	-0.07	-0.17	-0.43	0.00	0.30	0.97
TCGA-HD-7753	-0.50	-0.13	-0.18	-0.55	-0.25	1.66	0.13	-1.03	-0.74	-0.40	0.93	-0.80	-0.27	0.60	-1.04	-0.83	-0.72	1.92	-0.92	0.26	1.58	0.14	0.01	0.27	0.99
TCGA-HD-7754	1.52	-0.13	0.11	-1.00	-0.25	-0.10	-0.74	-0.75	0.52	-0.40	0.63	-0.80	-0.27	1.43	-1.43	0.77	-0.42	-0.40	-0.83	-0.31	-0.17	0.18	0.01	0.28	1.00
TCGA-HD-7831	-0.50	-0.13	0.01	-1.00	-0.25	1.60	-0.93	-1.03	-0.85	-0.40	-0.79	0.31	-0.27	0.73	-1.28	0.26	1.45	-0.72	0.26	-0.49	-0.17	-0.42	0.00	0.55	0.84
TCGA-HD-7832	-0.50	-0.13	-0.66	-0.70	-0.25	0.10	1.58	-1.03	-0.71	-0.40	0.40	0.16	-0.27	0.51	0.63	1.19	-0.79	-0.22	0.07	-0.49	-0.17	-0.43	0.00	0.30	0.98
TCGA-HD-8224	-0.50	-0.13	-0.83	0.04	-0.25	-0.78	-0.93	0.76	-0.26	1.45	-0.79	1.92	-0.27	-0.66	-0.39	2.65	0.49	0.28	1.86	-0.49	-0.17	-0.43	0.12	0.10	1.06
TCGA-HD-83	-0.21	-0.13	-0.28	-0.49	-0.25	-0.89	-0.59	0.10	0.63	-0.40	-0.79	-0.39	-0.27	1.00	-0.39	2.10	-0.63	-0.77	0.76	-0.49	-0.17	-0.43	0.00	0.65	0.77

14																									
TCGA-HD-8634	1.64	-0.13	-0.51	0.00	2.42	-1.10	-0.35	0.03	-0.85	-0.40	-0.45	-0.53	-0.27	0.29	1.09	-0.06	-0.45	0.89	-0.92	0.48	-0.17	0.48	0.01	0.25	1.01
TCGA-HD-8635	-0.50	-0.13	-0.02	-0.70	-0.25	1.59	-0.82	-0.58	-0.30	-0.40	-0.57	0.81	1.06	0.30	0.76	0.22	-0.71	-0.73	-0.28	-0.49	-0.17	0.03	0.00	0.41	0.92
TCGA-HD-A633	-0.50	-0.13	-0.79	-1.00	-0.25	0.94	-0.93	-1.03	-0.28	-0.40	1.63	-0.80	1.38	0.82	-1.19	-0.64	-0.48	-0.43	-0.92	3.99	-0.17	1.11	0.00	0.51	0.86
TCGA-HD-A634	3.85	-0.13	0.06	-0.37	-0.25	-1.13	-0.74	0.14	1.49	-0.40	0.19	-0.80	-0.27	-0.08	-1.43	0.10	-0.79	2.31	-0.92	2.21	3.26	-0.43	0.84	-0.01	1.10
TCGA-HD-A6HZ	-0.33	-0.13	0.18	-1.00	-0.25	2.67	1.12	-1.03	-0.85	-0.40	0.46	-0.80	-0.27	-0.09	-0.30	-0.34	-0.54	0.93	-0.92	-0.25	-0.17	-0.13	0.00	0.38	0.93
TCGA-HD-A6I0	-0.50	1.92	-0.38	1.00	-0.07	-1.13	0.95	0.00	0.93	-0.40	2.86	-0.80	0.47	-0.48	-0.03	0.76	-0.79	-0.67	-0.92	1.38	-0.17	-0.40	0.00	0.31	0.96
TCGA-HL-7533	-0.09	-0.13	-0.83	2.46	-0.25	-1.13	1.84	0.83	0.33	0.67	-0.64	1.05	-0.27	-0.46	1.15	-0.97	-0.56	-0.77	-0.52	-0.49	-0.17	-0.43	0.00	0.39	0.93
TCGA-IQ-7630	-0.50	-0.13	-0.84	-0.88	-0.25	1.61	-0.39	-0.57	-0.57	-0.40	-0.79	1.94	-0.27	-0.30	-0.57	0.96	0.49	1.35	-0.92	0.55	-0.17	-0.24	0.00	0.33	0.95
TCGA-IQ-7631	2.31	-0.13	-0.46	-0.96	-0.25	-0.78	-0.93	-1.03	-0.08	-0.40	-0.79	-0.47	-0.27	3.06	-1.31	-0.76	-0.79	-0.77	-0.13	-0.49	-0.17	-0.43	0.00	0.76	0.66
TCGA-IQ-7632	-0.50	-0.13	3.20	-0.91	-0.25	0.04	0.20	-1.03	-0.63	-0.40	1.14	-0.80	-0.27	-0.12	-0.76	-1.51	-0.79	0.03	-0.92	2.79	-0.17	0.80	0.08	0.12	1.04
TCGA-IQ-A61E	-0.37	-0.13	-0.58	0.72	-0.25	-0.73	1.55	-0.22	-0.85	-0.40	0.67	0.37	-0.27	0.11	-0.36	0.32	-0.37	-0.37	-0.92	1.62	-0.17	-0.43	0.00	0.33	0.95
TCGA-IQ-A61G	-0.50	-0.13	-0.86	-0.85	-0.25	-0.35	-0.93	0.21	0.22	-0.40	-0.79	1.08	-0.27	0.31	-0.89	-0.51	1.01	0.61	-0.92	4.15	3.26	-0.43	0.04	0.16	1.06
TCGA-IQ-A61H	0.79	-0.13	-0.19	-0.38	-0.25	-1.13	-0.74	-0.02	-0.84	0.54	-0.79	-0.49	-0.27	1.15	1.40	-0.52	0.29	-0.77	0.43	-0.49	-0.17	-0.43	0.00	0.44	0.91
TCGA-IQ-A61J	-0.50	0.01	-0.86	-0.65	-0.25	-0.08	-0.91	1.23	-0.03	-0.16	-0.79	1.99	-0.27	0.09	-1.12	-0.64	2.27	0.68	1.91	-0.49	-0.17	-0.43	0.01	0.28	0.97
TCGA-IQ-A61O	-0.50	0.24	-0.86	-0.97	-0.25	-1.03	-0.91	-0.69	0.86	1.86	-0.79	-0.80	-0.27	2.29	-0.49	1.48	-0.77	-0.77	-0.92	-0.05	-0.17	-0.43	0.00	0.76	0.65
TCGA-IQ-A6SG	-0.50	-0.13	-0.86	-1.00	0.46	-1.08	-0.36	0.04	-0.35	0.93	0.77	-0.80	-0.27	2.16	-1.43	0.03	-0.79	-0.09	-0.92	1.98	-0.17	0.28	0.02	0.19	1.10
TCGA-IQ-A6SH	-0.36	-0.13	-0.15	0.36	-0.25	-0.40	-0.30	-1.03	0.76	-0.40	-0.79	3.79	-0.27	-0.57	0.34	1.50	-0.79	0.79	-0.20	-0.49	-0.17	-0.43	0.00	0.46	0.89
TCGA-KU-A66S	0.95	-0.13	0.25	0.01	-0.25	-1.13	-0.64	1.24	0.74	-0.40	-0.77	-0.24	-0.27	0.29	-0.35	0.54	-0.79	0.25	-0.92	1.62	-0.17	-0.43	0.00	0.36	0.94
TCGA-KU-A66T	-0.50	-0.13	0.30	-1.00	-0.25	2.28	-0.93	-1.03	-0.85	-0.40	2.05	-0.80	-0.27	-0.15	-1.43	-0.91	0.36	1.91	-0.92	1.59	0.96	-0.34	0.06	0.13	1.04
TCGA-KU-A6H7	-0.50	-0.13	-0.18	2.89	-0.25	-1.13	1.48	0.42	0.44	0.50	0.32	-0.69	-0.27	-0.84	0.39	-0.42	-0.08	-0.36	-0.53	-0.49	-0.17	-0.43	0.00	0.35	0.95
TCGA-KU-A6	-0.23	-0.13	-0.45	3.28	-0.25	-1.13	-0.12	2.62	-0.75	2.21	-0.79	2.97	-0.27	-1.38	-0.35	0.20	0.07	-0.77	0.05	-0.49	-0.17	-0.43	0.05	0.15	1.06

H8																									
TCGA-MT-A 51W	-0.50	-0.13	-0.86	0.95	-0.25	0.37	0.72	-0.89	-0.85	-0.40	-0.79	2.07	-0.27	-0.65	2.85	-0.84	-0.34	0.10	-0.48	-0.49	-0.17	0.06	0.00	0.38	0.96
TCGA-MT-A 51X	-0.07	-0.13	-0.72	-0.23	-0.25	-0.09	-0.73	-0.08	-0.49	-0.40	-0.79	1.21	-0.27	1.02	-0.19	-1.05	0.90	-0.77	1.32	-0.49	-0.17	-0.07	0.04	0.17	1.05
TCGA-MT-A 67A	-0.50	-0.13	0.72	1.75	-0.25	-1.13	-0.12	1.61	-0.83	-0.40	-0.79	2.56	-0.12	-0.48	-0.11	-0.77	-0.62	-0.77	2.20	-0.49	-0.17	-0.43	0.01	0.26	0.98
TCGA-MT-A 67D	-0.50	-0.13	2.02	-0.61	1.38	0.15	0.03	-1.03	-0.82	-0.40	-0.79	-0.13	-0.27	0.30	-0.26	-0.32	-0.79	0.02	-0.92	0.67	-0.17	0.70	0.00	0.30	0.96
TCGA-MT-A 67F	0.56	-0.13	-0.74	1.68	-0.25	-0.94	3.17	0.77	-0.71	3.95	0.53	-0.80	-0.27	-0.88	-0.52	-0.29	-0.65	-0.12	-0.92	1.67	-0.17	0.13	0.00	0.31	0.96
TCGA-MT-A 7BN	-0.36	-0.13	0.76	1.00	-0.25	-1.13	-0.39	1.43	0.03	-0.40	-0.79	0.77	-0.27	0.10	-0.08	0.83	-0.67	-0.75	-0.43	-0.49	-0.17	-0.43	0.00	0.47	0.88
TCGA-MZ-A 5BI	-0.42	-0.13	0.98	0.12	-0.25	-0.53	0.00	0.77	0.75	-0.40	-0.79	-0.80	0.31	-0.26	-0.25	-1.09	0.26	-0.47	-0.92	2.80	-0.17	0.38	0.00	0.46	0.89
TCGA-MZ-A 6I9	1.03	-0.13	4.83	-0.05	-0.25	-1.00	-0.93	1.68	0.44	0.15	-0.79	0.53	-0.27	-1.09	-0.93	-0.88	-0.40	-0.04	0.53	-0.49	-0.17	-0.20	0.00	0.33	0.94
TCGA-MZ-A 7D7	0.66	-0.13	0.46	-0.50	5.26	0.81	0.11	-1.03	-0.85	-0.40	0.24	-0.70	0.70	-0.75	0.14	-1.71	2.11	-0.77	-0.92	1.13	-0.17	0.39	0.06	0.13	1.03
TCGA-P3-A5 Q5	-0.47	-0.13	-0.37	2.16	-0.25	-1.13	0.83	0.56	1.39	-0.40	-0.79	0.87	-0.27	-0.87	-0.47	1.22	0.16	-0.77	0.08	-0.49	-0.17	-0.25	0.00	0.49	0.87
TCGA-P3-A5 Q6	-0.50	-0.13	-0.42	-0.68	-0.25	0.78	-0.93	-1.03	-0.43	-0.40	-0.79	-0.64	-0.27	1.06	-0.58	1.34	0.26	-0.42	-0.24	-0.49	-0.17	-0.43	0.00	0.40	0.93
TCGA-P3-A5 QA	-0.50	-0.13	-0.47	-1.00	-0.25	1.08	-0.93	-1.03	-0.85	-0.40	-0.79	1.16	-0.27	1.56	-0.66	-1.71	-0.77	-0.77	-0.92	3.58	2.90	-0.43	0.00	0.43	0.92
TCGA-P3-A5 QE	0.71	-0.13	-0.86	0.94	-0.25	2.36	-0.27	0.03	0.98	-0.40	-0.79	0.24	-0.27	-0.98	2.39	-1.71	-0.56	-0.28	-0.81	-0.49	-0.17	-0.43	0.00	0.41	0.93
TCGA-P3-A5 QF	-0.50	-0.13	-0.48	0.35	-0.25	-1.13	0.48	1.97	-0.85	-0.40	-0.79	2.14	0.21	-1.05	0.60	0.72	1.22	0.74	0.88	-0.49	-0.17	-0.43	0.01	0.27	0.98
TCGA-P3-A6 SW	-0.50	-0.13	1.63	1.77	-0.25	-1.13	0.24	-0.19	-0.62	3.75	-0.79	-0.74	-0.27	-0.62	0.18	0.21	-0.67	-0.77	0.73	-0.49	-0.17	-0.43	0.00	0.45	0.89
TCGA-P3-A6 SX	0.36	-0.13	-0.70	-1.00	-0.25	-0.72	-0.93	-0.41	-0.40	0.41	0.35	-0.80	-0.27	3.29	-1.02	-1.71	-0.79	-0.77	-0.92	0.77	-0.17	-0.28	0.00	0.32	1.06
TCGA-P3-A6 T0	-0.50	-0.13	0.03	-1.00	6.25	0.34	-0.36	-1.03	-0.85	-0.40	3.11	-0.80	-0.27	1.17	-1.43	-1.40	-0.67	0.19	-0.92	1.38	1.14	-0.43	0.00	0.35	0.95
TCGA-P3-A6 T2	-0.50	-0.13	-0.59	-0.24	-0.25	0.55	-0.09	-1.03	-0.67	-0.40	0.48	-0.80	-0.27	0.13	-0.29	0.46	-0.79	-0.26	-0.92	4.07	-0.17	-0.41	0.01	0.25	1.01
TCGA-P3-A6 T3	-0.50	-0.13	0.29	-1.00	3.16	0.30	-0.93	-1.03	-0.62	-0.40	-0.61	-0.55	-0.27	1.26	-0.75	-0.35	-0.40	-0.68	-0.92	2.19	-0.17	-0.38	0.02	0.19	1.05
TCGA-P3-A6 T4	-0.50	-0.13	0.08	-1.00	-0.14	-0.08	0.24	-0.32	-0.08	0.98	1.03	-0.80	-0.27	0.60	-0.51	-1.29	0.94	1.21	-0.92	0.14	-0.17	1.18	0.09	0.12	1.06
TCGA-P3-A6	-0.50	-0.13	-0.15	-0.91	-0.25	-1.13	-0.93	1.71	0.26	0.21	-0.79	-0.54	-0.27	1.53	-1.03	0.06	0.25	0.80	-0.92	0.36	-0.17	-0.21	0.00	0.59	0.81

T5																									
TCGA-P3-A6<br>T6	-0.50	-0.13	1.46	-0.73	-0.25	-0.58	-0.82	1.52	1.32	-0.40	-0.79	0.24	-0.27	-0.07	1.31	-1.36	-0.79	1.92	-0.36	-0.49	-0.17	0.12	0.24	0.06	1.09
TCGA-P3-A6<br>T7	-0.50	-0.13	-0.86	0.70	-0.25	-1.13	0.60	0.76	-0.60	-0.40	0.06	-0.05	-0.27	1.08	1.38	-1.21	-0.66	-0.02	-0.92	-0.13	-0.17	-0.43	0.00	0.32	0.99
TCGA-P3-A6<br>T8	-0.50	-0.13	-0.83	-0.98	0.17	1.42	-0.93	-1.03	-0.85	-0.40	0.95	-0.24	-0.27	1.95	-1.43	-1.38	-0.79	2.55	-0.87	-0.49	-0.17	-0.43	0.00	0.51	0.87
TCGA-QK-A6<br>4Z	-0.50	-0.13	-0.78	-0.42	-0.25	1.90	-0.93	-1.03	-0.85	-0.40	-0.79	2.34	-0.27	0.05	1.20	-0.04	-0.06	-0.77	0.19	-0.49	-0.17	-0.40	0.00	0.31	0.98
TCGA-QK-A6<br>52	-0.50	-0.13	-0.86	-0.42	-0.25	-0.48	-0.91	1.94	0.45	0.93	-0.79	-0.28	-0.27	-0.26	1.37	0.33	1.28	-0.50	1.05	-0.49	-0.17	-0.06	0.00	0.37	0.94
TCGA-QK-A6<br>IF	3.36	6.56	0.33	0.43	-0.25	-1.13	0.14	4.23	2.82	3.48	-0.79	-0.80	-0.27	-0.65	-0.31	-1.21	-0.79	-0.77	-0.92	-0.37	-0.17	-0.43	0.00	0.34	0.95
TCGA-QK-A6<br>IG	-0.36	-0.13	-0.12	0.72	-0.25	0.89	-0.18	-0.54	-0.85	-0.40	1.79	0.36	-0.27	-0.48	0.73	0.00	-0.60	-0.77	-0.92	0.33	-0.17	0.62	0.00	0.39	0.93
TCGA-QK-A6<br>IH	-0.30	-0.13	-0.68	-0.15	-0.25	-0.63	-0.93	0.80	-0.85	-0.40	0.01	-0.05	-0.27	1.17	-1.21	1.42	-0.41	-0.07	-0.92	1.09	-0.17	-0.43	0.00	0.45	0.90
TCGA-QK-A6<br>II	-0.50	-0.13	-0.86	0.34	-0.25	-1.13	3.06	0.46	-0.85	-0.40	0.80	0.10	-0.27	0.09	-0.22	-0.07	0.52	0.03	-0.92	0.58	-0.17	-0.31	0.01	0.22	1.01
TCGA-QK-A6<br>IJ	0.28	-0.13	-0.38	-0.75	-0.25	-0.36	0.79	1.58	2.33	-0.40	-0.79	0.44	-0.27	-1.06	0.31	0.98	0.19	0.13	-0.92	1.35	-0.17	1.25	0.00	0.40	0.92
TCGA-QK-A6<br>V9	0.31	-0.13	-0.57	1.49	-0.25	-1.01	1.01	1.00	1.88	0.50	-0.52	-0.80	-0.27	-0.55	2.48	-1.03	-0.74	-0.77	0.06	-0.49	-0.17	-0.43	0.00	0.49	0.88
TCGA-QK-A6<br>VB	0.25	-0.13	-0.20	-1.00	-0.25	-1.13	-0.93	0.23	-0.51	-0.40	-0.68	-0.41	-0.27	3.35	-1.39	-0.79	-0.79	-0.14	-0.92	-0.49	-0.17	-0.43	0.02	0.20	1.15
TCGA-QK-A6<br>VC	-0.24	-0.13	-0.15	0.33	-0.25	-0.08	0.20	2.31	1.43	-0.40	-0.79	-0.29	-0.27	-1.05	2.11	-0.86	-0.24	1.04	-0.12	-0.49	-0.17	-0.09	0.00	0.47	0.89
TCGA-QK-A8<br>Z7	1.70	-0.13	1.01	-0.42	0.07	-1.07	-0.93	-0.06	0.15	-0.40	-0.01	-0.80	-0.27	1.51	-0.20	-0.66	-0.63	-0.77	-0.45	-0.49	-0.17	-0.43	0.00	0.44	0.90
TCGA-QK-A8<br>Z8	-0.42	-0.13	1.59	-0.15	-0.25	-1.13	0.38	1.92	-0.79	-0.16	-0.79	2.73	-0.27	-0.25	-1.03	-1.02	-0.79	1.44	2.42	-0.49	-0.17	-0.19	0.11	0.10	1.04
TCGA-QK-A8<br>Z9	-0.38	-0.13	-0.72	-0.29	-0.25	0.06	-0.93	1.55	0.69	-0.40	-0.79	1.14	-0.27	0.05	-0.41	0.50	1.39	-0.77	0.47	-0.49	-0.17	-0.43	0.00	0.36	0.94
TCGA-QK-A8<br>ZA	-0.50	-0.13	-0.57	-0.96	4.17	0.29	3.33	-1.03	-0.85	-0.40	-0.79	1.77	-0.27	-0.03	-1.05	-0.18	0.05	1.60	-0.92	0.11	-0.17	-0.19	0.07	0.13	1.04
TCGA-QK-A8<br>ZB	-0.50	-0.13	4.36	-0.91	-0.25	-1.09	-0.40	-0.31	-0.69	-0.40	-0.63	-0.80	-0.27	0.71	-0.13	-0.78	-0.79	-0.47	-0.18	-0.49	-0.17	-0.14	0.00	0.43	0.91
TCGA-QK-A<br>A3J	-0.50	-0.13	2.97	0.11	-0.25	-1.13	3.58	-0.27	0.41	-0.40	2.57	-0.80	-0.13	-1.22	-0.27	-0.98	-0.55	-0.08	0.95	-0.49	-0.17	-0.43	0.04	0.16	1.01
TCGA-QK-A<br>A3K	-0.19	-0.13	-0.52	1.45	-0.25	0.46	0.59	-1.03	-0.75	-0.40	0.01	-0.80	-0.27	0.45	-0.70	0.76	-0.79	-0.77	-0.92	-0.04	-0.17	-0.43	0.00	0.49	0.87
TCGA-RS-A6	-0.38	-0.13	-0.83	0.67	-0.25	-1.13	2.39	-0.48	-0.85	-0.40	1.86	-0.80	-0.27	0.30	0.92	1.13	-0.79	-0.57	-0.92	-0.49	-0.17	-0.43	0.00	0.60	0.80

TO													
TCGA-RS-A6TP	-0.31	-0.13	-0.85	0.87	-0.25	-1.13	-0.18	1.92	-0.67	-0.40	-0.79	6.33	-0.27
TCGA-T2-A6WX	-0.45	-0.13	0.24	3.25	-0.25	-1.13	1.15	-0.16	0.73	-0.40	-0.17	0.00	-0.27
TCGA-T2-A6WZ	-0.50	-0.13	3.15	-0.05	-0.25	-0.71	-0.90	0.97	0.86	0.32	-0.79	1.36	-0.27
TCGA-T2-A6X0	0.75	-0.13	0.90	1.72	-0.25	-1.13	2.25	-0.82	-0.45	-0.11	0.53	-0.80	-0.27
TCGA-T2-A6X2	-0.28	-0.13	-0.61	0.95	-0.25	-0.80	2.43	-0.63	-0.63	-0.40	3.36	0.06	0.29
TCGA-T3-A92M	-0.50	-0.13	0.52	0.32	-0.25	-0.85	0.49	1.23	1.22	-0.28	-0.79	0.48	-0.27
TCGA-T3-A92N	-0.44	-0.13	-0.77	0.59	-0.25	0.05	1.35	1.04	-0.47	-0.40	-0.47	0.32	-0.01
TCGA-TN-A7HI	-0.50	0.77	2.13	0.99	-0.25	-1.13	0.70	0.36	1.32	-0.40	-0.79	0.25	-0.27
TCGA-TN-A7HJ	0.59	-0.13	2.42	-0.80	1.13	-0.33	-0.93	0.14	0.33	-0.40	-0.05	-0.80	-0.27
TCGA-TN-A7HL	-0.02	-0.13	-0.38	1.97	-0.25	-1.13	1.31	0.95	1.04	-0.40	3.50	-0.80	-0.05
TCGA-UF-A718	-0.50	-0.13	0.78	-0.24	-0.25	0.15	1.29	1.13	0.76	0.64	-0.79	-0.24	-0.27
TCGA-UF-A719	-0.50	-0.13	0.01	0.07	-0.25	-1.13	-0.50	1.62	0.15	-0.40	-0.77	1.47	-0.27
TCGA-UF-A71A	-0.33	-0.13	-0.56	-0.85	3.01	1.10	-0.93	-1.03	-0.85	-0.40	0.81	-0.80	-0.27
TCGA-UF-A71B	-0.50	-0.13	1.44	-0.78	-0.25	0.16	-0.93	-1.03	-0.39	-0.40	-0.62	-0.37	-0.27
TCGA-UF-A71D	-0.10	-0.13	2.42	0.35	-0.25	-1.13	1.48	0.64	-0.20	-0.40	-0.79	-0.14	-0.27
TCGA-UF-A71E	-0.38	-0.13	3.38	-1.00	-0.25	-1.13	-0.93	0.39	-0.22	0.29	-0.17	-0.80	-0.27
TCGA-UF-A7J9	-0.47	-0.13	-0.60	-1.00	-0.25	0.95	-0.40	-1.03	-0.54	-0.40	-0.53	0.15	-0.27
TCGA-UF-A7JA	-0.50	-0.13	-0.31	-0.75	-0.25	-0.43	-0.93	0.63	-0.18	-0.40	0.91	-0.80	-0.27
TCGA-UF-A7JC	-0.45	-0.13	-0.63	-0.92	2.28	-0.64	-0.93	-0.86	-0.85	-0.40	1.05	-0.80	-0.27
TCGA-UF-A7JD	-0.33	-0.13	0.04	-0.80	0.34	-0.84	-0.93	-0.87	-0.40	-0.40	0.39	-0.80	-0.27
TCGA-UF-A7	-0.50	0.17	0.05	-0.69	-0.25	-0.47	-0.93	-0.23	-0.17	-0.40	-0.79	1.68	-0.27

TO												
TCGA-RS-A6TP	-0.55	2.64	-0.95	-0.79	-0.15	-0.48	-0.49	-0.17	-0.43	0.00	0.29	1.03
TCGA-T2-A6WX	-1.15	1.44	-0.28	-0.71	-0.77	-0.65	-0.49	-0.17	-0.38	0.00	0.35	0.96
TCGA-T2-A6WZ	-0.32	-0.74	-0.07	-0.50	-0.61	-0.08	-0.49	-0.17	-0.43	0.00	0.34	0.94
TCGA-T2-A6X0	-0.94	1.13	0.08	-0.79	-0.77	0.25	-0.49	-0.17	-0.43	0.00	0.45	0.89
TCGA-T2-A6X2	-1.06	0.82	0.72	-0.02	-0.77	0.93	-0.49	-0.17	-0.43	0.00	0.35	0.94
TCGA-T3-A92M	-0.51	1.21	0.39	-0.04	-0.76	-0.42	-0.49	-0.17	-0.43	0.00	0.49	0.87
TCGA-T3-A92N	-1.15	3.40	0.37	-0.36	-0.65	-0.49	-0.49	-0.17	-0.30	0.00	0.43	0.92
TCGA-TN-A7HI	-0.29	-0.22	-0.19	-0.77	-0.65	-0.62	-0.49	-0.17	-0.43	0.00	0.57	0.84
TCGA-TN-A7HJ	-0.40	-0.88	0.64	-0.18	1.23	-0.92	-0.18	-0.17	0.76	0.22	0.07	1.05
TCGA-TN-A7HL	-0.91	0.72	-0.53	-0.35	-0.77	-0.13	-0.49	-0.17	-0.43	0.00	0.63	0.79
TCGA-UF-A718	-0.38	-0.05	-0.23	-0.39	0.21	-0.92	0.34	-0.17	-0.43	0.00	0.30	0.96
TCGA-UF-A719	1.17	-1.14	0.82	-0.79	0.12	-0.92	-0.49	-0.17	-0.43	0.35	0.04	1.12
TCGA-UF-A71A	0.99	-1.07	1.34	-0.05	-0.43	-0.88	-0.35	-0.17	-0.43	0.00	0.47	0.89
TCGA-UF-A71B	0.67	-1.18	0.78	-0.47	-0.57	-0.92	1.56	-0.17	0.82	0.00	0.37	0.94
TCGA-UF-A71D	-0.49	-0.81	-0.42	-0.42	0.64	0.76	-0.49	-0.17	-0.09	0.50	0.02	1.05
TCGA-UF-A71E	1.06	-1.27	-0.33	-0.14	-0.56	-0.92	0.06	-0.17	-0.43	0.01	0.24	1.01
TCGA-UF-A7J9	1.99	-0.81	0.58	-0.79	-0.77	-0.92	-0.49	-0.17	-0.27	0.00	0.54	0.85
TCGA-UF-A7JA	2.23	-0.27	-0.57	-0.76	-0.77	-0.92	-0.26	-0.17	-0.20	0.00	0.52	0.87
TCGA-UF-A7JC	1.89	-0.10	-0.53	0.18	0.12	-0.92	0.16	1.93	-0.43	0.01	0.23	1.06
TCGA-UF-A7JD	1.39	-1.20	-0.37	-0.79	-0.77	-0.92	5.16	-0.17	-0.18	0.01	0.27	1.04
TCGA-UF-A7	0.75	-0.94	0.31	2.15	-0.77	-0.86	-0.06	-0.17	-0.43	0.00	0.38	0.94

JF																									
TCGA-UF-A7 JH	-0.50	0.62	-0.73	0.04	-0.25	-0.87	-0.69	2.53	-0.39	-0.02	-0.79	0.66	-0.27	0.72	0.44	0.64	0.24	-0.77	-0.92	-0.49	-0.17	-0.43	0.00	0.29	0.99
TCGA-UF-A7 JJ	-0.47	-0.13	1.13	0.40	-0.25	-0.22	0.66	-0.68	-0.50	2.50	-0.79	-0.80	-0.27	0.01	-0.85	0.62	-0.43	0.21	-0.14	-0.49	-0.17	0.02	0.00	0.60	0.82
TCGA-UF-A7 JK	-0.50	-0.13	-0.19	-0.51	8.20	0.65	-0.93	-1.03	-0.85	-0.40	-0.79	-0.12	-0.27	1.65	-1.12	-1.68	0.36	-0.77	-0.92	-0.25	-0.17	-0.42	0.00	0.29	1.00
TCGA-UF-A7 JO	0.00	-0.13	-0.86	0.31	-0.25	-0.59	0.43	0.39	-0.04	-0.40	0.83	-0.57	-0.27	0.06	1.02	0.58	-0.23	0.01	-0.08	-0.49	-0.17	-0.28	0.00	0.53	0.85
TCGA-UF-A7 JS	-0.50	3.66	-0.67	-0.35	-0.25	0.26	0.03	-1.03	0.88	-0.40	-0.79	2.11	-0.27	0.53	-0.86	1.34	-0.79	-0.12	-0.92	-0.24	-0.17	0.22	0.01	0.27	0.99
TCGA-UF-A7 JT	-0.19	-0.13	-0.17	1.86	-0.25	-1.13	0.73	0.04	0.54	-0.40	1.88	-0.18	-0.27	-0.71	1.64	0.39	-0.78	-0.77	-0.92	-0.49	-0.17	-0.43	0.00	0.36	0.95
TCGA-UF-A7 JV	-0.50	-0.13	-0.86	-0.84	-0.25	0.08	-0.93	-1.03	0.01	-0.40	0.02	-0.80	-0.27	1.75	-0.44	-0.36	-0.79	-0.45	-0.92	2.92	-0.17	-0.43	0.00	0.33	0.99
TCGA-UP-A6 WW	-0.17	-0.13	0.18	-0.44	-0.25	-0.81	-0.67	1.07	1.88	0.48	-0.79	-0.50	-0.27	0.80	-0.49	0.02	0.43	-0.64	-0.01	-0.49	-0.17	-0.43	0.00	0.47	0.88
TCGA-WA-A 7GZ	-0.50	-0.13	-0.86	-0.82	-0.25	2.25	-0.93	-1.03	-0.85	-0.40	-0.79	0.48	-0.27	0.91	-1.29	-0.99	-0.48	3.01	0.40	-0.49	-0.17	-0.43	0.00	0.59	0.81
TCGA-WA-A 7H4	-0.50	-0.13	0.28	-1.00	1.73	0.55	-0.93	-1.03	-0.28	-0.40	-0.79	0.32	-0.27	0.65	-0.77	-0.34	0.96	-0.01	1.27	-0.49	-0.17	-0.43	0.01	0.23	1.00

Table S2: 22 immune cell scores and prognosis in TME

	p.value	HR	Low.95.CI	High.95.CI
B cells naive	0.086548	0.863165	0.729478	1.021352
B cells memory	0.660853	0.955225	0.778452	1.17214
Plasma cells	0.069047	0.8604	0.731654	1.0118
T cells CD8	0.037925	0.853726	0.735302	0.991224
T cells CD4 naive	0.133356	1.093217	0.973116	1.22814
T cells CD4 memory resting	0.108134	1.12081	0.97523	1.288122
T cells CD4 memory activated	0.010586	0.819213	0.703058	0.954559
T cells follicular helper	0.004051	0.802667	0.690934	0.93247
T cells regulatory (Tregs)	0.003828	0.79889	0.68612	0.930195
T cells gamma delta	0.012931	0.774562	0.633251	0.947405
NK cells resting	0.452178	0.950007	0.831111	1.085912
NK cells activated	0.531778	1.045268	0.909828	1.200869
Monocytes	0.37787	0.864368	0.625178	1.19507
Macrophages M0	0.006389	1.198808	1.052333	1.36567
Macrophages M1	0.628357	1.035183	0.899933	1.190759
Macrophages M2	0.189327	1.115496	0.947518	1.313254
Dendritic cells resting	0.487793	1.062136	0.895827	1.259321

Dendritic cells activated	0.844445	0.984634	0.84352	1.149354
Mast cells resting	0.006784	0.793448	0.671079	0.938131
Mast cells activated	0.053651	1.123925	0.998166	1.265528
Eosinophils	0.00512	1.150857	1.043033	1.269828
Neutrophils	0.361275	1.05902	0.93635	1.197761

Table S3: Details of TMEC1 and TMEC2

cluster.Sample	cluster.TMECluster	cluster.Sample	cluster.TMECluster
TCGA-4P-AA8J	TMEC1	TCGA-CN-5374	TMEC2
TCGA-BA-4074	TMEC1	TCGA-CN-6010	TMEC2
TCGA-BA-4075	TMEC1	TCGA-CN-6011	TMEC2
TCGA-BA-4076	TMEC1	TCGA-CN-6012	TMEC2
TCGA-BA-5151	TMEC1	TCGA-CN-6013	TMEC2
TCGA-BA-5557	TMEC1	TCGA-CN-6017	TMEC2
TCGA-BA-6868	TMEC1	TCGA-CN-6021	TMEC2
TCGA-BA-6870	TMEC1	TCGA-CN-6023	TMEC2
TCGA-BA-6872	TMEC1	TCGA-CN-6995	TMEC2
TCGA-BA-A4IF	TMEC1	TCGA-CN-6996	TMEC2
TCGA-BA-A6D8	TMEC1	TCGA-CN-6997	TMEC2
TCGA-BA-A6DE	TMEC1	TCGA-CN-A497	TMEC2
TCGA-BA-A6DG	TMEC1	TCGA-CN-A498	TMEC2
TCGA-BA-A6DI	TMEC1	TCGA-CN-A499	TMEC2
TCGA-BA-A8YP	TMEC1	TCGA-CN-A49B	TMEC2
TCGA-BB-4224	TMEC1	TCGA-CN-A49C	TMEC2
TCGA-BB-4227	TMEC1	TCGA-CN-A63T	TMEC2
TCGA-BB-8596	TMEC1	TCGA-CN-A63U	TMEC2
TCGA-BB-A5HU	TMEC1	TCGA-CN-A641	TMEC2
TCGA-BB-A5HY	TMEC1	TCGA-CN-A642	TMEC2
TCGA-BB-A6UO	TMEC1	TCGA-CN-A6UY	TMEC2
TCGA-C9-A47Z	TMEC1	TCGA-CN-A6V3	TMEC2
TCGA-CN-4725	TMEC1	TCGA-CN-A6V6	TMEC2
TCGA-CN-4726	TMEC1	TCGA-CN-A6V7	TMEC2
TCGA-CN-4727	TMEC1	TCGA-CQ-5323	TMEC2
TCGA-CN-4728	TMEC1	TCGA-CQ-5324	TMEC2
TCGA-CN-4730	TMEC1	TCGA-CQ-5325	TMEC2
TCGA-CN-4731	TMEC1	TCGA-CQ-5327	TMEC2
TCGA-CN-4735	TMEC1	TCGA-CQ-5329	TMEC2
TCGA-CN-4737	TMEC1	TCGA-CQ-5330	TMEC2
TCGA-CN-4740	TMEC1	TCGA-CQ-5331	TMEC2
TCGA-CN-4742	TMEC1	TCGA-CQ-5333	TMEC2
TCGA-CN-5355	TMEC1	TCGA-CQ-5334	TMEC2

TCGA-CN-5359	TMEC1	TCGA-CQ-6219	TMEC2
TCGA-CN-5364	TMEC1	TCGA-CQ-6220	TMEC2
TCGA-CN-5367	TMEC1	TCGA-CQ-6223	TMEC2
TCGA-CN-5370	TMEC1	TCGA-CQ-6229	TMEC2
TCGA-CN-6016	TMEC1	TCGA-CQ-7063	TMEC2
TCGA-CN-6018	TMEC1	TCGA-CQ-7065	TMEC2
TCGA-CN-6019	TMEC1	TCGA-CQ-7068	TMEC2
TCGA-CN-6020	TMEC1	TCGA-CQ-7071	TMEC2
TCGA-CN-6022	TMEC1	TCGA-CQ-A4C6	TMEC2
TCGA-CN-6024	TMEC1	TCGA-CQ-A4CE	TMEC2
TCGA-CN-6988	TMEC1	TCGA-CR-5243	TMEC2
TCGA-CN-6989	TMEC1	TCGA-CR-5248	TMEC2
TCGA-CN-6992	TMEC1	TCGA-CR-5249	TMEC2
TCGA-CN-6994	TMEC1	TCGA-CR-5250	TMEC2
TCGA-CN-6998	TMEC1	TCGA-CR-6467	TMEC2
TCGA-CN-A49A	TMEC1	TCGA-CR-6470	TMEC2
TCGA-CN-A63V	TMEC1	TCGA-CR-6471	TMEC2
TCGA-CN-A63W	TMEC1	TCGA-CR-6472	TMEC2
TCGA-CQ-5326	TMEC1	TCGA-CR-6473	TMEC2
TCGA-CQ-5332	TMEC1	TCGA-CR-6478	TMEC2
TCGA-CQ-6218	TMEC1	TCGA-CR-6480	TMEC2
TCGA-CQ-6221	TMEC1	TCGA-CR-6481	TMEC2
TCGA-CQ-6222	TMEC1	TCGA-CR-6482	TMEC2
TCGA-CQ-6224	TMEC1	TCGA-CR-6484	TMEC2
TCGA-CQ-6225	TMEC1	TCGA-CR-6487	TMEC2
TCGA-CQ-6227	TMEC1	TCGA-CR-6491	TMEC2
TCGA-CQ-6228	TMEC1	TCGA-CR-6492	TMEC2
TCGA-CQ-7069	TMEC1	TCGA-CR-7364	TMEC2
TCGA-CQ-7072	TMEC1	TCGA-CR-7367	TMEC2
TCGA-CQ-A4C7	TMEC1	TCGA-CR-7368	TMEC2
TCGA-CQ-A4C9	TMEC1	TCGA-CR-7370	TMEC2
TCGA-CQ-A4CB	TMEC1	TCGA-CR-7371	TMEC2
TCGA-CQ-A4CD	TMEC1	TCGA-CR-7373	TMEC2
TCGA-CQ-A4CG	TMEC1	TCGA-CR-7376	TMEC2
TCGA-CQ-A4CH	TMEC1	TCGA-CR-7377	TMEC2
TCGA-CQ-A4CI	TMEC1	TCGA-CR-7380	TMEC2
TCGA-CR-5247	TMEC1	TCGA-CR-7382	TMEC2
TCGA-CR-6474	TMEC1	TCGA-CR-7383	TMEC2
TCGA-CR-6477	TMEC1	TCGA-CR-7385	TMEC2
TCGA-CR-6488	TMEC1	TCGA-CR-7386	TMEC2
TCGA-CR-6493	TMEC1	TCGA-CR-7388	TMEC2
TCGA-CR-7365	TMEC1	TCGA-CR-7389	TMEC2

TCGA-CR-7369	TMEC1	TCGA-CR-7391	TMEC2
TCGA-CR-7372	TMEC1	TCGA-CR-7392	TMEC2
TCGA-CR-7379	TMEC1	TCGA-CR-7393	TMEC2
TCGA-CR-7390	TMEC1	TCGA-CR-7394	TMEC2
TCGA-CV-5434	TMEC1	TCGA-CR-7395	TMEC2
TCGA-CV-5435	TMEC1	TCGA-CR-7397	TMEC2
TCGA-CV-5440	TMEC1	TCGA-CR-7398	TMEC2
TCGA-CV-5444	TMEC1	TCGA-CR-7399	TMEC2
TCGA-CV-5970	TMEC1	TCGA-CR-7401	TMEC2
TCGA-CV-5973	TMEC1	TCGA-CR-7402	TMEC2
TCGA-CV-5976	TMEC1	TCGA-CR-7404	TMEC2
TCGA-CV-5977	TMEC1	TCGA-CV-5430	TMEC2
TCGA-CV-5978	TMEC1	TCGA-CV-5431	TMEC2
TCGA-CV-6003	TMEC1	TCGA-CV-5432	TMEC2
TCGA-CV-6933	TMEC1	TCGA-CV-5436	TMEC2
TCGA-CV-6935	TMEC1	TCGA-CV-5439	TMEC2
TCGA-CV-6937	TMEC1	TCGA-CV-5441	TMEC2
TCGA-CV-6940	TMEC1	TCGA-CV-5442	TMEC2
TCGA-CV-6941	TMEC1	TCGA-CV-5443	TMEC2
TCGA-CV-6945	TMEC1	TCGA-CV-5966	TMEC2
TCGA-CV-6948	TMEC1	TCGA-CV-5971	TMEC2
TCGA-CV-6950	TMEC1	TCGA-CV-5979	TMEC2
TCGA-CV-6956	TMEC1	TCGA-CV-6433	TMEC2
TCGA-CV-6959	TMEC1	TCGA-CV-6436	TMEC2
TCGA-CV-6960	TMEC1	TCGA-CV-6441	TMEC2
TCGA-CV-6961	TMEC1	TCGA-CV-6934	TMEC2
TCGA-CV-6962	TMEC1	TCGA-CV-6936	TMEC2
TCGA-CV-7089	TMEC1	TCGA-CV-6938	TMEC2
TCGA-CV-7095	TMEC1	TCGA-CV-6939	TMEC2
TCGA-CV-7097	TMEC1	TCGA-CV-6942	TMEC2
TCGA-CV-7101	TMEC1	TCGA-CV-6943	TMEC2
TCGA-CV-7102	TMEC1	TCGA-CV-6951	TMEC2
TCGA-CV-7103	TMEC1	TCGA-CV-6952	TMEC2
TCGA-CV-7104	TMEC1	TCGA-CV-6953	TMEC2
TCGA-CV-7177	TMEC1	TCGA-CV-6954	TMEC2
TCGA-CV-7183	TMEC1	TCGA-CV-6955	TMEC2
TCGA-CV-7236	TMEC1	TCGA-CV-7090	TMEC2
TCGA-CV-7245	TMEC1	TCGA-CV-7091	TMEC2
TCGA-CV-7247	TMEC1	TCGA-CV-7099	TMEC2
TCGA-CV-7252	TMEC1	TCGA-CV-7100	TMEC2
TCGA-CV-7253	TMEC1	TCGA-CV-7178	TMEC2
TCGA-CV-7255	TMEC1	TCGA-CV-7180	TMEC2

TCGA-CV-7261	TMEC1	TCGA-CV-7235	TMEC2
TCGA-CV-7263	TMEC1	TCGA-CV-7238	TMEC2
TCGA-CV-7406	TMEC1	TCGA-CV-7242	TMEC2
TCGA-CV-7411	TMEC1	TCGA-CV-7248	TMEC2
TCGA-CV-7416	TMEC1	TCGA-CV-7250	TMEC2
TCGA-CV-7422	TMEC1	TCGA-CV-7254	TMEC2
TCGA-CV-7423	TMEC1	TCGA-CV-7407	TMEC2
TCGA-CV-7429	TMEC1	TCGA-CV-7410	TMEC2
TCGA-CV-7430	TMEC1	TCGA-CV-7413	TMEC2
TCGA-CV-7435	TMEC1	TCGA-CV-7415	TMEC2
TCGA-CV-7438	TMEC1	TCGA-CV-7418	TMEC2
TCGA-CV-7440	TMEC1	TCGA-CV-7424	TMEC2
TCGA-CV-A45P	TMEC1	TCGA-CV-7425	TMEC2
TCGA-CV-A45X	TMEC1	TCGA-CV-7427	TMEC2
TCGA-CV-A465	TMEC1	TCGA-CV-7428	TMEC2
TCGA-CV-A6JM	TMEC1	TCGA-CV-7432	TMEC2
TCGA-CV-A6JY	TMEC1	TCGA-CV-7433	TMEC2
TCGA-CV-A6K2	TMEC1	TCGA-CV-7434	TMEC2
TCGA-CX-7219	TMEC1	TCGA-CV-7437	TMEC2
TCGA-D6-6517	TMEC1	TCGA-CV-7446	TMEC2
TCGA-D6-6823	TMEC1	TCGA-CV-7568	TMEC2
TCGA-D6-6826	TMEC1	TCGA-CV-A45O	TMEC2
TCGA-D6-6827	TMEC1	TCGA-CV-A45Q	TMEC2
TCGA-D6-8568	TMEC1	TCGA-CV-A45R	TMEC2
TCGA-D6-A4Z9	TMEC1	TCGA-CV-A45T	TMEC2
TCGA-D6-A4ZB	TMEC1	TCGA-CV-A45U	TMEC2
TCGA-D6-A6ES	TMEC1	TCGA-CV-A45V	TMEC2
TCGA-DQ-5624	TMEC1	TCGA-CV-A45W	TMEC2
TCGA-DQ-5629	TMEC1	TCGA-CV-A45Y	TMEC2
TCGA-DQ-5631	TMEC1	TCGA-CV-A45Z	TMEC2
TCGA-DQ-7588	TMEC1	TCGA-CV-A460	TMEC2
TCGA-F7-8298	TMEC1	TCGA-CV-A461	TMEC2
TCGA-F7-A50I	TMEC1	TCGA-CV-A464	TMEC2
TCGA-H7-8502	TMEC1	TCGA-CV-A468	TMEC2
TCGA-H7-A6C4	TMEC1	TCGA-CV-A6JD	TMEC2
TCGA-HD-7753	TMEC1	TCGA-CV-A6JE	TMEC2
TCGA-HD-7754	TMEC1	TCGA-CV-A6JN	TMEC2
TCGA-HD-7831	TMEC1	TCGA-CV-A6JO	TMEC2
TCGA-HD-8314	TMEC1	TCGA-CV-A6JT	TMEC2
TCGA-HD-8634	TMEC1	TCGA-CV-A6JU	TMEC2
TCGA-HD-8635	TMEC1	TCGA-CV-A6JZ	TMEC2
TCGA-HD-A633	TMEC1	TCGA-CV-A6K0	TMEC2

TCGA-HD-A634	TMEC1	TCGA-CV-A6K1	TMEC2
TCGA-IQ-7630	TMEC1	TCGA-CX-7085	TMEC2
TCGA-IQ-7631	TMEC1	TCGA-CX-7086	TMEC2
TCGA-IQ-7632	TMEC1	TCGA-CX-A4AQ	TMEC2
TCGA-IQ-A61G	TMEC1	TCGA-D6-6515	TMEC2
TCGA-IQ-A61H	TMEC1	TCGA-D6-6516	TMEC2
TCGA-IQ-A61O	TMEC1	TCGA-D6-6824	TMEC2
TCGA-IQ-A6SG	TMEC1	TCGA-D6-6825	TMEC2
TCGA-KU-A66T	TMEC1	TCGA-D6-8569	TMEC2
TCGA-MT-A51X	TMEC1	TCGA-D6-A6EK	TMEC2
TCGA-MT-A67D	TMEC1	TCGA-D6-A6EM	TMEC2
TCGA-P3-A5Q6	TMEC1	TCGA-D6-A6EN	TMEC2
TCGA-P3-A5QA	TMEC1	TCGA-D6-A6EO	TMEC2
TCGA-P3-A6SX	TMEC1	TCGA-D6-A6EP	TMEC2
TCGA-P3-A6T0	TMEC1	TCGA-D6-A6EQ	TMEC2
TCGA-P3-A6T2	TMEC1	TCGA-D6-A74Q	TMEC2
TCGA-P3-A6T3	TMEC1	TCGA-DQ-5625	TMEC2
TCGA-P3-A6T4	TMEC1	TCGA-DQ-5630	TMEC2
TCGA-P3-A6T5	TMEC1	TCGA-DQ-7591	TMEC2
TCGA-P3-A6T7	TMEC1	TCGA-DQ-7592	TMEC2
TCGA-P3-A6T8	TMEC1	TCGA-F7-7848	TMEC2
TCGA-QK-A64Z	TMEC1	TCGA-F7-8489	TMEC2
TCGA-QK-A6IH	TMEC1	TCGA-F7-A50G	TMEC2
TCGA-QK-A6VB	TMEC1	TCGA-F7-A50J	TMEC2
TCGA-QK-A8Z7	TMEC1	TCGA-F7-A61S	TMEC2
TCGA-QK-A8ZB	TMEC1	TCGA-F7-A61V	TMEC2
TCGA-UF-A719	TMEC1	TCGA-F7-A620	TMEC2
TCGA-UF-A71A	TMEC1	TCGA-F7-A622	TMEC2
TCGA-UF-A71B	TMEC1	TCGA-F7-A623	TMEC2
TCGA-UF-A71E	TMEC1	TCGA-F7-A624	TMEC2
TCGA-UF-A7J9	TMEC1	TCGA-H7-7774	TMEC2
TCGA-UF-A7JA	TMEC1	TCGA-H7-8501	TMEC2
TCGA-UF-A7JC	TMEC1	TCGA-H7-A76A	TMEC2
TCGA-UF-A7JD	TMEC1	TCGA-HD-7229	TMEC2
TCGA-UF-A7JF	TMEC1	TCGA-HD-7832	TMEC2
TCGA-UF-A7JH	TMEC1	TCGA-HD-8224	TMEC2
TCGA-UF-A7JK	TMEC1	TCGA-HD-A6HZ	TMEC2
TCGA-UF-A7JS	TMEC1	TCGA-HD-A6I0	TMEC2
TCGA-UF-A7JV	TMEC1	TCGA-HL-7533	TMEC2
TCGA-WA-A7GZ	TMEC1	TCGA-IQ-A61E	TMEC2
TCGA-WA-A7H4	TMEC1	TCGA-IQ-A61J	TMEC2
TCGA-BA-4077	TMEC2	TCGA-IQ-A6SH	TMEC2

TCGA-BA-4078	TMEC2	TCGA-KU-A66S	TMEC2
TCGA-BA-5152	TMEC2	TCGA-KU-A6H7	TMEC2
TCGA-BA-5153	TMEC2	TCGA-KU-A6H8	TMEC2
TCGA-BA-5555	TMEC2	TCGA-MT-A51W	TMEC2
TCGA-BA-5556	TMEC2	TCGA-MT-A67A	TMEC2
TCGA-BA-5558	TMEC2	TCGA-MT-A67F	TMEC2
TCGA-BA-5559	TMEC2	TCGA-MT-A7BN	TMEC2
TCGA-BA-6869	TMEC2	TCGA-MZ-A5BI	TMEC2
TCGA-BA-6871	TMEC2	TCGA-MZ-A6I9	TMEC2
TCGA-BA-6873	TMEC2	TCGA-MZ-A7D7	TMEC2
TCGA-BA-7269	TMEC2	TCGA-P3-A5Q5	TMEC2
TCGA-BA-A4IG	TMEC2	TCGA-P3-A5QE	TMEC2
TCGA-BA-A4IH	TMEC2	TCGA-P3-A5QF	TMEC2
TCGA-BA-A4II	TMEC2	TCGA-P3-A6SW	TMEC2
TCGA-BA-A6DA	TMEC2	TCGA-P3-A6T6	TMEC2
TCGA-BA-A6DB	TMEC2	TCGA-QK-A652	TMEC2
TCGA-BA-A6DD	TMEC2	TCGA-QK-A6IF	TMEC2
TCGA-BA-A6DJ	TMEC2	TCGA-QK-A6IG	TMEC2
TCGA-BA-A6DL	TMEC2	TCGA-QK-A6II	TMEC2
TCGA-BB-4217	TMEC2	TCGA-QK-A6IJ	TMEC2
TCGA-BB-4223	TMEC2	TCGA-QK-A6V9	TMEC2
TCGA-BB-4225	TMEC2	TCGA-QK-A6VC	TMEC2
TCGA-BB-4228	TMEC2	TCGA-QK-A8Z8	TMEC2
TCGA-BB-8601	TMEC2	TCGA-QK-A8Z9	TMEC2
TCGA-BB-A5HZ	TMEC2	TCGA-QK-A8ZA	TMEC2
TCGA-BB-A6UM	TMEC2	TCGA-QK-AA3J	TMEC2
TCGA-C9-A480	TMEC2	TCGA-QK-AA3K	TMEC2
TCGA-CN-4722	TMEC2	TCGA-RS-A6TO	TMEC2
TCGA-CN-4723	TMEC2	TCGA-RS-A6TP	TMEC2
TCGA-CN-4729	TMEC2	TCGA-T2-A6WX	TMEC2
TCGA-CN-4733	TMEC2	TCGA-T2-A6WZ	TMEC2
TCGA-CN-4734	TMEC2	TCGA-T2-A6X0	TMEC2
TCGA-CN-4736	TMEC2	TCGA-T2-A6X2	TMEC2
TCGA-CN-4738	TMEC2	TCGA-T3-A92M	TMEC2
TCGA-CN-4739	TMEC2	TCGA-T3-A92N	TMEC2
TCGA-CN-4741	TMEC2	TCGA-TN-A7HI	TMEC2
TCGA-CN-5356	TMEC2	TCGA-TN-A7HJ	TMEC2
TCGA-CN-5358	TMEC2	TCGA-TN-A7HL	TMEC2
TCGA-CN-5360	TMEC2	TCGA-UF-A718	TMEC2
TCGA-CN-5361	TMEC2	TCGA-UF-A71D	TMEC2
TCGA-CN-5363	TMEC2	TCGA-UF-A7JJ	TMEC2
TCGA-CN-5365	TMEC2	TCGA-UF-A7JO	TMEC2

TCGA-CN-5366	TMEC2	TCGA-UF-A7JT	TMEC2
TCGA-CN-5369	TMEC2	TCGA-UP-A6WW	TMEC2
TCGA-CN-5373	TMEC2		

Table S4: DEGs analysis between TMEC1 and TMEC2

Gene symbol	logFC	P-value	Gene symbol	logFC	P-value
ZMYND10	-1.01773	1.19E-11	AMICA1	-1.06769	9.23E-24
ITGAL	-1.63474	1.47E-45	CD3G	-2.04764	2.58E-76
YBX2	-1.25614	4.01E-08	CXCR5	-2.08899	2.15E-26
CEACAM21	-1.13797	1.63E-21	CCR5	-1.64649	7.59E-45
NOS2	-2.25459	3.74E-28	FCRL3	-1.93298	7.25E-37
CD79B	-1.6687	5.53E-37	SCGB3A1	-1.48198	4.71E-06
SCN4A	1.11825	2.28E-05	NPHS1	-1.64608	2.10E-11
TKTL1	-1.74822	4.78E-08	IKZF3	-2.05953	2.22E-64
FMO3	-1.4527	8.60E-17	CCDC155	-2.48062	1.53E-15
ETV7	-1.484	2.15E-43	SYCE2	-1.51082	4.78E-24
CD22	-1.37446	1.06E-19	IP6K3	1.185645	8.71E-07
LTF	-1.31426	2.46E-05	ALOX15	-1.47442	2.14E-10
CD6	-1.52901	3.55E-51	SCIMP	-1.25626	4.69E-20
WAS	-1.09603	1.30E-30	GBP2	-1.11611	6.76E-35
ISL1	-1.27661	1.77E-06	GBP4	-1.86036	5.81E-52
CD74	-1.45635	5.43E-40	BRINP3	1.771178	2.64E-06
SLAMF7	-1.21764	5.25E-26	VCAM1	-1.85972	8.21E-28
STAP1	-1.76028	1.98E-36	SLAMF6	-1.85518	7.66E-50
CASR	-1.06296	1.17E-07	PKDCC	-1.12099	1.66E-08
SOX30	-1.24048	2.06E-09	IL24	1.021096	1.44E-08
CP	-1.28974	9.12E-08	FCAMR	-1.96271	5.96E-16
ROS1	1.616995	9.44E-12	ACTG2	1.099941	9.29E-14
TNFRSF17	-1.76655	3.28E-21	TDRD10	-1.80243	1.99E-24
PTPRN	2.027925	1.31E-30	NPPC	-1.99997	6.33E-14
ITIH1	-1.03721	4.78E-08	PROK2	1.046779	1.82E-06
LAMC2	1.05093	8.47E-16	EOMES	-1.69943	5.42E-43
COL11A1	1.159659	3.51E-08	FCRL4	-2.77371	3.08E-38
ARSF	-1.07301	5.34E-05	TRAT1	-2.37038	1.97E-57
SEZ6	1.035689	3.90E-06	DPPA2	1.641137	0.000481
TSPAN32	-1.29024	1.58E-25	FCRL1	-2.58936	7.10E-33
EYA2	-1.02916	3.98E-08	PYHIN1	-1.72117	4.51E-48
STAG3	-1.91306	2.44E-23	CTLA4	-1.24809	1.03E-30
GAL	1.236111	1.46E-07	ICOS	-1.2413	1.61E-28
CLEC2D	-1.1748	1.09E-41	PTX3	1.049525	8.24E-08
SPTB	1.127023	4.57E-10	CXCL5	1.376567	1.39E-11

CHAT	1.290807	0.000153	RTP3	-1.09598	0.000187
PTGS2	1.159821	4.37E-11	TMEM155	-1.12973	1.77E-13
TBX21	-1.89745	1.88E-57	HHIP	1.393365	7.76E-11
GLI2	1.050599	5.94E-10	SPINK1	1.141944	1.56E-07
IPCEF1	-1.16757	1.99E-27	ENPP6	-2.10986	1.01E-26
TXK	-1.07064	3.43E-23	TLR3	-1.07294	1.06E-22
FGF4	-1.86031	1.61E-05	CSF2	1.273639	1.64E-10
ARHGAP15	-1.16567	7.00E-35	GRIK2	1.261298	7.60E-08
ICAM3	-1.19215	3.98E-22	TLX3	-1.54818	0.001276
PAK3	-1.22472	9.02E-11	SAMD3	-1.56768	9.65E-35
SPAG6	-1.96403	1.57E-19	TAGAP	-1.03568	6.84E-19
APBB1IP	-1.02512	8.78E-22	SUN3	1.614426	2.15E-15
SMC1B	-1.84205	1.61E-13	HNF4G	-1.06922	7.82E-07
CST7	-1.25657	3.61E-34	SLC30A8	1.207664	1.83E-05
FGF20	-1.43091	1.29E-05	GBX1	-3.44126	4.34E-17
P2RY10	-1.58498	2.19E-33	DCSTAMP	1.091809	2.22E-07
SP140	-1.23257	3.12E-24	CLDN3	-1.12998	7.38E-05
EPHA6	-1.34233	2.87E-07	TSHR	-1.25205	3.72E-14
RDH8	1.254889	3.26E-13	NGB	1.214391	2.32E-05
COL4A4	-1.2233	3.54E-11	DRGX	-1.71963	3.89E-10
CDH7	-1.28963	5.79E-05	BTNL9	-1.94266	4.77E-24
PTPRC	-1.23917	9.32E-24	PASD1	1.900345	1.09E-05
COL19A1	-1.754	1.30E-12	NETO1	1.620412	3.43E-17
ABCB1	-1.00414	7.01E-18	PLD4	-1.06329	9.17E-17
PTHLH	1.036307	2.00E-12	ZMAT1	-1.11665	1.59E-11
PHACTR3	1.005833	1.96E-07	PRKCB	-1.21561	1.67E-25
TESC	-1.14335	6.75E-23	MMP10	1.051544	1.72E-06
SIRPG	-2.11027	2.20E-74	ELFN2	1.174168	1.79E-08
CHGB	1.398555	2.53E-07	SCG5	1.194311	9.21E-20
LAG3	-2.02642	1.39E-59	MS4A8	-1.26388	0.001594
LYZ	-1.17247	4.33E-12	MEI1	-1.97071	1.41E-30
IL5RA	-1.21257	1.14E-11	TTC16	-1.79508	6.58E-40
TBL1Y	-1.20977	0.000537	SNX20	-1.22813	2.45E-32
GABRP	-1.36319	3.00E-07	CD3D	-2.01203	5.11E-77
MYO3A	-1.25275	1.14E-05	GNG8	-1.01041	4.16E-09
JAK2	-1.02248	3.41E-29	FAM129C	-1.94692	5.87E-35
IL12RB1	-1.59966	3.10E-48	LAIR2	-1.04728	1.33E-16
DERL3	-1.16355	1.27E-14	TMIGD2	-1.71475	4.73E-39
UPB1	-2.59528	5.20E-38	TMC8	-1.01339	3.02E-28
LGALS2	-1.20137	7.88E-24	KRT24	-2.06344	1.17E-10
TIMP3	1.131846	3.88E-14	ABCA3	-1.69307	1.16E-19
NEFH	-2.94187	1.94E-38	NLRC3	-1.4167	2.22E-53

APOBEC3H	-1.45161	8.13E-38	BATF2	-1.87941	1.51E-34
APOL4	-1.33417	6.08E-32	C11orf85	-3.28876	3.16E-34
GRAP2	-1.66252	9.08E-59	PNOC	-1.24599	1.96E-12
UPK3A	-1.09095	5.86E-11	TAP1	-1.01474	1.19E-30
IL2RB	-1.39043	7.85E-41	SFTPC	-1.41989	2.76E-06
GZMH	-2.80664	7.65E-88	GDNF	1.035487	8.48E-09
GZMB	-2.14517	4.77E-73	SOX14	-4.77962	2.06E-18
TCL1A	-2.51006	5.14E-36	LGALS9	-1.01148	2.20E-23
MMP9	1.243817	3.13E-18	ZBBX	-1.23834	0.000282
HNF4A	1.204141	1.17E-10	PARM1	-1.59167	7.37E-23
SLA2	-1.78412	7.98E-60	RSPO1	-1.50218	2.42E-19
NTSR1	1.516181	2.02E-09	CXCL10	-2.38388	4.65E-39
NKAIN4	1.313756	6.44E-11	CXCL11	-2.53299	1.12E-35
RSPO4	1.48072	1.27E-09	P2RY12	-1.29969	2.79E-15
PPP1R16B	-1.44172	1.25E-41	CXCL8	1.388742	1.13E-15
RHOXF1	-2.10074	3.84E-16	CD52	-1.01087	3.93E-17
PIM2	-1.07907	2.16E-24	MYO7B	1.159311	9.18E-09
PCSK1N	1.011983	4.06E-06	GPR25	-2.72167	5.21E-69
CD40LG	-1.90848	6.88E-37	RNF150	-1.01703	1.74E-08
KLHL4	1.283557	7.65E-10	FABP4	2.285211	1.38E-17
TAF7L	-2.40965	4.99E-24	MZB1	-1.65064	2.26E-19
NALCN	1.106433	1.05E-10	KRT4	-1.36016	2.34E-06
TNFSF13B	-1.06343	3.80E-21	KISS1	1.581907	1.66E-10
CORO1A	-1.2694	3.12E-45	BFSP2	-1.38227	2.68E-17
MT4	-1.09619	0.011827	MUC7	1.474922	0.000608
NECAB2	1.202237	3.25E-09	SMR3B	-1.94387	0.00688
SALL1	1.432047	4.04E-15	CLDN20	1.01527	4.87E-06
IL21R	-1.48487	2.87E-36	KRT38	-1.93063	1.46E-10
OCA2	-1.21458	9.91E-07	KRT13	-1.29578	4.51E-07
CALB1	1.129684	0.00027	CDK5R2	1.297422	1.03E-08
IL7	-1.02191	5.20E-21	ECEL1	-1.69818	3.60E-23
MAP4K1	-1.52895	1.21E-49	PTCRA	-1.12512	7.67E-13
CGB	1.0003	0.001482	BCL2	-1.0922	2.65E-18
CD37	-1.00392	1.30E-18	WDR87	-1.12318	0.000535
AMH	-1.07659	6.42E-08	SCG2	1.039427	9.65E-10
RETN	1.12755	2.19E-10	FOXB1	-1.3203	1.11E-13
FCER2	-2.33931	1.35E-27	MAL	-1.54214	1.58E-09
RASAL3	-1.34435	4.74E-46	CD8B	-2.19267	7.63E-56
SLC1A6	1.236147	3.35E-05	CXCR6	-1.87383	4.05E-70
ZFR2	-2.68048	1.96E-20	C1QTNF4	-1.40798	7.74E-13
TJP3	-1.10704	7.33E-10	CSDC2	1.122664	2.78E-09
SIGLEC8	-1.65067	1.43E-25	CTSW	-1.86161	7.70E-54

CD79A	-1.99318	1.43E-27	THEMIS	-1.83583	1.12E-37
NKG7	-2.46986	2.33E-86	CCL19	-2.14955	1.53E-30
SIGLEC6	-1.01308	2.24E-10	COL6A5	-1.86939	5.60E-21
HAS1	1.050342	3.99E-08	RAB37	-1.19914	3.78E-30
CACNG7	1.10322	8.85E-06	PARP15	-1.38626	1.60E-32
TFPI2	1.679492	1.51E-13	ABCD2	-1.8668	2.75E-46
SERPINE1	1.154453	2.88E-17	C1QB	-1.07604	2.13E-17
MYL7	1.273777	4.77E-07	C1QA	-1.03122	6.24E-16
AKNA	-1.02239	1.79E-45	OLR1	1.080064	7.91E-11
DOCK8	-1.02073	7.40E-26	INSM1	-1.80192	1.26E-15
PTGDS	-1.05713	2.02E-11	XCR1	-1.10207	2.49E-13
SPOCK2	-1.35352	3.06E-26	MUC13	-1.91464	4.25E-17
CCL8	-1.06552	4.98E-13	CD7	-1.45751	5.33E-42
ABI3	-1.03938	7.63E-31	TLR10	-1.97629	6.03E-47
HLF	-1.08752	5.43E-10	ZNF80	-1.86014	2.32E-29
ODAM	-1.75935	4.83E-06	WDR49	-1.94883	1.26E-15
CLNK	-1.63369	5.80E-50	P2RY14	-1.01472	3.44E-18
DDX25	-1.98581	2.42E-16	AMZ1	1.100077	1.17E-12
CRTAM	-1.82643	1.44E-46	GPR171	-1.8335	1.14E-54
FOLR3	1.11178	1.05E-06	SLC22A1	1.221416	2.06E-10
IL10RA	-1.00227	1.60E-21	TBC1D10C	-1.73969	2.49E-61
CD5	-1.54625	1.30E-41	GAPT	-1.26911	3.58E-22
C11orf21	-1.59809	7.47E-38	KLHL38	1.139282	2.17E-08
POU2AF1	-1.47243	4.64E-21	ZNF683	-2.52277	2.46E-66
CD69	-1.0674	9.81E-18	ACBD7	-1.16327	2.41E-10
BIN2	-1.0253	1.89E-24	MAGEB6	1.933532	1.57E-06
SYT10	-1.33617	0.000194	C8orf4	-1.22761	2.52E-14
PRR4	-1.08492	2.55E-06	C8G	-1.28256	1.06E-17
CUX2	-1.12035	9.16E-09	DEFB4B	-1.48337	0.000178
ART4	-1.47289	2.05E-14	KCNA3	-1.71765	1.58E-26
IFNG	-2.76307	6.02E-74	KCNA2	-2.94543	2.71E-38
AICDA	-1.20916	1.45E-08	SAMD9L	-1.00599	1.77E-19
KLRB1	-1.9283	5.15E-64	CD19	-2.3549	1.03E-41
ULBP1	1.04746	2.83E-09	KCNJ10	-1.97162	1.31E-29
IL17A	-1.48041	1.29E-10	SPINK6	-1.03761	0.000604
CCR6	-1.15514	3.64E-15	ZC3H12D	-1.16713	4.09E-32
C6orf118	-1.28338	1.28E-05	GLDC	-1.43254	4.05E-11
C7	-1.28396	2.27E-07	CD28	-1.08785	2.87E-19
GZMK	-2.29358	2.21E-53	DYNAP	-1.79076	4.42E-08
ITK	-1.59406	4.52E-41	C5orf46	1.069201	1.33E-08
IL12B	-1.67536	9.28E-31	C14orf39	-1.02961	7.24E-06
SERPINI2	-2.49838	5.71E-16	ZFP42	2.117325	5.72E-11

MORC1	-1.02268	0.018794	FAM133A	1.455948	0.000155
IL1A	1.150538	1.23E-11	GIMAP7	-1.36125	1.52E-41
ZAP70	-1.79094	7.98E-60	HLA-DQB1	-1.33554	1.57E-27
POMC	1.046179	5.59E-07	CIITA	-1.80682	7.23E-59
TACR1	-1.08557	1.33E-09	FAM216B	-1.61983	2.01E-07
FN1	1.36123	4.18E-16	AKAP5	-1.09616	6.67E-33
GNLY	-1.14176	6.10E-14	GPBAR1	-1.02297	4.53E-24
IL18RAP	-1.37936	3.94E-33	1-Sep	-1.56109	9.18E-67
NPHS2	-1.34774	0.007856	FUT7	-1.63355	1.48E-44
CD2	-1.91732	6.72E-72	SSTR2	-1.12302	4.10E-18
SLAMF1	-1.54257	1.08E-48	PRF1	-1.73683	1.10E-53
CD48	-1.4879	4.36E-38	FCRL6	-1.60595	2.21E-31
PLA2G2D	-2.29804	1.26E-26	MUC16	-1.02596	5.45E-06
GBP1	-1.36339	1.76E-29	C4orf50	-1.29533	2.90E-15
CR2	-2.09241	2.35E-19	FDCSP	-2.7112	2.16E-19
FMO6P	-1.27586	6.11E-06	P2RY13	-1.28767	3.54E-23
FASLG	-2.60717	1.39E-86	TIGIT	-1.65403	6.40E-55
MMP8	2.06913	1.13E-23	UBA7	-1.13212	2.90E-45
ZNF541	-1.92955	1.57E-15	NLRP10	1.061584	1.12E-05
LRMP	-1.08748	1.43E-14	SPNS3	-1.09196	2.98E-23
MYB	-1.01084	5.35E-09	KRTAP11-1	1.117694	0.008463
GHRH	1.053883	0.034372	HS3ST4	-2.26338	1.56E-14
NKX2-3	-1.87287	1.01E-11	KCNB2	-1.53341	3.18E-08
CD274	-1.07182	1.31E-14	LCK	-1.26305	1.48E-38
PRB2	1.696901	2.13E-09	MCEMP1	1.230598	1.51E-16
BCL2L14	-1.83327	2.48E-41	PCP4	-1.36443	0.000557
CCR2	-1.2902	2.65E-18	OR51B4	1.240721	0.014407
TMEM156	-1.24235	4.16E-23	CCDC60	-1.41447	5.13E-06
FLT3	-1.36567	8.76E-28	GRIN2A	-1.65289	9.73E-12
SASH3	-1.34681	1.67E-39	FAM46C	-1.33838	5.98E-21
LAX1	-1.66504	1.41E-31	PRR32	1.055095	0.00287
CD244	-1.38396	1.22E-33	OPCML	1.464654	8.25E-13
LY9	-1.70886	1.12E-40	B3GALT5	-1.44066	1.24E-08
INHBA	1.074335	2.48E-13	CCR4	-1.1586	9.21E-16
CDKN2C	-1.08809	4.20E-20	MAATS1	-1.52246	3.01E-12
ARHGAP9	-1.2603	6.94E-43	PNMA3	-1.19163	6.77E-09
HOXC12	1.058427	0.011682	FAM3B	-1.71971	4.13E-12
DBH	-1.73417	1.12E-33	SCN5A	1.035447	1.34E-07
COL10A1	1.249685	7.96E-12	C1orf64	-1.01469	0.003451
MAGEA9B	1.728655	0.016545	SH2D1A	-1.98062	2.32E-60
G0S2	1.656039	3.06E-27	TMPRSS2	-1.03344	6.17E-06
ZNF831	-1.8991	1.27E-43	CNTN2	-1.02095	4.26E-07

C20orf85	-2.54979	4.57E-10	CLECL1	-1.35136	6.36E-27
ZBP1	-1.5308	1.41E-31	KRTAP19-1	1.008973	0.008694
MAGEA10	1.094894	0.023307	CLDN6	1.543821	3.35E-13
TCP11	-1.42403	7.19E-08	LCN12	-1.0177	5.96E-08
DNAH8	-1.00653	7.28E-09	SV2B	-1.29152	9.98E-11
GPR18	-2.0114	7.31E-63	DLK1	-1.15116	0.002414
KIF25	-1.61115	1.09E-16	DBX2	-1.55606	3.96E-06
IRF1	-1.27183	3.62E-51	PDIA2	1.438958	1.52E-08
TGM3	-1.38895	1.83E-06	ADARB2	-1.68096	4.15E-15
PAX1	-2.74932	1.61E-28	IKZF1	-1.48484	7.25E-40
NKX2-4	-2.06963	0.000445	CCIN	1.123303	1.48E-14
BPIFB1	-1.01144	0.014911	ZPBP2	-5.27917	9.88E-37
IGFLR1	-1.08853	6.87E-33	CYP4Z1	-1.66789	1.03E-12
HCST	-1.08456	1.07E-20	BTLA	-1.74005	4.60E-44
KRT36	-2.19167	2.51E-23	GABRA5	1.277521	0.001285
CCR7	-1.05844	1.36E-14	CYP4X1	-1.54048	4.23E-17
STATH	-1.10882	0.004954	FCAR	1.051308	1.38E-09
CTAG2	2.309425	4.41E-05	CCDC42B	-1.0353	4.60E-15
RGS13	-1.22253	8.06E-18	CXCR3	-2.20854	1.20E-69
SYNGR3	-1.06203	1.55E-10	FGF3	-1.49561	0.020988
FGL2	-1.29014	1.50E-27	FOXD3	-1.2339	2.43E-10
VPREB3	-1.41909	4.06E-17	DCC	-1.20847	2.96E-11
MGAT3	-1.63183	2.65E-18	PLEKHG7	-1.29838	4.89E-12
APOL3	-1.45254	3.34E-46	TMEM8C	1.043503	0.004266
IGLL1	-1.7436	2.66E-13	CXorf67	1.347742	0.008346
WDFY4	-1.22498	1.83E-20	LIN28B	1.233767	0.01322
FOXA1	-1.16691	1.08E-08	ASCL4	-2.04068	9.46E-14
PRRG3	-1.07561	1.33E-06	TTC24	-1.59397	5.66E-29
CNN1	1.104828	1.38E-15	C1orf168	-1.39756	5.51E-09
DPP6	1.420537	2.48E-12	RTP5	-1.98913	1.45E-25
GFAP	1.100912	7.87E-08	NWD1	-1.2253	3.24E-12
CCL25	-1.49314	2.55E-12	IL17REL	-3.63923	1.11E-57
IDO1	-2.96865	1.37E-70	RUFY4	-1.42794	4.92E-23
C19orf57	-1.02571	3.80E-18	PDCD1	-2.15352	3.82E-86
MATN3	1.168686	2.58E-16	SELL	-1.27917	2.53E-21
FCRLA	-1.30379	5.38E-16	SERPINA5	1.291653	2.48E-13
TRIM22	-1.11818	1.36E-24	IDO2	-1.77103	2.80E-35
DDC	1.186323	8.16E-07	VWC2	-1.17365	1.14E-06
IGJ	-1.78099	1.26E-20	BCL2L15	-1.11347	1.13E-09
CLEC10A	-1.44174	8.03E-28	HMX2	-1.07609	0.003113
FCRL2	-2.11909	2.01E-26	FAM26F	-1.41504	4.45E-31
ACY3	-1.02681	1.20E-16	CNR2	-2.16001	3.19E-39

KANK4	1.186507	6.26E-09	AADACL3	-1.22188	0.002924
RARRES3	-1.51944	1.18E-34	KIR2DL4	-1.85742	1.57E-36
GIMAP4	-1.06988	5.72E-30	CGB5	1.588877	2.85E-10
TEX15	-1.22922	0.000857	ANKRD34B	-1.03241	0.000212
CMPK2	-1.01429	1.26E-15	NUGGC	-1.72949	3.23E-32
KLRD1	-1.51873	3.93E-34	FAM150B	-1.0041	8.55E-07
KLRC1	-1.52712	1.28E-29	NCR1	-1.793	5.39E-42
CLDN10	-1.54202	3.02E-08	SYCP2	-1.61845	3.05E-14
PRPH	1.245009	8.85E-12	SPOCK3	1.186601	7.23E-07
TESPA1	-1.34724	2.45E-35	HLA-DRB1	-1.36172	1.59E-31
GPR55	-1.25782	2.79E-26	CTSE	-1.58436	3.08E-13
RTP4	-1.1436	2.88E-24	GIMAP5	-1.22927	9.40E-29
TBR1	-1.5388	4.06E-08	WNK3	-1.01908	2.58E-08
BLK	-1.34245	1.10E-11	SLC30A10	1.169875	7.90E-07
SIT1	-1.71376	2.33E-51	TLR7	-1.18072	2.01E-17
CD72	-1.06999	9.01E-19	HSH2D	-1.15838	2.84E-23
IRF4	-1.5815	1.86E-34	HLA-DQA1	-1.25339	3.25E-24
FGFBP2	1.041964	0.000197	CHRNG	1.041006	9.26E-05
IL18BP	-1.27515	5.33E-42	FAM163B	-2.24655	3.95E-27
IFI44L	-1.05668	1.35E-12	ACSL5	-1.14777	8.13E-27
FGF5	1.965534	5.38E-19	IL27	-1.09937	7.78E-14
CXCL9	-2.4772	1.42E-51	CYP2F1	-1.32442	3.76E-06
KLRG1	-1.09978	4.48E-21	SPN	-1.2318	3.27E-26
CD27	-1.89847	8.51E-55	C5orf56	-1.38891	8.14E-55
COL2A1	1.074194	3.82E-09	GZMM	-2.04853	1.25E-59
ITGB7	-1.05064	8.30E-24	MFAP5	1.140948	1.12E-10
TTC6	-1.05087	2.88E-05	MPEG1	-1.11798	4.80E-21
WARS	-1.64856	2.53E-37	KLHL14	-1.12088	1.01E-09
IRF8	-1.39	7.69E-35	CR1L	-1.68965	9.80E-23
SKAP1	-1.16148	1.67E-19	CLEC9A	-1.40227	1.17E-23
SECTM1	-1.20657	1.26E-29	KEL	-1.7909	2.86E-16
CBLN2	-2.30391	3.54E-19	FCGR1B	-1.09176	8.54E-16
IL19	-1.27022	1.30E-09	CLEC4C	-1.6574	3.71E-15
SIGLEC10	-1.11382	2.21E-20	BPIFA1	-1.03661	0.045079
C1orf94	1.252856	5.00E-06	HLA-DRB5	-1.30896	1.81E-24
DMRTA2	-1.3031	2.45E-05	C1orf228	-1.13564	5.00E-23
GPA33	-1.51406	1.64E-23	SPANXC	2.115174	3.57E-06
XCL2	-1.28949	8.90E-30	APCDD1L	1.34852	1.38E-16
MAEL	1.040242	0.000422	CD247	-1.61359	1.03E-51
FCRL5	-1.66714	3.33E-18	CD3E	-1.82499	6.79E-70
HHIPL2	1.105879	1.70E-07	L1CAM	1.263502	1.43E-12
CRNN	-1.4997	3.04E-06	RORB	-2.66626	1.02E-38

C1QL2	-1.06228	0.009527	OOEP	-1.1834	7.39E-06
4-Mar	1.288605	8.37E-18	TCEAL5	1.076239	5.88E-06
GPR128	-1.46834	1.56E-06	CLPSL1	-1.23856	0.00168
GZMA	-2.05031	2.22E-53	CXorf65	-1.47711	1.74E-24
TIMD4	-1.59964	1.26E-15	HLA-DOA	-1.47042	1.64E-29
SPINK7	-1.45168	5.18E-07	HLA-DMA	-1.39331	2.23E-41
FGD2	-1.00594	1.48E-28	HLA-DRA	-1.33823	7.84E-34
SCML4	-2.17644	7.01E-57	SP5	-1.55902	6.60E-17
RAB19	-1.30165	4.59E-20	NCR3	-1.41966	3.48E-25
GPR174	-2.06524	1.62E-40	MUC21	-1.6699	3.07E-07
IL2RG	-1.41934	9.88E-37	RANBP17	-1.12808	1.58E-08
NXF3	1.227105	8.52E-10	PSG5	1.168645	6.43E-07
ARHGAP36	1.037281	0.000131	PKHD1L1	-1.48678	2.07E-15
FATE1	-1.15715	1.85E-11	SLFN12L	-1.16686	1.52E-40
DOK2	-1.00233	7.77E-24	PSMB10	-1.05548	2.19E-40
CDKN2A	-1.07183	1.16E-06	EXOC3L4	-1.52377	1.47E-18
CRB2	-2.25504	9.23E-25	ARRDC5	-1.16087	3.43E-20
PLEKHS1	-1.05911	2.90E-05	KLRC2	-1.82522	2.85E-25
KLHL35	-1.02619	1.65E-08	CLEC6A	-1.47343	1.72E-16
DRD2	1.438012	5.90E-16	COL6A6	-1.15729	2.11E-11
ZP1	1.326684	3.15E-13	XKR4	-1.9998	9.09E-18
KLRF1	-1.06605	1.88E-13	KRTAP2-3	1.076854	4.37E-05
FCGR1A	-1.0007	9.17E-15	CGB8	1.102918	3.62E-05
CD226	-1.40456	1.09E-40	GIMAP1	-1.20056	4.36E-36
RAD9B	-1.11354	1.54E-20	KLRK1	-1.55455	2.21E-31
KCTD14	-1.01981	2.78E-10	UBD	-2.74121	7.46E-65
TUBA3E	1.051468	0.000104	HBE1	1.331313	0.000775
SPOCK1	1.117557	9.50E-12	MAGEB1	1.913908	0.021973
CNTNAP4	1.038673	0.000156	C12orf74	-1.32501	4.09E-09
JAKMIP1	-2.06694	1.82E-71	MUC5AC	-1.07684	0.001135
BANK1	-1.08446	1.19E-15	LINGO3	-1.07125	8.25E-13
CLGN	-1.73394	2.70E-14	HMSD	-1.23252	3.14E-12
FEZF2	-3.20155	1.16E-06	HLA-DPB1	-1.30785	5.27E-34
CD96	-1.72494	2.36E-71	ENSG00000226321	-1.14968	1.72E-07
FRMD1	-1.36063	2.41E-07	LTA	-1.53872	6.46E-42
CD8A	-2.52321	7.72E-93	SPANXB2	2.082843	1.02E-06
FAM92B	-1.37692	1.49E-10	LTB	-1.2383	1.02E-25
JPH3	1.668654	1.31E-17	PATL2	-1.05667	3.14E-26
GPR15	-1.54793	4.25E-15	HLA-DPA1	-1.40332	9.98E-34
GBP5	-2.18103	3.43E-43	HLA-DQB2	-1.11414	8.05E-15
TMPRSS15	1.305821	3.47E-05	PAGE2	1.188574	0.044777
MAGEC1	1.979162	0.000453	HLA-DQA2	-1.4264	5.58E-21

GRIA1	1.496695	5.52E-11	APOBEC3G	-1.10277	1.45E-26
XAGE2B	1.602799	0.008207	LILRA4	-1.88861	5.89E-31
TTN	1.142292	4.36E-06	PSMB9	-1.16126	1.49E-29
ADCY8	1.026819	0.000881	KIR3DL2	-1.88265	3.48E-19
SLA	-1.04029	4.99E-25	TNFRSF13B	-2.43069	1.56E-38
RAB39B	-1.02989	1.84E-13	HLA-DOB	-1.01789	6.15E-26
BATF	-1.22925	6.32E-33	ARHGDIG	1.346748	9.06E-10
CXCL13	-1.46421	3.68E-26	HLA-DMB	-1.32187	6.97E-33
NAA11	2.093232	1.69E-06	APOBEC3D	-1.16714	9.28E-30
MS4A1	-2.99136	1.70E-45	ADH1C	-2.13398	1.85E-13
ITGAD	-1.09211	5.14E-14	RTL1	1.636931	1.37E-06
LHFPL4	-2.28481	8.58E-14	IGLL5	-1.72446	2.70E-17
GHRL	-1.02185	1.21E-19	KLRC4-KLRK1	-2.19852	6.21E-43
SHCBP1L	-3.67204	1.33E-19	TIFAB	-1.60681	7.87E-31
TMSB15A	-1.27094	6.33E-11	SALL3	-1.99653	8.49E-08
HOXB13	-1.08416	0.000318	SLC5A8	-1.19131	0.000796
SIM2	-1.65262	1.88E-23	MGAM	1.130524	3.77E-12
IRX6	-1.40495	4.23E-09	RP11-812E19.9	-1.91813	1.65E-12
CES5A	1.205867	3.78E-06	TMEM178B	-1.3852	4.32E-10
TGM7	-1.19743	1.83E-05	CCER2	-1.00319	1.71E-06
RGL4	-1.04097	3.61E-30	ENSG00000263264	-1.18321	1.07E-13
GPR114	-1.50862	6.20E-33	MSMB	-1.81556	1.48E-13
TNFRSF13C	-1.03256	4.95E-14	C1orf186	-1.10837	1.17E-11
TFF3	-1.28143	1.42E-08	CT45A1	1.348484	0.033059
TMPRSS3	-1.22565	4.73E-13	SPIB	-3.283	7.07E-75
UBASH3A	-1.97881	6.01E-71	ZBTB8B	-1.24641	9.63E-08
AIRE	-1.27105	2.05E-10	CCL4	-1.04219	5.45E-19
LCN1	1.568291	2.28E-12	SSTR3	-1.50023	3.22E-29

Table S5: KEGG enrichment analysis of 160 genes

ID	Description	GeneRatio	BgRatio	pvalue	p.adjust	qvalue	geneID
hsa05340	Primary immunodeficiency	11/70	37/7911	8.19E-15	1.20E-12	1.03E-12	CD19/CD79A/TNFRSF13C/CD3D/TNFRSF13B/CD3E/ZAP70/ICOS/CD40LG/AICDA/LCK
hsa04060	Cytokine-cytokine receptor interaction	15/70	294/7911	2.84E-08	2.08E-06	1.78E-06	INHBA/TNFRSF17/CD27/TNFRSF13C/IL1A/CXCL5/TNFRSF13B/CXCL8/IL12B/CCR4/CD40LG/XCL2/CXCR6/CCR7/CXCR3
hsa04672	Intestinal immune network for IgA production	7/70	49/7911	2.00E-07	9.73E-06	8.35E-06	TNFRSF17/TNFRSF13C/TNFRSF13B/ITGB7/ICOS/CD40LG/AICDA
hsa04660	T cell receptor signaling pathway	7/70	103/7911	3.16E-05	0.001154	0.00099	CD3D/CD3E/ZAP70/ICOS/CD40LG/LCK/PAK3
hsa05144	Malaria	5/70	49/7911	6.62E-05	0.001932	0.001658	KLRB1/CXCL8/CD40LG/KLRC4-KLRK1/KLRK1
hsa04658	Th1 and Th2 cell differentiation	6/70	92/7911	0.000151	0.003682	0.003159	CD3D/CD3E/IL12B/ZAP70/TBX21/LCK
hsa04640	Hematopoietic cell lineage	6/70	97/7911	0.000203	0.003893	0.00334	CD19/IL1A/CD3D/MS4A1/CD3E/CD7
hsa04061	Viral protein interaction with cytokine and cytokine receptor	6/70	100/7911	0.000239	0.003893	0.00334	CXCL5/CXCL8/CCR4/XCL2/CCR7/CXCR3
hsa04062	Chemokine signaling pathway	8/70	189/7911	0.000243	0.003893	0.00334	CXCL5/CXCL8/CCR4/GNG8/XCL2/CXCR6/CCR7/CXCR3
hsa04064	NF-kappa B signaling pathway	6/70	102/7911	0.000267	0.003893	0.00334	TNFRSF13C/CXCL8/ZAP70/CD40LG/BCL2/LCK

hsa04650	Natural killer cell mediated cytotoxicity	6/70	131/7911	0.001012	0.013335	0.011441	ZAP70/CD244/NCR3/LCK/KLRC4-KLRK1/KLRK1
hsa05235	PD-L1 expression and PD-1 checkpoint pathway in cancer	5/70	89/7911	0.001096	0.013335	0.011441	CD3D/BATF/CD3E/ZAP70/LCK
hsa05162	Measles	6/70	138/7911	0.001326	0.014892	0.012776	IL1A/CD3D/CD3E/IL12B/BCL2/SLAMF1
hsa04514	Cell adhesion molecules (CAMs)	6/70	146/7911	0.00177	0.01846	0.015838	L1CAM/ITGB7/CLDN10/ICOS/CD40LG/TIGIT
hsa04659	Th17 cell differentiation	5/70	107/7911	0.002483	0.024172	0.020739	CD3D/CD3E/ZAP70/TBX21/LCK
hsa05133	Pertussis	4/70	76/7911	0.004467	0.040764	0.034975	IL1A/CXCL5/CXCL8/IL12B
hsa00565	Ether lipid metabolism	3/70	47/7911	0.008145	0.069955	0.060019	PLD4/ENPP6/PLA2G2D
hsa05142	Chagas disease (American trypanosomiasis)	4/70	102/7911	0.012468	0.101128	0.086765	CD3D/CXCL8/CD3E/IL12B
hsa05321	Inflammatory bowel disease (IBD)	3/70	65/7911	0.019605	0.150646	0.129249	IL1A/IL12B/TBX21
hsa05202	Transcriptional misregulation in cancer	5/70	186/7911	0.024064	0.175664	0.150714	CXCL8/ITGB7/CDKN2C/PTCRA/TMPRSS2
hsa04662	B cell receptor signaling pathway	3/70	82/7911	0.035762	0.248629	0.213315	CD19/CD79A/CD79B
hsa05170	Human immunodeficiency virus 1 infection	5/70	212/7911	0.039145	0.258251	0.221571	CD3D/CD3E/GNG8/BCL2/PAK3
hsa05143	African trypanosomiasis	2/70	37/7911	0.04213	0.258251	0.221571	IL12B/IDO2
hsa05166	Human T-cell leukemia virus 1 infection	5/70	219/7911	0.044011	0.258251	0.221571	TNFRSF13C/CD3D/CD3E/CDKN2C/LCK
hsa05330	Allograft rejection	2/70	38/7911	0.044221	0.258251	0.221571	IL12B/CD40LG
hsa04657	IL-17 signaling pathway	3/70	93/7911	0.048956	0.264728	0.227128	CXCL5/DEFB4B/CXCL8
hsa05323	Rheumatoid arthritis	3/70	93/7911	0.048956	0.264728	0.227128	IL1A/CXCL5/CXCL8

Table S6: GO enrichment analysis of 160 genes

ONTOLOGY	ID	Description	GeneRatio	BgRatio	pvalue	p.adjust	qvalue	geneID
BP	GO:0002250	adaptive immune response	29/141	381/16240	1.90E-19	3.91E-16	3.30E-16	TNFRSF17/CD27/CD79A/IGLL5/TNFRSF13C/FCAMR/CD3D/BATF/CD79B/TNFRSF13B/CD3E/TXK/IL12B/CD7/LY9/SASH3/FCRL
BP	GO:0042110	T cell activation	29/141	456/16240	2.50E-17	2.58E-14	2.18E-14	CD27/GPR18/TNFRSF13C/BLK/CD3D/NLRC3/BATF/CD3E/TXK/IL12B/CD7/LY9/SASH3/SLAMF6/ZAP70/ICOS/CD40LG/
BP	GO:0030098	lymphocyte differentiation	25/141	328/16240	7.49E-17	5.15E-14	4.34E-14	INHBA/IKZF3/CD27/CD79A/GPR18/BLK/CD3D/BATF/CD79B/CD3E/TXK/IL12B/LY9/SASH3/SLAMF6/
BP	GO:0051249	regulation of lymphocyte activation	27/141	419/16240	2.62E-16	1.35E-13	1.14E-13	INHBA/IKZF3/CD27/TBC1D10C/IGLL5/TNFRSF13C/CD3D/TNFRSF13B/CD3E/IL12B/SASH3/ZAP70/ICOS/CD40LG/BCL2/
BP	GO:0002694	regulation of leukocyte activation	28/141	486/16240	1.21E-15	4.98E-13	4.20E-13	INHBA/IKZF3/CD27/TBC1D10C/IGLL5/TNFRSF13C/CD3D/TNFRSF13B/CD3E/IL12B/SASH3/ZAP70/CNR2/ICOS/CD40LG/BC
BP	GO:0002521	leukocyte differentiation	26/141	480/16240	6.11E-14	2.10E-11	1.77E-11	INHBA/IKZF3/CD27/CD79A/GPR18/BLK/CD3D/BATF/CD79B/CD3E/TXK/IL12B/GPR55/LY9/SASH3/SLAM
BP	GO:0042113	B cell activation	19/141	241/16240	2.82E-13	8.30E-11	6.99E-11	INHBA/IKZF3/CD27/CD79A/TBC1D10C/IGLL5/TNFRSF13C/BATF/MS4A1/CD79B/TNFRSF1
BP	GO:0030217	T cell differentiation	18/141	223/16240	8.32E-13	2.14E-10	1.81E-10	CD27/GPR18/BLK/CD3D/BATF/CD3E/TXK/IL12B/LY9/SASH3/SLAMF6/ZAP
BP	GO:0051251	positive regulation of lymphocyte activation	19/141	279/16240	3.82E-12	8.76E-10	7.38E-10	CD27/IGLL5/TNFRSF13C/CD3D/CD3E/IL12B/SASH3/ZAP70/ICOS/CD40LG/BCL2/TBX2
BP	GO:0045058	T cell selection	10/141	45/16240	4.34E-12	8.95E-10	7.54E-10	CD3D/BATF/CD3E/IL12B/LY9/SLAMF6/ZAP70/
CC	GO:0098552	side of membrane	25/147	470/17107	2.54E-13	4.45E-11	4.15E-11	CD19/CD27/CD79A/IGLL5/TNFRSF13C/BLK/MS4A1/CD79B/CD3E/TXK/CCR4/GNG8/ZAP70/AKAP5/CNR2/C
CC	GO:0009897	external side of plasma membrane	17/147	250/17107	5.08E-11	4.45E-09	4.15E-09	CD19/CD27/CD79A/IGLL5/TNFRSF13C/MS4A1/CD79B/CD3E/CCR4/CD244/SERPINA
CC	GO:0031234	extrinsic component of cytoplasmic side of plasma membrane	7/147	109/17107	4.30E-05	0.002508	0.002338	BLK/TXK/GNG8/ZAP70/CNR2/LCK
CC	GO:0009898	cytoplasmic side of plasma membrane	8/147	175/17107	0.000136	0.005956	0.005553	BLK/TXK/GNG8/ZAP70/AKAP5/CNR2/
CC	GO:0098562	cytoplasmic side of membrane	8/147	198/17107	0.000316	0.011056	0.010308	BLK/TXK/GNG8/ZAP70/AKAP5/CNR2/
CC	GO:0019814	immunoglobulin complex	3/147	19/17107	0.000545	0.013613	0.012692	CD79A/IGLL5/CD79B
CC	GO:0042101	T cell receptor complex	3/147	19/17107	0.000545	0.013613	0.012692	CD3D/CD3E/ZAP70
CC	GO:0019897	extrinsic component of plasma membrane	7/147	169/17107	0.000648	0.014182	0.013222	BLK/TXK/GNG8/ZAP70/CNR2/LCK
CC	GO:0001669	acrosomal vesicle	5/147	106/17107	0.002212	0.043012	0.040101	SPINK7/SPINK1/SERPINA5/SV2B/
CC	GO:0001772	immunological synapse	3/147	33/17107	0.002808	0.04914	0.045815	CD3E/ZAP70/LCK

MF	GO:0004715	non-membrane spanning protein tyrosine kinase activity	5/130	50/16239	4.83E-05	0.004653	0.004163	BLK/TXK/ZAP70/LCK/SLA2
MF	GO:0001637	G-protein coupled chemoattractant receptor activity	4/130	26/16239	5.11E-05	0.004653	0.004163	CCR4/CXCR6/CCR7/CXCR3
MF	GO:0004950	chemokine receptor activity	4/130	26/16239	5.11E-05	0.004653	0.004163	CCR4/CXCR6/CCR7/CXCR3
MF	GO:0005125	cytokine activity	8/130	215/16239	0.000338	0.023063	0.020631	INHBA/FAM3B/IL1A/CXCL5/CXCL8/IL12B/
MF	GO:0042288	MHC class I protein binding	3/130	19/16239	0.000442	0.024148	0.021602	CD244/KLRC4-KLRK1/KLRK[illegible]
MF	GO:0004867	serine-type endopeptidase inhibitor activity	5/130	88/16239	0.000701	0.030746	0.027503	SPINK7/TFPI2/SPINK6/SPINK1/SER
MF	GO:0004896	cytokine receptor activity	5/130	91/16239	0.000816	0.030746	0.027503	IL12B/CCR4/CXCR6/CCR7/CXC
MF	GO:0030246	carbohydrate binding	8/130	250/16239	0.000912	0.030746	0.027503	PTX3/KLRB1/OLR1/LGALS2/SIGLEC6/KLRG1/KL
MF	GO:0019956	chemokine binding	3/130	25/16239	0.001014	0.030746	0.027503	CXCR6/CCR7/CXCR3
MF	GO:0042287	MHC protein binding	3/130	29/16239	0.001573	0.042947	0.038418	CD244/KLRC4-KLRK1/KLRK[illegible]

Table S7: TMEscore details for Risk-H and Risk-L groups

univariant_HR.gName	univariant_HR.Pvalue	univariant_HR.HR	univariant_HR.Low.95.CI.	univariant_HR.High.95.CI.	univariant_HR.Logrank	univariant_HR.Sctest	univariant_HR.Waldtest	univariant_HR.fpkm_median	univariant_HR.fpkm_mean
signature G3 PC1	7.83E-05	0.664954	0.543068	0.814197	2.27E-06	6.85E-05	7.83E-05	-0.33656	-7.76E-18
signature G2 PC1	0.000142	0.713356	0.599424	0.848944	1.84E-05	0.000115	0.000142	-0.35065	1.35E-17
signature G1 PC1	0.000404	0.742714	0.629863	0.875785	0.000114	0.000357	0.000404	-0.31711	-2.08E-17

Table S8: The Cox multivariate regression analysis results of PC1 in three groups of signature Gs

TMEscore.A0_Samples	TMEscore.A1_OS	TMEscore.Status	TMEscore.RiskScore	TMEscore.RiskType	TMEscore.A0_Samples	TMEscore.A1_OS	TMEscore.Status	TMEscore.RiskScore	TMEscore.RiskType
TCGA-CV-A45V	32	1	0.12118	Risk-L	TCGA-CN-A49C	645	0	-0.06434	Risk-L
TCGA-MT-A67D	56	0	0.02262	Risk-L	TCGA-QK-A652	645	0	-0.10744	Risk-L
TCGA-CV-7102	56	1	0.239033	Risk-H	TCGA-CV-A6JY	646	0	0.249486	Risk-H
TCGA-P3-A6T4	62	1	0.189906	Risk-H	TCGA-QK-A6IH	653	0	0.32591	Risk-H
TCGA-CV-7255	64	1	0.295398	Risk-H	TCGA-CQ-5325	654	1	-0.18754	Risk-L
TCGA-CV-6934	65	1	-0.22088	Risk-L	TCGA-F7-8489	658	0	-0.09138	Risk-L
TCGA-BA-A6DG	69	1	0.209283	Risk-H	TCGA-CV-7177	663	1	0.222413	Risk-H
TCGA-CV-6961	76	1	0.076866	Risk-L	TCGA-CV-6939	666	1	-0.59153	Risk-L
TCGA-D6-6824	77	0	-0.35523	Risk-L	TCGA-HD-7831	667	0	0.182408	Risk-H
TCGA-CN-A642	82	1	0.100243	Risk-L	TCGA-HD-8314	670	0	-0.4449	Risk-L
TCGA-UF-A71A	86	1	0.279577	Risk-H	TCGA-CV-7440	675	1	0.154331	Risk-H
TCGA-CQ-5326	89	1	0.239241	Risk-H	TCGA-CN-A63V	679	0	0.229565	Risk-H
TCGA-UF-A7JV	90	1	0.179659	Risk-H	TCGA-UF-A7JS	680	1	0.286553	Risk-H
TCGA-F7-A50I	92	0	0.148908	Risk-H	TCGA-CV-A6K1	685	0	0.332705	Risk-H
TCGA-CR-7371	94	1	0.031198	Risk-L	TCGA-D6-A6EN	687	0	0.443255	Risk-H
TCGA-T3-A92N	95	1	0.219093	Risk-H	TCGA-CR-6491	693	0	0.292825	Risk-H
TCGA-4P-AA8J	102	0	0.192309	Risk-H	TCGA-CV-7415	695	1	0.280775	Risk-H
TCGA-CR-7370	105	0	-0.00947	Risk-L	TCGA-HD-8635	695	0	0.116884	Risk-L
TCGA-CV-7429	107	1	0.186686	Risk-H	TCGA-CV-5971	701	0	-0.14402	Risk-L
TCGA-BA-6871	108	1	0.00858	Risk-L	TCGA-D6-6823	701	0	0.170086	Risk-H
TCGA-CV-A6JU	110	0	0.345196	Risk-H	TCGA-QK-A6IF	704	0	-3.91559	Risk-L

TCGA-HD-A6HZ	111	0	-0.14586	Risk-L	TCGA-CQ-A4C9	707	0	0.186766	Risk-H
TCGA-CN-6995	112	1	0.291499	Risk-H	TCGA-D6-A74Q	710	0	0.11805	Risk-L
TCGA-BA-6873	122	0	0.15817	Risk-H	TCGA-CN-A6UY	713	0	-0.13484	Risk-L
TCGA-CV-6962	126	1	0.289996	Risk-H	TCGA-CV-A6JZ	714	0	-0.21818	Risk-L
TCGA-CQ-5334	129	1	0.193327	Risk-H	TCGA-CN-A499	717	0	-0.36958	Risk-L
TCGA-CQ-6227	129	1	0.166147	Risk-H	TCGA-BA-5151	722	0	0.10455	Risk-L
TCGA-HD-A634	130	1	-0.08154	Risk-L	TCGA-BA-5556	725	0	-0.34614	Risk-L
TCGA-BB-4227	134	0	0.261967	Risk-H	TCGA-CN-6013	727	1	-0.12009	Risk-L
TCGA-CN-4726	142	1	0.215571	Risk-H	TCGA-UF-A7JD	739	1	0.067658	Risk-L
TCGA-CV-6938	144	1	0.13093	Risk-L	TCGA-CN-A6V3	742	0	0.004048	Risk-L
TCGA-CV-7236	144	1	0.137031	Risk-L	TCGA-CR-7397	754	0	0.069029	Risk-L
TCGA-BB-4225	146	0	-0.11716	Risk-L	TCGA-CR-7372	759	0	0.116089	Risk-L
TCGA-CV-7252	151	1	0.226201	Risk-H	TCGA-F7-A61V	759	0	0.19003	Risk-H
TCGA-CR-7398	156	0	-0.10952	Risk-L	TCGA-D6-8568	759	0	0.086411	Risk-L
TCGA-CV-7101	160	1	0.149845	Risk-H	TCGA-D6-A6EO	759	0	0.048514	Risk-L
TCGA-CV-6936	166	1	0.164611	Risk-H	TCGA-CV-7416	763	1	0.230195	Risk-H
TCGA-QK-A8Z8	171	1	0.091019	Risk-L	TCGA-D6-8569	770	0	0.129243	Risk-L
TCGA-BA-A6DD	173	1	0.337754	Risk-H	TCGA-D6-6516	773	0	0.165045	Risk-H
TCGA-CR-7399	181	0	-0.17046	Risk-L	TCGA-CN-A498	773	1	0.17597	Risk-H
TCGA-CV-A6JD	182	1	0.166679	Risk-H	TCGA-BB-A5HU	782	0	0.183036	Risk-H
TCGA-CR-6478	183	1	-0.17475	Risk-L	TCGA-HD-7754	783	0	-0.09829	Risk-L
TCGA-CV-6952	185	1	0.200898	Risk-H	TCGA-CV-7418	789	1	-0.15385	Risk-L
TCGA-BB-4217	187	0	-3.49154	Risk-L	TCGA-CR-7382	796	0	-0.80094	Risk-L
TCGA-C9-A47Z	191	1	0.26256	Risk-H	TCGA-CV-7245	797	0	0.245605	Risk-H
TCGA-CV-A6JM	194	1	0.322456	Risk-H	TCGA-CR-5250	799	0	-0.49192	Risk-L
TCGA-CV-7438	194	1	0.336098	Risk-H	TCGA-CV-6940	804	1	0.222136	Risk-H
TCGA-CV-A6JO	197	1	0.128303	Risk-L	TCGA-CN-4730	817	0	0.296645	Risk-H
TCGA-CV-A45X	198	1	0.195435	Risk-H	TCGA-CR-7388	823	1	-0.01678	Risk-L
TCGA-CN-6020	205	1	0.268316	Risk-H	TCGA-BB-A5HZ	827	0	0.283975	Risk-H
TCGA-T2-A6WX	209	1	-0.08103	Risk-L	TCGA-QK-A6V9	833	0	-1.38201	Risk-L
TCGA-HD-A6I0	210	0	0.349531	Risk-H	TCGA-HD-7832	836	0	0.282891	Risk-H
TCGA-CV-5978	215	1	-0.00159	Risk-L	TCGA-CN-4740	839	1	0.039438	Risk-L
TCGA-CV-A465	215	1	0.181776	Risk-H	TCGA-BA-A6D8	850	0	0.181913	Risk-H
TCGA-BA-A6DB	216	0	-0.51815	Risk-L	TCGA-CV-A45O	851	0	0.19635	Risk-H
TCGA-T2-A6X0	216	0	-0.43322	Risk-L	TCGA-CV-A6JT	852	0	0.199934	Risk-H
TCGA-MZ-A5BI	217	1	-0.30745	Risk-L	TCGA-CN-6017	853	1	-0.43566	Risk-L
TCGA-CV-6956	217	1	0.330961	Risk-H	TCGA-BA-A4IG	855	0	-0.62452	Risk-L
TCGA-CV-7434	218	1	0.296957	Risk-H	TCGA-CV-6960	862	1	0.199748	Risk-H
TCGA-QK-A6IG	222	1	0.195592	Risk-H	TCGA-HD-7753	866	0	0.148055	Risk-H
TCGA-CN-A63T	225	0	0.239609	Risk-H	TCGA-D6-A6EK	875	0	0.346013	Risk-H
TCGA-D6-A6EM	232	0	0.303544	Risk-H	TCGA-P3-A6T5	882	1	0.272651	Risk-H
TCGA-CR-6487	234	0	-1.77132	Risk-L	TCGA-CR-7373	889	0	0.254702	Risk-H

TCGA-MT-A51X	242	0	0.190798	Risk-H	TCGA-CQ-A4CB	893	0	-0.01841	Risk-L
TCGA-CV-7099	243	1	0.150312	Risk-H	TCGA-BA-A4IF	895	0	-0.12957	Risk-L
TCGA-CN-5363	253	1	0.346109	Risk-H	TCGA-UF-A7JH	896	0	0.096987	Risk-L
TCGA-QK-AA3K	253	0	0.049956	Risk-L	TCGA-CQ-A4CE	897	0	0.14361	Risk-L
TCGA-CV-6959	256	1	0.192374	Risk-H	TCGA-CN-A49B	904	0	0.195577	Risk-H
TCGA-CN-5370	259	1	0.053232	Risk-L	TCGA-CV-A6JN	906	0	0.310633	Risk-H
TCGA-CN-5358	261	1	-0.53817	Risk-L	TCGA-P3-A5Q5	910	0	-0.49894	Risk-L
TCGA-BB-A6UO	268	1	0.29639	Risk-H	TCGA-CR-7402	911	0	-0.21392	Risk-L
TCGA-CV-7100	274	1	0.005144	Risk-L	TCGA-CR-7391	913	0	-0.20576	Risk-L
TCGA-BA-4078	276	1	-0.09141	Risk-L	TCGA-MT-A67A	914	0	0.221496	Risk-H
TCGA-CN-6021	276	1	0.227911	Risk-H	TCGA-CV-6951	915	1	0.260634	Risk-H
TCGA-BB-4224	278	0	-0.218	Risk-L	TCGA-BA-A4II	918	0	0.169293	Risk-H
TCGA-CR-7377	279	1	-0.40087	Risk-L	TCGA-CV-7568	927	1	0.08164	Risk-L
TCGA-CN-6022	281	1	0.082215	Risk-L	TCGA-CR-7395	930	0	-0.01003	Risk-L
TCGA-CR-6493	282	1	0.327594	Risk-H	TCGA-CN-6011	933	0	0.236736	Risk-H
TCGA-BA-4075	283	1	0.163437	Risk-H	TCGA-DQ-5629	941	1	0.280526	Risk-H
TCGA-QK-A6II	284	1	0.277577	Risk-H	TCGA-F7-A50J	947	0	0.208045	Risk-H
TCGA-CV-6441	292	1	0.147268	Risk-H	TCGA-CQ-A4CI	950	0	0.190862	Risk-H
TCGA-D6-6517	292	0	0.218626	Risk-H	TCGA-CN-A63U	964	0	0.203192	Risk-H
TCGA-CV-7413	294	1	0.239736	Risk-H	TCGA-CR-7376	972	0	-0.48905	Risk-L
TCGA-CV-6935	295	1	0.332858	Risk-H	TCGA-CN-6989	980	1	0.229821	Risk-H
TCGA-CR-6481	311	0	-2.09183	Risk-L	TCGA-CQ-6220	985	1	0.235972	Risk-H
TCGA-CQ-5332	317	1	0.232213	Risk-H	TCGA-T2-A6X2	987	0	0.099458	Risk-L
TCGA-CV-A6K2	317	1	0.202229	Risk-H	TCGA-CN-6997	988	1	0.144087	Risk-L
TCGA-CN-6988	318	0	0.184813	Risk-H	TCGA-CR-7393	993	0	-1.43419	Risk-L
TCGA-CX-7085	321	0	-0.228	Risk-L	TCGA-UF-A7JT	993	1	0.104254	Risk-L
TCGA-BB-A5HY	321	1	0.258545	Risk-H	TCGA-F7-8298	995	0	0.252564	Risk-H
TCGA-KU-A6H8	327	1	-0.01806	Risk-L	TCGA-CR-7385	997	0	-1.02826	Risk-L
TCGA-CV-7180	327	1	0.240804	Risk-H	TCGA-CN-4731	998	1	0.455548	Risk-H
TCGA-P3-A5QF	330	1	0.12407	Risk-L	TCGA-CQ-6221	1000	0	0.2558	Risk-H
TCGA-CV-6955	334	1	0.127618	Risk-L	TCGA-IQ-A61J	1021	0	0.063156	Risk-L
TCGA-BA-A6DI	336	1	0.196005	Risk-H	TCGA-CQ-A4CD	1022	0	0.212303	Risk-H
TCGA-CN-6024	337	1	0.31116	Risk-H	TCGA-HD-7229	1027	0	0.164002	Risk-H
TCGA-CQ-5333	341	1	0.374846	Risk-H	TCGA-DQ-5630	1030	0	-0.19073	Risk-L
TCGA-CV-6941	342	1	-0.0025	Risk-L	TCGA-CR-7379	1036	0	0.259995	Risk-H
TCGA-CR-6482	345	0	-1.38239	Risk-L	TCGA-CV-7422	1037	1	-0.14373	Risk-L
TCGA-D6-6826	348	1	-0.08157	Risk-L	TCGA-CN-6019	1038	0	0.02567	Risk-L
TCGA-BA-A6DA	351	0	0.294869	Risk-H	TCGA-CX-7219	1045	0	0.054445	Risk-L
TCGA-CN-5365	351	1	0.286984	Risk-H	TCGA-CR-6472	1050	0	0.039966	Risk-L
TCGA-CN-5367	352	1	0.166876	Risk-H	TCGA-HL-7533	1057	0	-0.03382	Risk-L
TCGA-CQ-A4C7	353	1	0.181965	Risk-H	TCGA-CN-A497	1065	0	0.087628	Risk-L
TCGA-CR-6484	354	0	-0.04814	Risk-L	TCGA-CN-6992	1066	0	0.059186	Risk-L

TCGA-CN-6998	357	1	0.335795	Risk-H	TCGA-CV-A6JE	1075	0	0.381683	Risk-H
TCGA-CR-5247	358	0	0.328017	Risk-H	TCGA-CR-7401	1077	0	0.19302	Risk-H
TCGA-F7-A622	359	1	0.314273	Risk-H	TCGA-CV-A45U	1079	1	-0.03303	Risk-L
TCGA-CN-5366	360	1	0.212235	Risk-H	TCGA-CV-7407	1081	1	0.045269	Risk-L
TCGA-IQ-A61G	360	0	0.259983	Risk-H	TCGA-CR-7369	1090	1	-0.06432	Risk-L
TCGA-CV-7253	361	1	0.361737	Risk-H	TCGA-CV-7446	1093	1	0.016819	Risk-L
TCGA-CR-6480	362	0	-0.23032	Risk-L	TCGA-CV-7242	1095	0	-0.28435	Risk-L
TCGA-CV-6945	366	1	0.306388	Risk-H	TCGA-P3-A6SW	1120	0	-0.18313	Risk-L
TCGA-CN-A641	367	0	0.237591	Risk-H	TCGA-CR-6473	1125	0	0.095242	Risk-L
TCGA-D6-A6EQ	368	0	0.241173	Risk-H	TCGA-F7-7848	1131	0	-0.38772	Risk-L
TCGA-QK-A8ZA	371	1	0.132884	Risk-L	TCGA-DQ-5625	1133	1	0.242636	Risk-H
TCGA-D6-A4ZB	376	0	0.196568	Risk-H	TCGA-BA-4077	1134	1	-0.09971	Risk-L
TCGA-CN-5359	377	1	-0.46371	Risk-L	TCGA-IQ-A61H	1138	0	0.167362	Risk-H
TCGA-CN-A63W	377	1	0.212762	Risk-H	TCGA-DQ-7592	1143	0	0.219338	Risk-H
TCGA-F7-A624	378	0	0.09891	Risk-L	TCGA-IQ-A61E	1147	0	0.219412	Risk-H
TCGA-CQ-A4CH	379	1	0.119243	Risk-L	TCGA-CR-5249	1152	0	-2.15351	Risk-L
TCGA-CR-6488	379	0	-0.17039	Risk-L	TCGA-CN-4725	1157	0	0.244978	Risk-H
TCGA-CN-5369	380	1	-0.02931	Risk-L	TCGA-IQ-7631	1172	0	0.252016	Risk-H
TCGA-MT-A67F	384	0	-0.52527	Risk-L	TCGA-CQ-6229	1179	0	0.177086	Risk-H
TCGA-BA-6872	384	1	0.303746	Risk-H	TCGA-CN-6994	1183	0	0.121817	Risk-L
TCGA-HD-8634	385	1	0.153694	Risk-H	TCGA-CR-7365	1191	0	0.247002	Risk-H
TCGA-CV-7097	385	1	0.221706	Risk-H	TCGA-CR-6471	1202	1	0.037487	Risk-L
TCGA-C9-A480	386	0	0.14129	Risk-L	TCGA-CR-7368	1245	0	-0.12818	Risk-L
TCGA-QK-A6IJ	387	0	0.299609	Risk-H	TCGA-CQ-6218	1253	0	0.233919	Risk-H
TCGA-RS-A6TO	387	1	0.149576	Risk-H	TCGA-BA-7269	1273	0	0.273311	Risk-H
TCGA-D6-A6ES	389	0	0.311107	Risk-H	TCGA-CQ-7069	1274	0	0.198517	Risk-H
TCGA-CR-7389	392	0	0.350088	Risk-H	TCGA-CN-5355	1278	0	0.243535	Risk-H
TCGA-QK-A8Z7	392	0	0.033368	Risk-L	TCGA-BA-5152	1288	0	0.242971	Risk-H
TCGA-CN-4729	392	0	0.146775	Risk-H	TCGA-CV-6948	1289	1	-0.0686	Risk-L
TCGA-BB-A6UM	393	0	-0.09804	Risk-L	TCGA-CQ-7068	1309	0	-0.1898	Risk-L
TCGA-CV-7104	393	1	0.186687	Risk-H	TCGA-CQ-7071	1311	0	0.201244	Risk-H
TCGA-CN-4736	395	1	-0.07014	Risk-L	TCGA-CV-5979	1315	0	0.262419	Risk-H
TCGA-P3-A6T6	395	1	0.092538	Risk-L	TCGA-CR-7394	1346	0	-0.23438	Risk-L
TCGA-CN-4742	397	1	0.276323	Risk-H	TCGA-CQ-A4C6	1353	0	0.13715	Risk-L
TCGA-P3-A6T8	400	0	0.220338	Risk-H	TCGA-UF-A7J9	1358	0	0.186379	Risk-H
TCGA-D6-6515	403	1	-0.37575	Risk-L	TCGA-CN-4739	1394	1	-0.39529	Risk-L
TCGA-TN-A7HJ	403	0	0.137755	Risk-L	TCGA-CV-A45W	1398	1	-0.19666	Risk-L
TCGA-CQ-6225	403	1	0.317566	Risk-H	TCGA-CQ-5331	1399	0	-0.08491	Risk-L
TCGA-KU-A66S	406	1	-0.22478	Risk-L	TCGA-CN-5356	1409	0	-0.10865	Risk-L
TCGA-CV-5970	406	1	0.275801	Risk-H	TCGA-CR-7392	1425	0	-0.20873	Risk-L
TCGA-H7-7774	407	0	0.162373	Risk-H	TCGA-CQ-6223	1428	0	0.236182	Risk-H
TCGA-BA-A6DJ	407	1	0.213874	Risk-H	TCGA-CR-7386	1430	0	0.04992	Risk-L

TCGA-TN-A7HI	412	0	0.13224	Risk-L	TCGA-P3-A6SX	1430	1	0.311446	Risk-H
TCGA-H7-A6C4	414	0	0.142708	Risk-L	TCGA-CR-7364	1435	0	0.099113	Risk-L
TCGA-BA-4076	415	1	0.279543	Risk-H	TCGA-CR-7367	1440	0	-0.01857	Risk-L
TCGA-T3-A92M	417	0	-0.05817	Risk-L	TCGA-CN-6016	1443	0	0.14878	Risk-H
TCGA-HD-A633	421	0	0.258411	Risk-H	TCGA-CV-7254	1459	1	-0.7697	Risk-L
TCGA-IQ-A61O	421	1	0.190412	Risk-H	TCGA-CN-6012	1460	0	0.197635	Risk-H
TCGA-D6-A6EP	424	0	0.242123	Risk-H	TCGA-UF-A71D	1461	0	0.074463	Risk-L
TCGA-UF-A7JK	424	1	0.286768	Risk-H	TCGA-CQ-5323	1466	0	0.02603	Risk-L
TCGA-DQ-7588	427	1	0.310943	Risk-H	TCGA-CV-A45Z	1466	1	-0.02267	Risk-L
TCGA-CQ-A4CG	430	1	0.21607	Risk-H	TCGA-CR-7404	1472	0	-0.87352	Risk-L
TCGA-CN-4738	436	1	-0.16583	Risk-L	TCGA-CV-5976	1478	0	0.2579	Risk-H
TCGA-MT-A51W	437	0	0.226267	Risk-H	TCGA-CN-4722	1483	0	0.182488	Risk-H
TCGA-BA-A6DE	440	0	0.246447	Risk-H	TCGA-UF-A71E	1504	1	0.276783	Risk-H
TCGA-IQ-7632	441	0	0.308592	Risk-H	TCGA-UF-A71B	1506	0	0.220432	Risk-H
TCGA-WA-A7H4	443	0	-0.07059	Risk-L	TCGA-CR-7390	1508	0	0.176552	Risk-H
TCGA-HD-8224	446	1	0.053964	Risk-L	TCGA-CV-7261	1512	0	0.181739	Risk-H
TCGA-QK-A8Z9	449	1	0.02473	Risk-L	TCGA-CR-6470	1521	0	-1.13852	Risk-L
TCGA-BA-6870	451	1	0.115434	Risk-L	TCGA-CN-6010	1523	0	-0.04628	Risk-L
TCGA-CV-7424	453	1	-0.13641	Risk-L	TCGA-CX-A4AQ	1555	0	-2.33725	Risk-L
TCGA-CQ-6228	456	1	0.04383	Risk-L	TCGA-P3-A5QE	1559	0	-1.78312	Risk-L
TCGA-H7-8502	458	0	0.24541	Risk-H	TCGA-CN-4727	1560	0	0.265891	Risk-H
TCGA-CV-6950	459	1	0.272142	Risk-H	TCGA-CN-5373	1584	0	-0.0685	Risk-L
TCGA-H7-8501	461	0	-0.38415	Risk-L	TCGA-CN-6023	1584	0	-0.80586	Risk-L
TCGA-BA-4074	462	1	0.282404	Risk-H	TCGA-CN-4733	1586	0	-0.29203	Risk-L
TCGA-CV-A468	464	1	-0.14868	Risk-L	TCGA-CV-7103	1591	1	0.214129	Risk-H
TCGA-QK-AA3J	466	0	0.056711	Risk-L	TCGA-CQ-5324	1593	0	0.268568	Risk-H
TCGA-MT-A7BN	469	0	0.069571	Risk-L	TCGA-CQ-7065	1628	0	0.294604	Risk-H
TCGA-IQ-A6SH	471	0	0.274056	Risk-H	TCGA-CV-6953	1641	1	0.284127	Risk-H
TCGA-BA-6868	472	1	0.200924	Risk-H	TCGA-CQ-5327	1660	0	0.146059	Risk-H
TCGA-CQ-6219	479	1	0.063959	Risk-L	TCGA-CR-5248	1663	0	-1.62385	Risk-L
TCGA-CR-6492	479	0	0.141148	Risk-L	TCGA-UF-A719	1663	0	0.224224	Risk-H
TCGA-P3-A5Q6	480	1	0.315683	Risk-H	TCGA-CV-6003	1665	0	0.300349	Risk-H
TCGA-T2-A6WZ	484	1	0.227509	Risk-H	TCGA-CV-7428	1671	1	0.086537	Risk-L
TCGA-IQ-7630	485	0	0.237678	Risk-H	TCGA-UF-A7JF	1686	0	0.052429	Risk-L
TCGA-P3-A6T7	487	1	0.250446	Risk-H	TCGA-CN-4734	1690	0	0.087	Risk-L
TCGA-MZ-A6I9	489	1	-0.06876	Risk-L	TCGA-CN-4723	1699	0	-0.01192	Risk-L
TCGA-D6-6825	491	0	-0.30269	Risk-L	TCGA-CV-7425	1718	1	0.148388	Risk-H
TCGA-CN-5364	493	1	0.149198	Risk-H	TCGA-CQ-6224	1721	0	0.246811	Risk-H
TCGA-CV-7430	495	1	0.299562	Risk-H	TCGA-CV-A464	1722	0	0.110079	Risk-L
TCGA-BA-A8YP	499	0	0.319185	Risk-H	TCGA-CN-4728	1724	0	0.008074	Risk-L
TCGA-CV-7437	506	1	0.229277	Risk-H	TCGA-CN-5374	1732	1	-1.13695	Risk-L
TCGA-CR-6477	514	0	0.211388	Risk-H	TCGA-CN-4735	1737	0	-0.46621	Risk-L

TCGA-RS-A6TP	516	0	0.086965	Risk-L	TCGA-CV-7406	1748	1	0.060828	Risk-L
TCGA-UP-A6WW	518	0	-0.18243	Risk-L	TCGA-BA-5153	1762	1	-0.68592	Risk-L
TCGA-BA-5555	520	0	0.078693	Risk-L	TCGA-CR-6467	1777	0	-1.42081	Risk-L
TCGA-CR-7383	521	1	0.06576	Risk-L	TCGA-DQ-5624	1778	0	0.210324	Risk-H
TCGA-CV-7248	521	1	-0.04019	Risk-L	TCGA-CV-A460	1838	1	-0.12374	Risk-L
TCGA-CV-5431	522	1	-0.16801	Risk-L	TCGA-CV-5977	1840	0	0.340707	Risk-H
TCGA-CN-A49A	526	1	-0.11522	Risk-L	TCGA-CQ-5330	1897	0	-0.13768	Risk-L
TCGA-CN-6996	530	1	-0.10499	Risk-L	TCGA-CV-6436	1899	0	0.389693	Risk-H
TCGA-D6-A4Z9	539	0	0.093553	Risk-L	TCGA-UF-A718	1971	0	0.162141	Risk-H
TCGA-QK-A8ZB	542	0	0.272063	Risk-H	TCGA-CV-7089	1972	1	0.204803	Risk-H
TCGA-F7-A620	543	0	-0.002	Risk-L	TCGA-BA-5558	1995	0	0.201361	Risk-H
TCGA-CV-5966	545	1	0.384122	Risk-H	TCGA-CV-6954	2002	1	0.264889	Risk-H
TCGA-CV-5439	546	1	0.116887	Risk-L	TCGA-CQ-6222	2016	0	0.100711	Risk-L
TCGA-UF-A7JC	546	1	0.27501	Risk-H	TCGA-CV-A461	2064	1	-0.19019	Risk-L
TCGA-MZ-A7D7	547	0	0.246413	Risk-H	TCGA-BA-5559	2083	1	-1.12355	Risk-L
TCGA-DQ-5631	548	1	0.201782	Risk-H	TCGA-CN-5361	2120	1	0.117182	Risk-L
TCGA-UF-A7JJ	549	0	-0.24453	Risk-L	TCGA-CQ-7063	2133	0	-0.16866	Risk-L
TCGA-KU-A66T	552	0	0.279625	Risk-H	TCGA-CQ-5329	2143	0	0.229799	Risk-H
TCGA-BB-4228	559	0	-0.13885	Risk-L	TCGA-BB-8596	2161	0	-0.3028	Risk-L
TCGA-CV-7263	560	1	-0.0026	Risk-L	TCGA-CV-7178	2166	1	-0.00666	Risk-L
TCGA-CR-6474	564	1	0.141479	Risk-L	TCGA-CN-5360	2169	0	-0.58004	Risk-L
TCGA-D6-6827	568	0	-0.5081	Risk-L	TCGA-P3-A5QA	2182	0	0.182603	Risk-H
TCGA-CV-7095	572	1	0.325461	Risk-H	TCGA-CN-4741	2239	0	0.055676	Risk-L
TCGA-CX-7086	573	0	0.125423	Risk-L	TCGA-UF-A7JA	2265	0	0.132637	Risk-L
TCGA-F7-A61S	576	0	0.20375	Risk-H	TCGA-P3-A6T2	2298	0	0.294898	Risk-H
TCGA-CV-7247	577	1	0.259078	Risk-H	TCGA-CV-5435	2319	1	0.216409	Risk-H
TCGA-P3-A6T3	577	1	0.25783	Risk-H	TCGA-CV-5442	2327	0	0.188936	Risk-H
TCGA-P3-A6T0	578	0	0.26217	Risk-H	TCGA-CV-7235	2347	0	0.062476	Risk-L
TCGA-IQ-A6SG	579	0	0.320211	Risk-H	TCGA-CQ-7072	2359	0	0.067549	Risk-L
TCGA-CN-6018	580	1	0.2146	Risk-H	TCGA-CV-5444	2437	0	0.046028	Risk-L
TCGA-CV-5436	584	1	0.273833	Risk-H	TCGA-CR-5243	2562	0	-0.36903	Risk-L
TCGA-KU-A6H7	586	0	-0.20245	Risk-L	TCGA-CV-7432	2570	1	0.295193	Risk-H
TCGA-CN-A6V7	594	0	0.010107	Risk-L	TCGA-CV-5973	2641	0	0.339052	Risk-H
TCGA-QK-A6VC	600	0	-0.22267	Risk-L	TCGA-CV-A45Y	2703	1	0.039637	Risk-L
TCGA-CV-7433	601	1	0.213842	Risk-H	TCGA-CV-7411	2717	1	0.293066	Risk-H
TCGA-CV-6943	602	1	-0.14478	Risk-L	TCGA-CV-7238	2727	0	-0.14869	Risk-L
TCGA-CR-7380	606	1	-0.04313	Risk-L	TCGA-CV-6933	2741	1	0.200041	Risk-H
TCGA-CV-A6K0	606	0	-0.64403	Risk-L	TCGA-CV-5443	2784	0	-0.24348	Risk-L
TCGA-F7-A50G	616	0	-0.02757	Risk-L	TCGA-CV-5441	2886	0	0.260344	Risk-H
TCGA-F7-A623	616	0	0.061505	Risk-L	TCGA-CV-7250	2900	1	0.101084	Risk-L
TCGA-TN-A7HL	619	0	-0.36303	Risk-L	TCGA-CV-7423	3059	1	0.268307	Risk-H
TCGA-BA-A4IH	622	0	-0.14764	Risk-L	TCGA-BB-4223	3221	0	-1.92222	Risk-L

TCGA-DQ-7591	622	0	-1.37204	Risk-L	TCGA-CV-5440	3270	0	0.24818	Risk-H
TCGA-BA-5557	623	0	0.126484	Risk-L	TCGA-CV-5434	3314	1	0.088661	Risk-L
TCGA-BA-A6DL	623	0	0.204936	Risk-H	TCGA-CV-7091	3381	0	0.180802	Risk-H
TCGA-BB-8601	624	0	0.309753	Risk-H	TCGA-CV-5432	3930	0	0.272345	Risk-H
TCGA-CV-6937	624	1	0.234928	Risk-H	TCGA-CV-7183	3981	0	0.32282	Risk-H
TCGA-CN-4737	625	0	0.028854	Risk-L	TCGA-CV-5430	4241	0	-0.33973	Risk-L
TCGA-WA-A7GZ	625	1	0.189498	Risk-H	TCGA-CV-6942	4282	0	-0.46084	Risk-L
TCGA-UF-A7JO	631	1	-0.26414	Risk-L	TCGA-CV-7435	4680	1	0.203096	Risk-H
TCGA-CN-A6V6	635	0	-0.7327	Risk-L	TCGA-CV-7427	4760	1	-0.02354	Risk-L
TCGA-H7-A76A	637	0	-1.27383	Risk-L	TCGA-CV-A45T	4856	1	-0.64943	Risk-L
TCGA-CV-A45P	639	0	0.209929	Risk-H	TCGA-CV-A45Q	5152	1	0.346233	Risk-H
TCGA-CV-6433	641	0	-0.10246	Risk-L	TCGA-CV-7090	5252	0	0.170112	Risk-H
TCGA-QK-A64Z	641	1	0.202539	Risk-H	TCGA-CV-A45R	5480	0	0.120086	Risk-L
TCGA-QK-A6VB	641	0	0.140043	Risk-L	TCGA-CV-7410	6417	1	-3.9063	Risk-L
TCGA-BA-6869	644	0	0.188739	Risk-H					

Table S9: The risk coefficient of PC1 in three groups of signature Gs

Coef.gName	Coef.Coef
signature G3 PC1	-0.7631
signature G2 PC1	0.256376
signature G1 PC1	0.070339

Table S10: Details of 26 genes with high frequency mutation

gName	FisherP	Mut_TMEscoreL	Nor_TMEscoreL	Mut_TMEscoreH	Nor_TMEscoreH
TP53	9.69E-08	138	107	194	52
ABCA10	0.000871	10	235	0	246
CYLD	0.001593	12	233	1	245
SCLT1	0.001779	9	236	0	246
FGFR3	0.001779	9	236	0	246
IQUB	0.003043	11	234	1	245
SLTM	0.003626	8	237	0	246
MASP1	0.003626	8	237	0	246
OR2T6	0.003626	8	237	0	246
ZNF750	0.003629	13	232	2	244
ACTN2	0.005772	10	235	1	245
C8B	0.005772	10	235	1	245
IGSF1	0.005772	10	235	1	245
NLRP12	0.006064	16	229	4	242
RP1L1	0.00653	14	231	3	243
ATAD2	0.007374	7	238	0	246

HNRNPU	0.007374	7	238	0	246
GPRASP2	0.007374	7	238	0	246
SPATA7	0.007374	7	238	0	246
KRT74	0.007374	7	238	0	246
C11orf30	0.007374	7	238	0	246
OR2C3	0.007374	7	238	0	246
MTPAP	0.007374	7	238	0	246
ESYT3	0.007374	7	238	0	246
CEP152	0.007374	0	245	8	238
AKAP9	0.0084	22	223	8	238
